# Diastereoselective multicomponent synthesis of dihydroisoindolo[2,1-*a*]quinolin-11-ones mediated by eutectic solvents†

**DOI:** 10.1039/d3ra05561b

**Published:** 2023-09-04

**Authors:** Carlos M. Sanabria-Sánchez, Vladimir V. Kouznetsov, Cristian Ochoa-Puentes

**Affiliations:** a Laboratorio de Química Orgánica y Biomolecular, Escuela de Química, Universidad Industrial de Santander Cl. 9 # Cra 27 A.A. 680006 Bucaramanga Santander Colombia; b Laboratorio de Síntesis Orgánica Sostenible, Departamento de Química, Universidad Nacional de Colombia-Sede Bogotá Carrera 45 # 26-85 A.A. 5997 Bogotá Cundinamarca Colombia cochoapu@unal.edu.co

## Abstract

In this contribution, a series of dihydroisoindolo[2,1-*a*]quinolin-11-ones was synthesized by a one-pot multicomponent Povarov reaction starting from anilines, alkenes (*trans*-anethole, methyl eugenol and indene) and 2-formylbenzoic acid. Different eutectic solvents bearing Lewis or Brønsted acids were evaluated as reaction media and catalysts for the model reaction employing *p*-toluidine and *trans*-anethole finding that the eutectic mixture ChCl/ZnCl_2_ (1/2) allowed the obtention of the target compound in 77% isolated yield. Under the optimized reaction conditions, 20 derivatives were obtained in good to moderated yields using *meta*- and *para*-susbstituted anilines, while the corresponding *ortho*-analogs followed a different pathway affording isoindolinones. In addition, the eutectic mixture was reused in six cycles without observing a detrimental catalytic activity. This methodology features mild reaction conditions, short reaction time, simple work-up, and utilization of a reusable solvent; and provides straightforward and diastereoselective access to these alkaloid-like heterocyclic molecules.

## Abstract 2

A convenient, fast and environmentally friendly synthesis of isoindolo[2,1-*a*]quinolin-11-one derivatives has been developed employing choline chloride/zinc chloride eutectic solvent as alternative reaction media.

## Graphical abstract

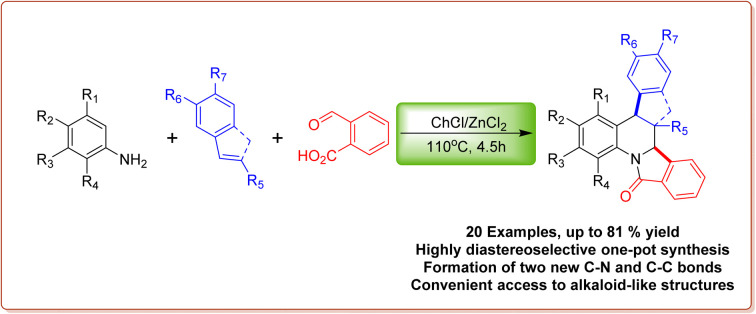

## Introduction

The introduction of alternative solvents in organic synthesis during the last decades has offered a new platform to explore classical and contemporary reactions. Although the main goal is the reduction of the negative environmental impact of petroleum-derived solvents, new perspectives have emerged with solvents such as ionic liquids and deep eutectic solvents (DES) due to their capability to promote different types of reactions.^1–4^ However, DES are considered to be superior to ionic liquids because they are easily prepared, dissolve a wide scope of substrates, can be readily recycled and reused, are air and moisture stable, and can play different roles as solvents, reagents and catalyst.^5,6^ A DES can be defined as a mixture, formed by two or more compounds, with the lowest melting temperature compared to its individual components. To date, these solvents have been classified considering their components: DES type I are formed from metal chlorides (MCl_*x*_) and quaternary ammonium salts; type II are based on metal chloride hydrates and quaternary ammonium salts; hydrogen bond donors (HBD) and quaternary ammonium salts form the type III; HBD and metal chloride hydrates are the type IV and finally, mixtures of non-ionic compounds such as terpenes or terpenoids constitute the type V.^7^ Notable applications of DES for the synthesis of heterocyclic compounds,^8^ C–H activation, and cross-coupling reactions^9^ have been recently reported in literature, and although several groups have made important contributions in these fields, the scope and limitations of these new and promising reaction media are still under study.

**Scheme 1:**
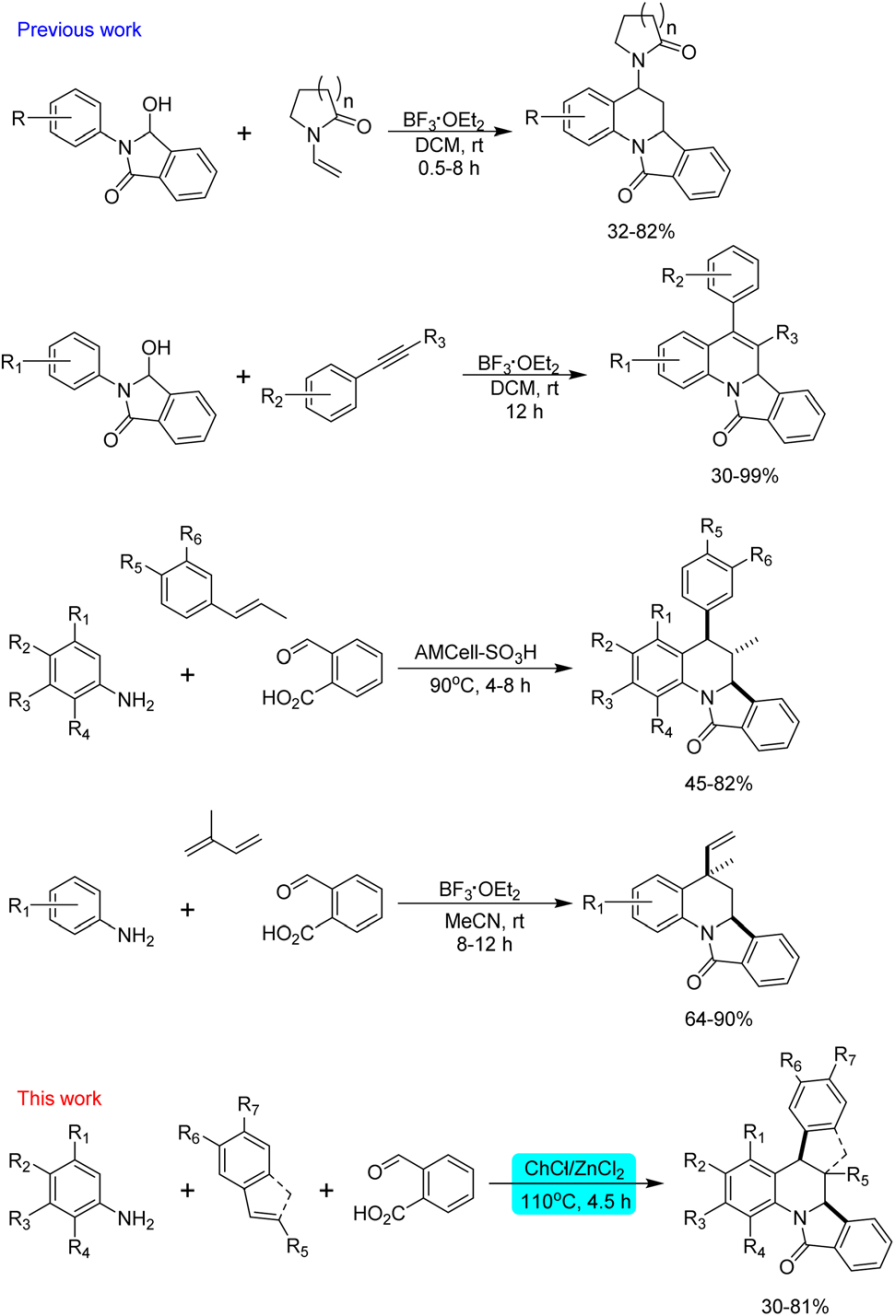
Imino Diels–Alder reaction in the synthesis of isoindolo[2,1-*a*]quinolin-11-ones.

Multicomponent reactions are one of the most versatile and atom-economy strategies for the construction of diverse molecular structures. This synthetic methodology, in combination with the use of heterogeneous and easily recoverable catalysts, alternative solvents and microwave irradiation, has promoted the preparation of interesting compounds with broad applications in medicinal chemistry.^10–12^ Among the known multicomponent reactions, the inverse electron-demand aza Diels–Alder approach (IEDDA reaction) has been of great interest for synthetic chemists because this synthetic tool allows the obtention of quinoline derivatives highly functionalized considering the wide variety of functional groups that can be attached to the aromatic amine, the aldehyde and the dienophile. Recently, numerous efforts in the IEDDA reaction directed toward the preparation of asymmetric derivatives^13–17^ and ring-fused derivatives have been reported.^18^ While the stereoselective synthesis of tetrahydroquinolines is usually performed with chiral catalysts such as Lewis acids,^19,20^ phosphoric acid,^21–23^ and proline derivatives,^24^ ring-fused derivatives of tetrahydroquinolines can be easily and conveniently obtained by the Povarov reaction employing cyclic dienophiles such as cyclopropene,^25^ 2,3-dihydrofuran,^26^ 2,3-dihydropyrrole,^27,28^ 3,4-dihydropyran,^29,30^ cyclopentadiene,^31,32^ indene,^33^ benzofuran^34^ and indole^35^ employing Lewis or Brønsted acid as catalysts. A double Povarov reaction has been also considered to synthesize julolidine derivatives employing TFA, TFE,^36^ silica sulphuric acid and *p*-sulfonic acid calix[4]arene as catalysts.^37,38^ However, a more well-designed and developed methodology to construct these polycyclic structures involves the use of functional amines, aldehydes or dienophiles which can undergo consecutive reactions in a one-pot fashion. Following this approach, a series of furo[3,2-*c*]pyrrolo[1,2-*a*]quinoline derivatives were obtained starting from homopropargylic amines which undergo an intramolecular hydroamination and cycloisomerization forming a 2-azadiene which react with electron-rich olefins.^39^ On the other hand, the synthesis of [1,2-*a*]-fused tetrahydroquinolines can be performed *via* intramolecular aza-Diels–Alder reaction of *ortho*-quinone methide imines tethered to electron-neutral dienophiles,^18^ or by a dual C–H functionalization of *N*-aryl tetrahydroisoquinolines *via* copper(i) bromide catalysed oxidation.^40^ The intramolecular aza-Diels–Alder reaction between *N*-prenylated 1-formyl-9*H*-β-carbolines and substituted anilines in the presence of Yb(OTf)_3_ allowed the obtention of dihydroquinoline-fused canthines;^41^ while *O*-propargylated and *O*-cinnamyl salicylaldehydes in combination of indolines and anilines have been used to synthesize cromenoquinolines employing catalysts such as chiral amines,^42,43^ chiral phosphoric acid,^44^ I_2_,^45^ Ru(bpy)_3_(PF_6_)_2_,^46^ and CuI/La(OTf)_3._^47^ Furthermore, the *in situ* formation of a dienophile by a one-pot gold-catalysed hydroalkoxylation of alkynols and subsequent Povarov reaction with *N*-aryl imines or *in situ* generated iminium has been developed for the obtention of [3,2-*c*]quinoline derivatives.^48^ Finally, the synthesis of isoindolo[2,1-*a*]quinolin-11-ones have been achieved by cyclization from *N*-aryl-3-hydroxyisoindolinones and *N*-vinyl lactams or aryl alkynes under BF_3_·Et_2_O catalysed reaction,^49–51^ and by the reaction between anilines, dienophiles and 2-formylbenzoic acid (Scheme 1).^52,53^

All these synthetic efforts demonstrate the utility of the imino-Diels–Alder reaction in the obtention of ring-fused tetrahydroquinoline derivatives, however, environmental concerns regarding the impact of solvents and catalysts employed in this reaction demand the development of new methodologies. Motivated by this, herein we show our results directed to the obtention of dihydroisoindolo[2,1-*a*]quinolin-11(5*H*)-ones employing eutectic solvents as alternative and environmentally friendly reaction media (Scheme 1).

## Results and discussion

Considering that the straightforward method for the synthesis of the target compounds involves the Povarov reaction using of 2-formylbenzoic acid and the remarkable effect of deep eutectic solvents on this reaction,^54^ our initial efforts started with the screening of several acidic deep eutectic solvents in the model reaction between *p*-toluidine, *trans*-anethole and 2-formylbenzoic acid (Table 1).

**Table 1:** Screening of different eutectic mixtures for the obtention of the isoindoloquinoline 1

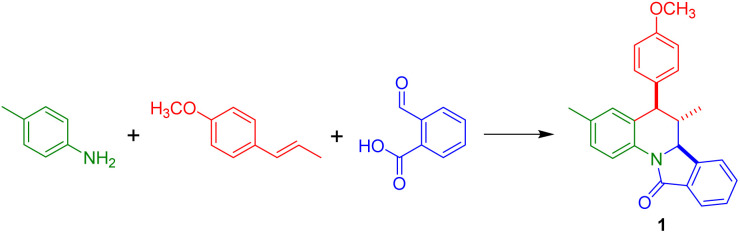		
Entry	Reaction conditions	Yield^a^ (%)
1	ChCl/ZnCl_2_ (1/2), 110 °C, 3 h^b^	60
2	ChCl/ZnCl_2_ (1/2), 110 °C, 4.5 h^b^	68
3	ChCl/ZnCl_2_ (1/2), 110 °C, 4.5 h^c^	77
4	ChCl/ZnCl_2_ (1/2), MW 150 °C, 16 min^c^	40
5	Urea/ZnCl_2_ (3.5/1), 110 °C, 24 h^c^	0
6	ChCl/MgCl_2_·6H_2_O (1/2), 110 °C, 24 h^c^	0
7	ChCl/SnCl_2_ (1/2), 110 °C, 4.5 h^c^	49
8	ZnCl_2_/ethylene glycol (1/4), 110 °C, 4.5 h^c^	49
9	ChCl/APTS (1/2), 110 °C, 4.5 h^c^	65
10	ChCl/CF_3_SO_3_H, 110 °C, 4.5 h^c^	64
11	AMCell-SO_3_H, 90 °C, 5 h	68 (ref. 53)

a Isolated yield.

b *p*-Toluidine (1 mmol), *trans*-anethole (1 mmol), 2-formylbenzoic acid (1 mmol).

c *p*-Toluidine (1 mmol), *trans*-anethole (1.1 mmol), 2-formylbenzoic acid (1.2 mmol).

According to Table 1, the eutectic mixture ChCl/ZnCl_2_ afforded the target compound in 60 and 68% yield after 3 and 4.5 h respectively using equimolar amounts of reactants (entries 1 and 2); however, with a slight increase in the dienophile and 2-formylbenzoic acid the yield increases up to 77% (entry 3). The reaction performed at higher temperature employing a microwave reactor gives a complex mixture where 40% of the product was isolated (entry 4). In contrast, the eutectic mixtures urea/ZnCl_2_ (3.5/1) and ChCl/MgCl_2_·6H_2_O (1/2) did not afford the compound even under prolonged reaction times (entries 5 and 6). The Lewis acid based eutectic mixtures ChCl/SnCl_2_ (1/2) and ZnCl_2_/ethylene glycol (1/4) yielded the isoindoloquinoline 1 in 49% (entries 7 and 8), while the eutectic mixtures based on Brønsted acids ChCl/APTS (1/2) and ChCl/CF_3_SO_3_H give the compound in 65 and 64% yield respectively (entries 9 and 10). The results here obtained showed that the isoindoloquinoline 1 can be easily obtained in a better yield in comparison to a previous reported method (entry 11).^53^^1^H NMR analysis of the compound indicates that the compound was obtained as a single diastereomer (6,6*a-trans*-isomer) as confirmed by the coupling constants found for the vicinal protons 5-H and 6-H (*J* = 11.0 Hz) as well as for 6-H and 6a-H (*J* = 10.7 Hz). The magnitude of these coupling constants agrees with an axial–axial relationship between 5-H and 6-H.

**Scheme 2:**
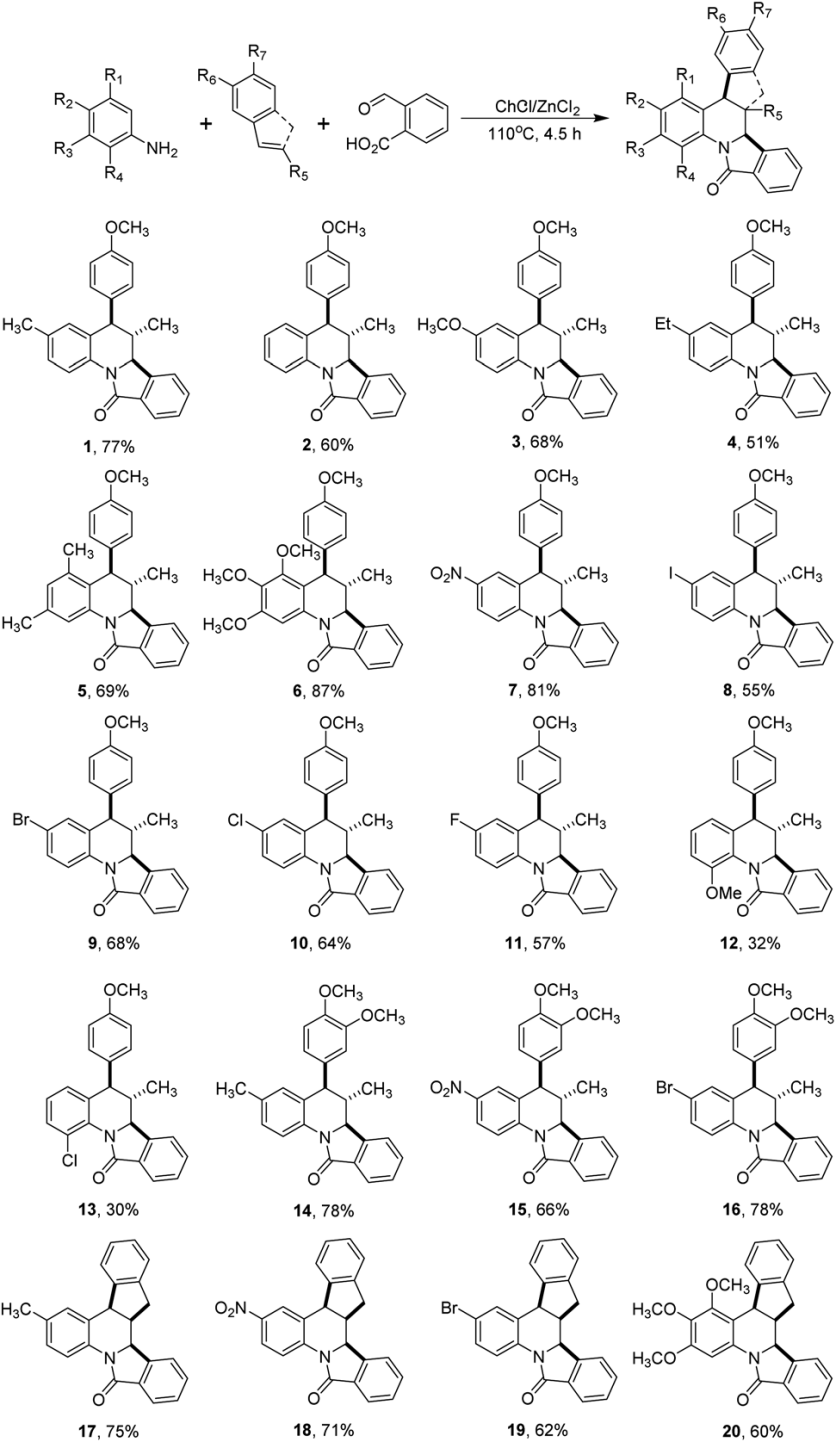
Substrate scope of the reaction.

With these results in our hands, we decided to study the scope of this methodology employing different anilines and dienophiles (Scheme 2).

As depicted in Scheme 2, under the optimal reaction conditions found in Table 1 the isoindoloquinolines 1–11 were obtained in yields between 51 and 87% employing *trans*-anethole, aniline and its *meta*- and *para*-substituted analogues bearing electron-donating and electron-withdrawing groups. According to the results, we did not observe a close correlation between the electronic nature of the substituents on the aniline and the yield, however, when *ortho*-substituted anilines were used a complex mixture was obtained and the desired compounds 12 and 13 could be isolated in low yields (32 and 30%, respectively). Other dienophiles such as methyl isoeugenol and indene also afforded the target compounds 14–20 in yields comparable to that obtained with *trans*-anethole. The indenoisoindoloquinolines 17–20 were also obtained as a single regio- and diastereoisomer where the indene and isoindole rings have a *cis* orientation having coupling constants of 9.1–8.1 Hz for the vicinal protons H-10b and H-15a; and 3.8–3.6 Hz for protons H-15b and H-15a.

**Scheme 3:**
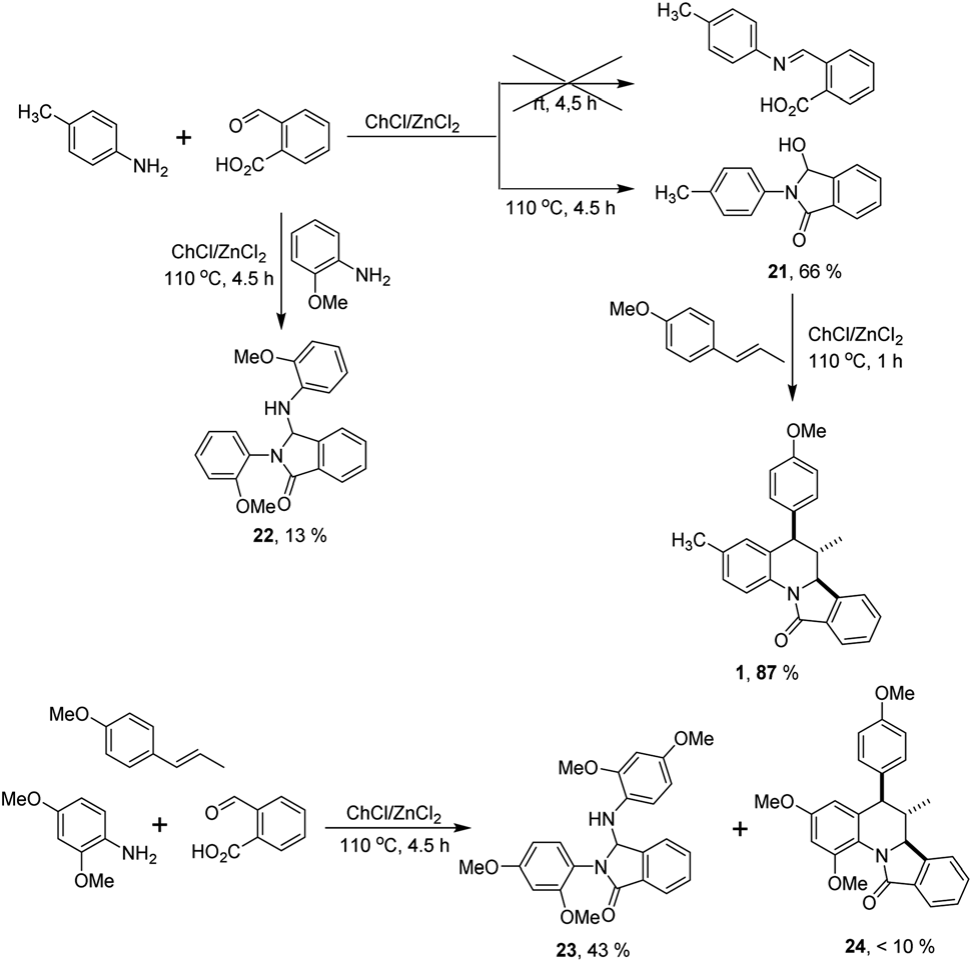
Experiments conducted to propose a plausible reaction mechanism for the obtention of the isoindoloquinolines 1–20.

To understand the different reactivity observed in the reactions performed with *ortho*-substituted anilines, and to get inside of the mechanistic pathway a set of experiments were performed (Scheme 3). It has been proposed that the reaction between anilines and 2-formylbenzoic acid in the Povarov reaction may led to the formation of an imine or and isoindolinone^52,53^ which further undergo the cycloaddition step to assemble the isoindoloquinoline skeleton. In our hands, *p*-toluidine and 2-formylbenzoic acid did not react employing the DES at room temperature, however when the reaction was conducted under the optimal conditions the 3-hydroxy-isoindolinone 21 was isolated in 66% yield. In contrast, when the same reaction was performed with *o*-anisidine, the corresponding 3-hydroxy-isoindolinone was not obtained and instead of this the 3-substituted isoindolinone 22 was isolated in 13% yield together with unreacted starting materials (Scheme 3). These observations suggest that the formation of the isoindoloquinoline occurs *via* intermediate 21, but in the case of *ortho*-substituted anilines the cycloaddition is not favoured because the required cisoid geometry of the *N*-acyliminium cation is probably not reached due to steric hindrance of the *ortho* substituent on the *N*-aryl^49^ and the alkylation with other equivalent of the amine takes place.^55^ To confirm our hypothesis, compound 21 was further poured into the reaction together with *trans*-anethole and the eutectic mixture obtaining the isoindoloquinoline 1 in 87% yield. This observation was also noted in the multicomponent reaction between 2,4-dimethoxyaniline, *trans*-anethole and 2-formylbenzoic acid were the product of alkylation 23 was isolated in 43% yield and the diagnostic signals for the isoindoloquinoline 24 (H-6, H-6a and CH_3_) were identified in the ^1^H NMR spectra of a complex fraction obtained after purification (Scheme 3).

**Scheme 4:**
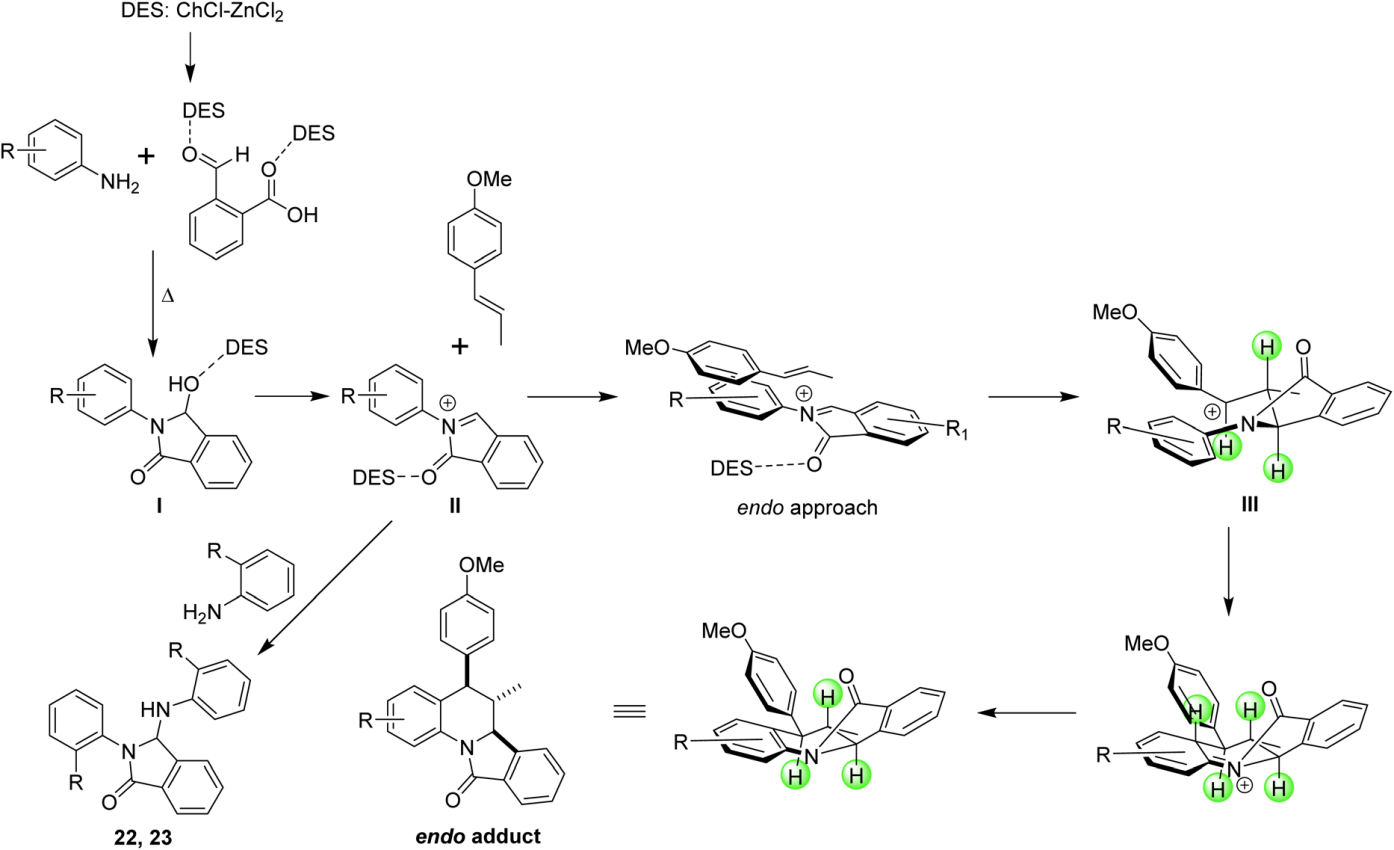
Plausible reaction mechanism for the obtention of isoindoloquinolines 1–20 and 3-substituted isoindolinones 22 and 23.

With the information provided by the experiments previously performed a plausible reaction mechanism was proposed (Scheme 4). We believe that the reaction starts with the formation of the 3-hydroxy-isoindolinone I which undergo a de-hydroxylation, mediated by the eutectic mixture, to form an *N*-acyliminium cation II (2-aza-diene). Next, the electrophilic addition of this cation over the dienophile (*trans*-anethole, methyl isoeugenol or indene) generates the benzylic cation III which after an intramolecular Friedel–Crafts reaction afford the isoindoloquinoline. This proposed mechanism supports the diastereosectivity observed in the product because no evidence of an *exo* adduct was observed, and also explain the formation of compounds 22 and 23 that may be formed through the amidoalkylation of 3-hydroxy-isoindolinones obtained from *ortho*-substituted anilines. From the proposed mechanism can be suggested that the deep eutectic solvent places an important role facilitating the formation of the intermediate I, its de-hydroxylation and alkylation, and enhance the electrophilic character of the *N*-acyliminium cation II.

Once the synthetic utility of the eutectic mixture for the obtention of the target compounds was demonstrated, we directed our efforts on study the recyclability and reusability of this versatile reaction media, and for this, after completion of the model reaction 2 mL of water were added and then the crude solid product was isolated by vacuum filtration. The DES was recovered from the filtrate by lyophilisation and added with new reactants for another run. This process was repeated several times obtaining the isoindoloquinoline 1 in 75, 73, 72, 69, 63 and 51% yield. These results indicate that the eutectic mixture can be easily recovered and reused several times without a remarkable negative effect on the outcome of the reaction for at least four cycles. Scaling up key molecules for biomedical or technological applications usually represents a challenge; therefore, in order to validate our methodology for its further implementation at large scale the model reaction for obtaining the derivative 1 was scaled-up (10 mmol of *p*-toluidine) under the optimized conditions obtaining the corresponding isoindoloquinoline in 75% yield.

The easy isolation of the crude product, the recyclability and reusability of the DES together with the scalability of this methodology for the preparation of the target compound are key factors that highlight the benefits of this synthetic procedure over other reported methodologies.^49–53^

## Experimental

### Materials and methods

All the chemicals and solvents were purchased from commercial suppliers (Aldrich, Merck) and used as received without further purification except for choline chloride (ChCl), which was dried at 130 °C in vacuum and stored under nitrogen prior to use. Melting points, reported without correction, were measured using a Fisher–Jhones apparatus. The Fourier transform infrared spectra were obtained using a Shimadzu IR prestige 21 spectrophotometer (Columbia, MD, USA). ^1^H and ^13^C NMR spectra were recorded using a Bruker AVANCE III system operating at 400 MHz, using residual and deuterated solvent peaks of CDCl_3_ (*δ*_H_ 7.26; *δ*_C_ 77.0) and DMSO (*δ*_H_ 2.50; *δ*_C_ 39.5) as reference standards. The peak patterns are indicated as follows: s, singlet; d, doublet; t, triplet; m, multiplet; and q, quartet. The coupling constants, *J*, are reported in hertz (Hz).

### DES preparation

The DESs were formed by mixing the components in the respective molar ratio (Table 1) in a glass vial. The mixtures were heated up to 80 °C until a homogeneous colourless liquid was obtained, except for DES ChCl/ethylene glycol and ChCl/CF_3_SO_3_H.

### General experimental procedure for the synthesis of isoindoloquinolines 1–20

To an open headspace vial equipped with a magnetic stir bar, 2.88 g of ChCl/ZnCl_2_ DES (1 : 2) (0.98 g, 7 mmol of ChCl and 1.90 g, 14 mmol of ZnCl_2_) was added and heated to 80 °C to obtain a clear melt. To this melt, a mixture of substituted aniline (1 mmol), alkene (*trans*-anethole, methyl isoeugenol or indene, 1.1 mmol) and 2-formylbenzoic acid (1.2 mmol) was added, and the reaction mixture was stirred at 110 °C for 4.5 h. After completion of the reaction (monitored by TLC), the reaction mixture was quenched by adding water while still hot and cooled to room temperature, and the crude solid was filtered, washed with water, and purified by column chromatography on silica gel (60–120 mesh) using a mixture of petroleum ether–ethyl acetate as an eluent to afford the tetrahydroquinoline derivatives.

#### *Trans*-5-(4-methoxyphenyl)-3,6-dimethyl-6,6*a*-dihydro-isoindolo[2,1-*a*]quinolin-11(5*H*)-one (1)

White solid, mp 183–184 °C. Yield 77%. IR (KBr): *ν*_max_ 2997, 1683, 1495, 1380, 1246, 832, 739 cm^−1^; ^1^H NMR (400 MHz, CDCl_3_): *δ* (ppm) 8.36 (d, *J* = 8.3 Hz, 1H), 8.01–7.95 (m, 1H), 7.64–7.60 (m, 1H), 7.56 (td, *J* = 7.2, 1.5 Hz, 1H), 7.54–7.50 (m, 1H), 7.08 (m, 1H), 7.04 (d, *J* = 8.7 Hz, 2H), 6.85 (d, *J* = 8.7 Hz, 2H), 6.62–6.56 (m, 1H), 4.48 (d, *J* = 10.7 Hz, 1H), 3.80 (s, 3H), 3.76 (d, *J* = 11.0 Hz, 1H), 2.17 (s, 3H), 1.91–1.82 (m, 1H), 1.24 (d, *J* = 6.5 Hz, 3H); ^13^C NMR (101 MHz, CDCl_3_): *δ* (ppm) 165.6, 158.5, 143.2, 135.9, 133.6, 133.4, 133.2, 131.4, 130.6, 130.6, 130.5, 128.6, 127.8, 124.3, 124.2, 120.2, 114.0, 64.1, 55.3, 52.2, 42.0, 21.1, 16.2. GC-MS (EI) *m*/*z*: 369.2 (M^+^); anal. calcd for C_25_H_23_NO_2_: C, 81.27; H, 6.28; N, 3.79; O, 8.66. Found: C, 81.23; H, 6.26; N, 3.71.

#### *Trans*-5-(4-methoxyphenyl)-2,4,6-trimethyl-6,6*a*-dihydroisoindolo[2,1-*a*]quinolin-11(5*H*)-one (5)

White solid, mp 178–180 °C. Yield 69%. IR (ATR): *ν*_max_ 2971, 1685, 1507, 1379, 1242, 833, 742 cm^−1^. ^1^H NMR (400 MHz, CDCl_3_): *δ* (ppm) 8.12 (d, *J* = 1.9 Hz, 1H), 7.99–7.95 (m, 1H), 7.60–7.57 (m, 1H), 7.52 (m, 2H), 6.96 (d, *J* = 8.7 Hz, 2H), 6.74 (d, *J* = 8.7 Hz, 2H), 6.71 (d, *J* = 1.9 Hz, 1H), 4.34 (d, *J* = 11.2 Hz, 1H), 3.74 (s, 3H), 3.72 (d, *J* = 9.5 Hz, 1H), 2.35 (s, 3H), 1.79 (s, 3H), 1.74–1.68 (m, 1H), 1.38 (d, *J* = 6.6 Hz, 3H). ^13^C NMR (101 MHz, CDCl_3_): *δ* (ppm) 165.3, 158.0, 143.6, 138.0, 137.3, 136.9, 136.5, 133.4, 131.5, 129.9, 128.6, 128.5, 126.7, 124.4, 124.0, 118.9, 113.8, 63.5, 55.3, 50.3, 45.7, 21.3, 21.3, 16.6. GC-MS (EI) *m*/*z*: 383.2 (M^+^); anal. calcd for C_26_H_25_NO_2_: C, 81.43; H, 6.57; N, 3.65; O, 8.34. Found: C, 80.96; H, 6.51; N, 3.60.

#### *Trans*-5-(3,4-dimethoxyphenyl)-3,6-dimethyl-6,6*a*-dihydroisoindolo[2,1-*a*]quinolin-11(5*H*)-one (14)

White solid, mp 218–219 °C. Yield 78%. IR (ATR): *ν*_max_ 3077, 2989, 1678, 1517, 1383, 1233, 880, 747 cm^−1^. ^1^H NMR (400 MHz, CDCl_3_): *δ* (ppm) 8.35 (d, *J* = 8.3 Hz, 1H), 7.99 (dd, *J* = 7.6, 1.2 Hz, 1H), 7.66–7.60 (m, 1H), 7.60–7.55 (m, 1H), 7.55–7.51 (m, 1H), 7.09 (dt, *J* = 8.3, 1.0 Hz, 1H), 6.83 (d, *J* = 8.2 Hz, 1H), 6.77 (dd, *J* = 8.2, 2.0 Hz, 1H), 6.65–6.57 (m, 1H), 6.52 (d, *J* = 2.0 Hz, 1H), 4.50 (d, *J* = 10.7 Hz, 1H), 3.89 (s, 3H), 3.76 (s, 3H), 2.18 (s, 3H), 1.95–1.82 (m, 1H), 1.25 (d, *J* = 6.5 Hz, 3H). ^13^C NMR (101 MHz, CDCl_3_): *δ* (ppm) 165.8, 149.4, 148.1, 143.3, 136.2, 133.8, 133.4, 133.2, 131.5, 130.56, 130.4, 128.8, 127.9, 124.4, 124.3, 122.5, 120.2, 111.7, 110.9, 64.2, 56.1, 56.0, 52.9, 41.8, 21.1, 16.4. GC-MS (EI) *m*/*z*: 399.2 (M^+^); anal. calcd for C_26_H_25_NO_3_: C, 78.17; H, 6.31; N, 3.51; O, 12.01. Found: C, 77.94; H, 6.26; N, 3.47.

#### *Trans*-3-bromo-5-(3,4-dimethoxyphenyl)-6-methyl-6,6*a*-dihydroisoindolo[2,1-*a*]quinolin-11(5*H*)-one (16)

Pale yellow solid, mp 224–225 °C. Yield 78%. IR (ATR): *ν*_max_ 3388, 2969, 1703, 1505, 1235, 751 cm^−1^. ^1^H NMR (400 MHz, CDCl_3_): *δ* (ppm) 8.38 (d, *J* = 8.8 Hz, 1H), 8.04–7.94 (m, 1H), 7.66–7.63 (m, 1H), 7.60 (td, *J* = 7.3, 1.4 Hz, 1H), 7.55 (td, *J* = 7.3, 1.4 Hz, 1H), 7.39 (ddd, *J* = 8.8, 2.4, 1.1 Hz, 1H), 6.92 (dd, *J* = 2.4, 1.1 Hz, 1H), 6.84 (d, *J* = 8.2 Hz, 1H), 6.75 (dd, *J* = 8.2, 2.0 Hz, 1H), 6.49 (d, *J* = 2.0 Hz, 1H), 4.52 (d, *J* = 10.7 Hz, 1H), 3.89 (s, 3H), 3.77 (s, 3H), 3.76 (d, *J* = 11.1 Hz, 1H), 1.94–1.84 (m, 1H), 1.25 (d, *J* = 6.5 Hz, 3H). ^13^C NMR (101 MHz, CDCl_3_): *δ* (ppm) 166.0, 149.6, 148.4, 143.1, 135.0, 134.9, 132.9, 132.8, 132.8, 131.93, 130.3, 129.0, 124.6, 124.4, 122.5, 122.0, 117.3, 111.4, 111.1, 64.1, 56.1, 56.0, 52.8, 41.4, 16.3. GC-MS (EI) *m*/*z*: 465 (M^+^); anal. calcd for C_25_H_22_BrNO_3_: C, 64.66; H, 4.78; N, 3.02; O, 10.34. Found: C, 64.61; H, 4.70; N, 2.58.

#### 9-Nitro-10*b*,15,15*a*,15*b*-tetrahydro-5*H*-indeno[2,1-*c*]isoindolo[2,1-*a*]quinolin-5-one (18)

Pale yellow solid, mp 260–261 °C. Yield 71%. IR (ATR): *ν*_max_ 2989, 1702, 1678, 1505, 1373, 1161, 849, 751 cm^−1^. ^1^H NMR (400 MHz, CDCl_3_): *δ* (ppm) 8.63 (d, *J* = 9.1 Hz, 1H), 8.45 (dd, *J* = 2.6, 1.1 Hz, 1H), 8.10 (ddd, *J* = 9.2, 2.6, 0.7 Hz, 1H), 8.02–7.97 (m, 1H), 7.71 (td, *J* = 7.3, 1.2 Hz, 1H), 7.64 (d, *J* = 7.6 Hz, 1H), 7.62–7.57 (m, 2H), 7.28–7.21 (m, 1H), 7.16 (td, *J* = 7.6, 1.2 Hz, 1H), 7.01 (d, *J* = 7.3 Hz, 1H), 5.22 (d, *J* = 3.6 Hz, 1H), 4.74 (d, *J* = 8.1 Hz, 1H), 3.81–3.73 (m, 1H), 2.41 (qd, *J* = 16.0, 9.4 Hz, 2H). ^13^C NMR (101 MHz, CDCl_3_): *δ* (ppm) 167.0, 144.2, 143.8, 142.9, 141.4, 141.2, 133.3, 132.2, 130.0, 129.3, 128.2, 127.6, 125.2, 125.0, 124.9, 124.9, 122.8, 122.2, 120.8, 59.3, 46.0, 42.5, 30.8. GC-MS (EI) *m*/*z*: 368 (M^+^); anal. calcd for C_23_H_16_N_2_O_3_: C, 74.99; H, 4.38; N, 7.60; O, 13.03. Found: C, 74.90; H, 4.31; N, 7.53.

#### 8,9,10-Trimethoxy-10*b*,15,15*a*,15*b*-tetrahydro-5*H*-indeno[2,1-*c*]isoindolo[2,1-*a*]quinolin-5-one (20)

White solid, mp 160 °C (dec.). Yield 60%. IR (ATR): *ν*_max_ 3388, 2970, 1704, 1505, 1235, 752 cm^−1^. ^1^H NMR (400 MHz, CDCl_3_): *δ* (ppm) 7.96–7.91 (m, 1H), 7.90–7.85 (m, 1H), 7.81 (s, 1H), 7.63 (td, *J* = 7.4, 1.2 Hz, 1H), 7.58–7.48 (m, 2H), 7.12–7.04 (m, 2H), 6.93 (d, *J* = 7.1 Hz, 1H), 4.99 (d, *J* = 3.8 Hz, 1H), 4.92 (d, *J* = 9.1 Hz, 1H), 4.06 (s, 3H), 3.88 (s, 3H), 3.85 (s, 3H), 3.80–3.72 (m, 1H), 2.44–2.34 (m, 2H). ^13^C NMR (101 MHz, CDCl_3_): *δ* (ppm) 166.3, 152.3, 152.1, 145.6, 143.1, 141.4, 139.2, 133.6, 132.3, 132.1, 128.8, 127.3, 127.0, 126.8, 124.4, 124.2, 122.1, 116.7, 99.80, 61.1, 60.7, 60.6, 56.1, 42.7, 41.9, 31.7. GC-MS (EI) *m*/*z*: 413.2 (M^+^); anal. calcd for C_26_H_23_NO_4_: C, 75.53; H, 5.61; N, 3.39; O, 15.48. Found: C, 75.48; H, 5.54; N, 3.32.

## Conclusions

In conclusion, we have studied the multicomponent Povarov reaction between anilines, several alkenes and 2-formylbenzoic acid in deep eutectic solvents. It was found that the eutectic mixture ChCl/ZnCl_2_ efficiently catalyses the reaction affording the target isoindoloquinolines with high diastereoselectivity and good yields employing *m*- and *p*-substituted anilines while the reaction with *o*-substituted analogues follow a different pathway yielding isoindolinones. The eutectic mixture also was recycled and reused in six consecutive cycles without observing a detrimental catalytic activity. This synthetic methodology offers a straight, green, and efficient alternative for the diastereoselective preparation of the target tetra- or hexacyclic fused heterocyclic molecules which could be useful for therapeutic and/or prophylactic drugs research.

## Conflicts of interest

There are no conflicts to declare.

## Supplementary Material

RA-013-D3RA05561B-s001
