# Supplementary material for: Diastereoselective multicomponent synthesis of dihydroisoindolo[2,1-a]quinolin-11-ones mediated by eutectic solvents
Source: RSC Adv. 2023 Sep 4;13(37):26189–95. doi: 10.1039/d3ra05561b (PMC10475973; doi:10.1039/d3ra05561b)
Supplement: RA-013-D3RA05561B-s001 [file RA-013-D3RA05561B-s001.pdf]

Electronic Supplementary Material (ESI) for RSC Advances.
This journal is © The Royal Society of Chemistry 2023

Diastereoselective multicomponent synthesis of dihydroisoindolo[2,1-*a*]quinolin-11-ones mediated by eutectic solvents

Carlos M. Sanabria-Sánchez,[a,b] Vladimir V. Kouznetsov*[a] and Cristian Ochoa-Puentes*[b]

a. Laboratorio de Química Orgánica y Biomolecular, Escuela de Química, Universidad Industrial de Santander, Cl. 9 # Cra 27, A.A. 680006, Bucaramanga, Santander, Colombia

b. Laboratorio de Síntesis Orgánica Sostenible, Departamento de Química, Universidad Nacional de Colombia-Sede Bogotá, Carrera 45 # 26-85, A.A 5997 Bogotá, Cundinamarca, Colombia

General procedure for the synthesis of dihydroisoindolo[2,1-*a*]quinolin-11-ones 1 - 20

2.9 g of ChCl/$ZnCl_2$ DES (1:2) (0.98 g; 7 mmol of choline chloride and 1.90 g; 14 mmol of zinc chloride) was heated to 80 °C to obtain a clear melt (2-3 minutes). To this melt a mixture of anilines (1 mmol), 2-formylbenzoic acid (1.1 mmol) and alkenes (1.2 mmol) was added, and the reaction mixture was stirred at 110 °C for 4.5 h. After completion of the reaction (monitored by TLC), the mixture was quenched by adding water while still hot, cooled to room temperature and the crude mixture was filtered. The resulting solid was purified by flash column chromatography on silica gel using petroleum ether-ethyl acetate mixtures as eluent to afford the isoindoloquinolines derivatives.

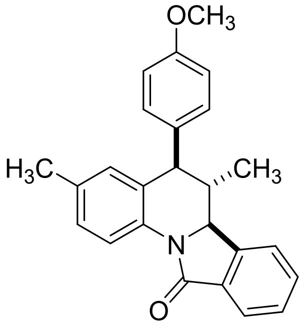

***Trans*-5-(4-methoxyphenyl)-3,6-dimethyl-6,6a-dihydroisoindolo[2,1-a]quinolin-11(5H)-one (1)**.[1] White solid, mp 183-184 °C. Yield 77%. IR (ATR): ν_{max} 2997, 1683, 1495, 1380, 1246, 832, 739 cm^{-1}; ^1H NMR (400 MHz, $CDCl_3$): δ (ppm) 8.36 (d, *J* = 8.3 Hz, 1H), 8.01 – 7.95 (m, 1H), 7.64 – 7.60 (m, 1H), 7.56 (td, *J* = 7.2, 1.5 Hz, 1H), 7.54 – 7.50 (m, 1H), 7.08 (m, 1H), 7.04 (d, *J* = 8.7 Hz, 2H), 6.85 (d, *J* = 8.7 Hz, 2H), 6.62 – 6.56 (m, 1H), 4.48 (d, *J* = 10.9 Hz, 1H), 3.80 (s, 3H), 3.76 (d, *J* = 11.0 Hz, 1H), 2.17 (s, 3H), 1.91 - 1.82 (m, 1H), 1.24 (d, *J* = 6.5 Hz, 3H); ^{13}C NMR (101 MHz, $CDCl_3$): δ (ppm) 165.6, 158.5, 143.2, 135.9, 133.6, 133.4, 133.2, 131.4, 130.6, 130.6, 130.5, 128.6, 127.8, 124.3, 124.2, 120.2, 114.0, 64.1, 55.3, 52.2, 42.0, 21.1, 16.2. GC–MS (EI) m/z: 369.2 (M^+); Anal. Calcd for $C_{25}H_{23}NO_2$: C, 81.27; H, 6.28; N, 3.79; O, 8.66. Found: C,81.23; H, 6.26; N, 3.71.

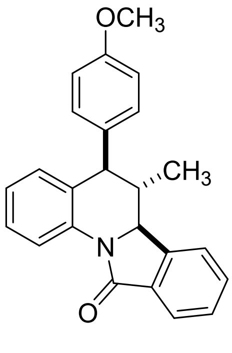

***Trans*-5-(4-methoxyphenyl)-6-methyl-6,6a-dihydroisoindolo[2,1-a]quinolin-11(5H)-one (2)**.[1] White solid, mp 208 - 210 °C. Yield 60%. IR (ATR): ν_{max} 3071, 2967, 1683, 1486, 1369, 1242, 754, 741 cm^{-1}; ^1H NMR (400 MHz, $CDCl_3$): δ (ppm) 8.48 (dd, *J* = 8.2, 1.3 Hz, 1H), 8.04 – 7.96 (m, 1H), 7.66 – 7.62 (m, 1H), 7.58 (td, *J* = 7.4, 1.6 Hz, 1H), 7.56 – 7.52 (m, 1H), 7.30 – 7.24 (m, 1H), 7.05 (d, *J* = 8.6 Hz, 2H), 6.97 (td, *J* = 7.5, 1.3 Hz, 1H), 6.85 (d, *J* = 8.6 Hz, 2H), 6.81 – 6.78 (m, 1H), 4.54 (d, *J* = 10.7 Hz, 1H), 3.80 (s, 3H), 3.80 (d, *J* = 8.2 Hz, 1H), 1.95 – 1.83 (m, 1H), 1.26 (d, *J* = 6.5 Hz, 3H). ^{13}C NMR (101 MHz, $CDCl_3$): δ (ppm) 165.9, 158.6, 143.3, 135.9, 135.9, 133.1, 131.6, 130.9, 130.6, 130.4, 128.8, 127.1, 124.5, 124.3, 124.2, 120.4, 114.1, 77.5, 77.2, 76.8, 64.2, 55.4, 52.4, 42.00, 16.3. GC–MS (EI) m/z: 355.2 (M^+); Anal. Calcd for $C_{24}H_{21}NO_2$: C, 81.10; H, 5.96; N, 3.94; O, 9.00. Found: C, 80.93; H, 5.90; N, 3.89.

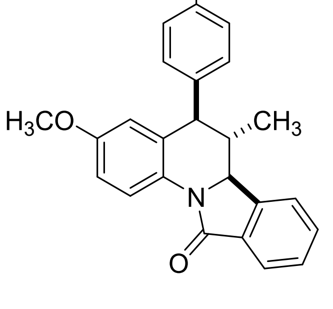

***Trans*-3-methoxy-5-(4-methoxyphenyl)-6-methyl-6,6a-dihydroisoindolo[2,1-a]quinolin-11(5H)-one (3)**. White solid. mp 271 – 272 °C. Yield 68%. IR (ATR): ν_{max} 3010, 2957, 1688, 1499, 1384, 1280, 832, 744 cm^{-1}. ^1H NMR (400 MHz, $CDCl_3$): δ (ppm) 8.40 (d, *J* = 9.0 Hz, 1H), 8.03 – 7.91 (m, 1H), 7.64 – 7.60 (m, 1H), 7.56 (dd, *J* = 7.2, 5.6 Hz, 1H), 7.53 (dd, *J* = 7.2, 5.6 Hz, 1H), 7.04 (d, *J* = 8.6 Hz, 2H), 6.84 (d, *J* = 8.6 Hz, 2H),

6.86 – 6.82 (m, 1H), 6.32 (dd, *J* = 2.9, 1.1 Hz, 1H), 4.49 (d, *J* = 10.7 Hz, 1H), 3.79 (s, 3H), 3.76 (d, *J* = 12.0 Hz, 1H), 3.65 (s, 3H), 1.95 – 1.80 (m, 1H), 1.24 (d, *J* = 6.5 Hz, 3H). ^{13}C NMR (101 MHz, $CDCl_3$): δ (ppm) 165.6, 158.7, 156.1, 143.2, 135.6, 133.3, 132.5, 131.4, 130.6, 129.5, 128.7, 124.3, 124.2, 121.5, 115.8, 114.2, 112.3, 64.2, 55.5, 55.4, 52.5, 42.0, 16.3. GC–MS (EI) m/z: 385.2 (M^+); Anal. Calcd for $C_{25}H_{23}NO_3$: C, 77.90; H, 6.01; N, 3.63; O, 12.45. Found: C, 77.82; H, 5.92; N, 3.54.

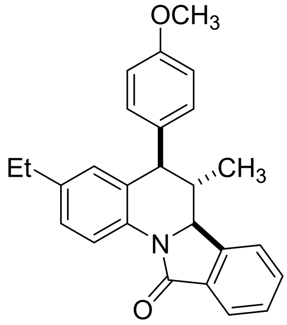

***Trans*-3-ethyl-5-(4-methoxyphenyl)-6-methyl-6,6a-dihydroisoindolo[2,1-a]quinolin-11(5H)-one (4)**.[1] White solid. mp 197 - 199 °C. Yield 51%. IR (ATR): ν_{max} 2960, 1683, 1498, 1381, 1246, 845, 740 cm^{-1}. ^{1}H NMR (400 MHz, $CDCl_3$): δ (ppm) 8.38 (d, *J* = 8.4 Hz, 1H), 7.99 (dd, *J* = 7.8, 1.1 Hz, 1H), 7.67 – 7.60 (m, 1H), 7.59 – 7.51 (m, 2H), 7.15 – 7.09 (m, 1H), 7.05 (d, *J* = 8.7 Hz, 2H), 6.85 (d, *J* = 8.7 Hz, 2H), 6.60 (d, *J* = 2.0 Hz, 1H), 4.51 (d, *J* = 10.7 Hz, 1H), 3.81 (d, *J* = 11.0 Hz, 1H), 3.81 (s, 3H), 2.46 (q, *J* = 7.6 Hz, 2H), 1.97 – 1.80 (m, 1H), 1.24 (d, *J* = 6.5 Hz, 3H), 1.09 (t, *J* = 7.6 Hz, 3H). ^{13}C NMR (101 MHz, $CDCl_3$): δ (ppm) 165.7, 158.6, 143.3, 140.2, 136.0, 133.7, 133.3, 131.5, 130.7, 130.6, 129.6, 128.7, 126.6, 124.4, 124.3, 120.3, 114.0, 64.2, 55.4, 52.4, 42.1, 28.5, 16.3, 15.9. GC–MS (EI) m/z: 383.2 (M^+); Anal. Calcd for $C_{26}H_{25}NO_2$: C, 81.43; H, 6.57; N, 3.65; O, 8.34. Found: C, 80.94; H, 6.50; N, 3.58.

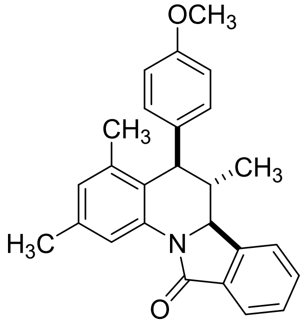

***Trans*-5-(4-methoxyphenyl)-2,4,6-trimethyl-6,6a-dihydroisoindolo[2,1-a]quinolin-11(5H)-one (5)**. White solid. mp 178 - 180 °C. Yield 69%. IR (ATR): ν_{max} 2971, 1685, 1507, 1379, 1242, 833, 742 cm^{-1}. ^{1}H NMR (400 MHz, $CDCl_3$): δ (ppm) 8.12 (d, *J* = 1.9 Hz, 1H), 7.99 – 7.95 (m, 1H), 7.60 – 7.57 (m, 1H), 7.52 (m, 2H), 6.96 (d, *J* = 8.7 Hz, 2H), 6.74 (d, *J* = 8.7 Hz, 2H), 6.71 (d, *J* = 1.9 Hz, 1H), 4.34 (d, *J* = 11.2 Hz, 1H), 3.74 (s, 3H), 3.72 (d, *J* = 9.5 Hz, 1H), 2.35 (s, 3H), 1.79 (s, 3H),1.74 – 1.68 (m, 1H), 1.38 (d, *J* = 6.6 Hz, 3H). ^{13}C NMR (101 MHz, $CDCl_3$): δ (ppm) 165.3, 158.0, 143.6, 138.0, 137.3, 136.9, 136.5, 133.4, 131.5, 129.9, 128.6, 128.5, 126.7, 124.4, 124.0, 118.9, 113.8, 63.5, 55.3, 50.3, 45.7, 21.3, 21.3, 16.6. GC–MS (EI) m/z: 383.2 (M^+); Anal. Calcd for $C_{26}H_{25}NO_2$: C, 81.43; H, 6.57; N, 3.65; O, 8.34. Found: C, 80.96; H, 6.51; N, 3.60.

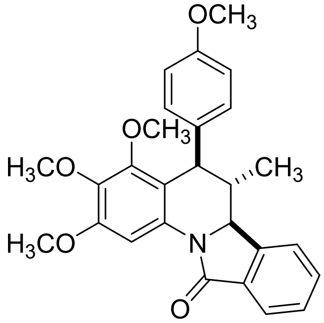

***Trans*-2,3,4-trimethoxy-5-(4-methoxyphenyl)-6-methyl-6,6a-dihydroisoindolo[2,1-a]quinolin-11(5H)-one (6).** White solid. mp 148 - 150 °C. Yield 87%. IR (ATR): ν_{max} 2971, 1682, 1457, 1396, 1232, 834, 755 cm^{-1}. ^{1}H NMR (400 MHz, $CDCl_3$): δ (ppm) 7.97 – 7.93 (m, 1H), 7.90 (s, 1H), 7.60 – 7.57 (m, 1H), 7.57 – 7.49 (m, 2H), 7.04 (d, *J* = 8.7 Hz, 2H), 6.78 (d, *J* = 8.7 Hz, 2H), 4.34 (d, *J* = 11.1 Hz, 1H), 3.94 (s, 3H), 3.79 (d, *J* = 10.0 Hz, 1H), 3.75 (s, 3H), 3.04 (s, 3H), 1.72 – 1.62 (m, 1H), 1.31 (d, *J* = 6.5 Hz, 3H).^{13}C NMR (101 MHz, $CDCl_3$): δ (ppm) 165.6, 158.0, 152.8, 151.9, 143.4, 139.6, 138.7, 133.2, 132.2, 131.6, 129.6, 128.7, 124.2, 124.1, 117.7, 113.6, 99.5, 63.8, 60.7, 59.5, 56.1, 55.4, 48.2, 44.5, 16.2. GC–MS (EI) m/z: 445.2 (M^+); Anal. Calcd for $C_{27}H_{27}NO_5$: C, 72.79; H, 6.11; N, 3.14; O, 17.96. Found: C, 72.71; H, 5.95; N, 2.98.

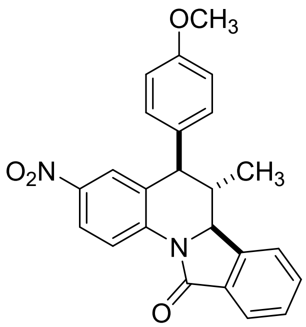

***Trans*-5-(4-methoxyphenyl)-6-methyl-3-nitro-6,6a-dihydroisoindolo[2,1-a]quinolin-11(5H)-one (7)**.[1] Yellow solid. mp 235 - 236 °C. Yield 81%. IR (ATR): ν_{max} 2992, 1780, 1506, 1328, 1245, 844, 747 cm^{-1}. ^{1}H NMR (400 MHz, $CDCl_3$): δ (ppm) 8.70 (d, *J* = 9.1 Hz, 1H), 8.12 (dd, *J* = 9.1, 2.6 Hz, 1H), 8.01 (dd, *J* = 7.5, 1.0 Hz, 1H), 7.70 – 7.62 (m, 3H), 7.58 (td, *J* = 7.2, 1.6 Hz, 1H), 7.04 (d, *J* = 8.7 Hz, 2H), 6.88 (d, *J* = 8.7 Hz, 2H), 4.61 (d, *J* = 10.7 Hz, 1H), 3.86 (d, *J* = 11.1 Hz, 1H), 3.81 (s, 3H), 1.98 – 1.88 (m, 1H), 1.29 (d, *J* = 6.5 Hz, 3H). ^{13}C NMR (101 MHz, $CDCl_3$): δ (ppm) 166.5, 159.1, 143.6, 143.0, 141.4, 134.0, 132.6, 132.1, 131.9, 130.4, 129.2, 126.1, 124.9, 124.5, 122.8, 120.4, 114.7, 64.1, 55.4, 52.3, 41.1, 16.1. GC–MS (EI) m/z: 400.2 (M^+); Anal. Calcd for $C_{24}H_{20}N_2O_4$: C, 71.99; H, 5.03; N, 7.00; O, 15.98. Found: C, 71.90; H, 4.93; N, 6.91.

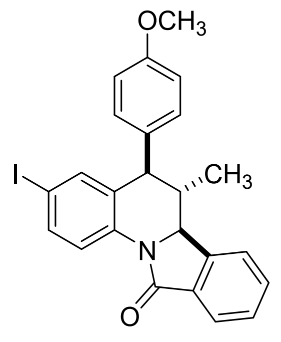

***Trans*-3-iodo-5-(4-methoxyphenyl)-6-methyl-6,6a-dihydroisoindolo[2,1-a]quinolin-11(5H)-one (8)**. White solid. mp 254 - 255 °C. Yield 55%. IR (ATR): ν_{max} 2992, 1688, 1476, 1378, 1246, 836, 739 cm^{-1}. ^{1}H NMR (400 MHz, $CDCl_3$): δ (ppm) 8.25 (d, *J* = 8.7 Hz, 1H), 8.02 – 7.95 (m, 1H), 7.65 – 7.61 (m, 1H), 7.61 – 7.51 (m, 3H), 7.09 (dd, *J* = 2.1, 1.1 Hz, 1H), 7.01 (d, *J* = 8.7 Hz, 2H), 6.86 (d, *J* = 8.7 Hz, 2H), 4.50 (d, *J* = 10.7 Hz, 1H), 3.81 (s, 3H), 3.75 (d, *J* = 11.1 Hz, 1H), 1.92 – 1.79 (m, 1H), 1.23 (d, *J* = 6.5 Hz, 3H). ^{13}C NMR (101 MHz, $CDCl_3$): δ (ppm) 165.9, 158.8, 143.2, 139.0, 136.1, 135.7, 134.9, 133.3, 132.8, 131.9, 130.5, 128.9, 124.6, 124.4, 122.3, 114.4, 88.1, 64.0, 55.4, 52.0, 41.6, 16.2. GC–MS (EI) m/z: 355.1 (M^+-I); Anal. Calcd for $C_{24}H_{20}INO_2$: C, 59.89; H, 4.19; N, 2.91; O, 6.65. Found: C, 59.84; H, 4.12; N, 2.86.

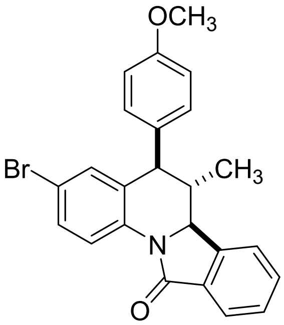

***Trans*-3-bromo-5-(4-methoxyphenyl)-6-methyl-6,6a-dihydroisoindolo[2,1-a]quinolin-11(5H)-one (9)**. White solid. mp 231 - 232 °C. Yield 68%. IR (ATR): ν_{max} 2961, 1685, 1513, 1381, 1246, 835, 740 cm^{-1}. ^{1}H NMR (400 MHz, $CDCl_3$): δ (ppm) 8.39 (d, *J* = 8.8 Hz, 1H), 8.00 – 7.97 (m, 1H), 7.65 – 7.62 (m, 1H), 7.59 (td, *J* = 7.3, 1.5 Hz, 1H), 7.54 (td, *J* = 7.3, 1.5 Hz, 1H), 7.37 (ddd, *J* = 8.8, 2.4, 1.1 Hz, 1H), 7.02 (d, *J* = 8.7 Hz, 2H), 6.90 (dd, *J* = 2.4, 1.1 Hz, 1H), 6.86 (d, *J* = 8.7 Hz, 2H), 4.51 (d, *J* = 10.7 Hz, 1H), 3.81 (s, 3H), 3.77 (d, *J* = 11.1 Hz, 1H), 1.93 – 1.80 (m, 1H), 1.24 (d, *J* = 6.5 Hz, 3H). ^{13}C NMR (101 MHz, $CDCl_3$): δ (ppm) 165.9, 158.8, 143.1, 135.0, 134.8, 133.1, 133.0, 132.8, 131.9, 130.5, 130.2, 128.9, 124.6, 124.4, 122.0, 117.2, 114.4, 64.1, 55.4, 52.2, 41.6, 16.2. GC–MS (EI) m/z: 433 (M^+); Anal. Calcd for $C_{24}H_{20}BrNO_2$: C, 66.37; H, 4.64; N, 3.22; O, 7.37 Found: C, 65.94; H, 4.60; N, 3.18.

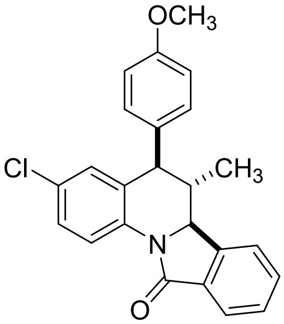

***Trans*-3-chloro-5-(4-methoxyphenyl)-6-methyl-6,6a-dihydroisoindolo[2,1-a]quinolin-11(5H)-one (10)**. White solid. mp 210 - 211 °C. Yield 64%. IR (ATR): ν_{max} 2980, 1686, 1513, 1378, 1263, 835, 739 cm^{-1}. ^{1}H NMR (400 MHz, $CDCl_3$): δ (ppm) 8.44 (d, *J* = 8.8 Hz, 1H), 8.01 – 7.96 (m, 1H), 7.65 – 7.61 (m, 1H), 7.59 (td, *J* = 7.3, 1.5 Hz, 1H), 7.54 (td, *J* = 7.3, 1.5 Hz, 1H), 7.23 (ddd, *J* = 8.8, 2.5, 1.1 Hz, 1H), 7.02 (d, *J* = 8.6 Hz, 2H), 6.86 (d, *J* = 8.7 Hz, 2H), 6.75 (dd, *J* = 2.5, 1.1 Hz, 1H), 4.51 (d, *J* = 10.7 Hz, 1H), 3.81 (s, 3H), 3.76 (d, *J* = 11.0 Hz, 1H), 1.92 - 1.82 (m, 1H), 1.24 (d, *J* = 6.5 Hz, 3H). ^{13}C NMR (101 MHz, $CDCl_3$): δ (ppm) 165.9, 158.8, 143.1, 134.9, 134.5, 132.8, 132.7, 131.8, 130.5, 130.1, 129.3, 128.9, 127.2, 124.5, 124.3, 121.7, 114.4, 64.1, 55.4, 52.2, 41.6, 16.2. GC–MS (EI) m/z: 389.1 (M^+); Anal. Calcd for $C_{24}H_{20}ClNO_2$: C, 73.94; H, 5.17; N, 3.59; O, 8.21. Found: C, 73.88; H, 5.11; N, 3.53.

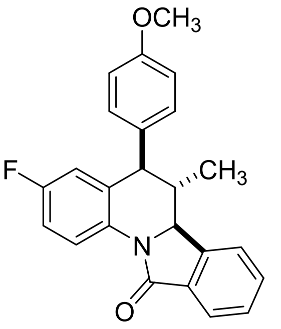

***Trans*-3-fluoro-5-(4-methoxyphenyl)-6-methyl-6,6a-dihydroisoindolo[2,1-a]quinolin-11(5H)-one (11)**. White solid. mp 219 - 220 °C. Yield 57%. IR (ATR): ν_{max} 2962, 1689, 1490, 1380, 1245, 828, 743 cm^{-1}. ^{1}H NMR (400 MHz, $CDCl_3$): δ (ppm) 8.45 (dd, *J* = 8.9, 5.4 Hz, 1H), 8.02 – 7.95 (m, 1H), 7.63 (d, *J* = 7.4 Hz, 1H), 7.59 (dd, *J* = 7.3, 1.4 Hz, 1H), 7.56 – 7.50 (m, 1H), 7.03 (d, *J* = 8.6 Hz, 2H), 6.97 (td, *J* = 8.9, 2.9 Hz, 1H), 6.86 (d, *J* = 8.6 Hz, 2H), 6.51 – 6.45 (m, 1H), 4.52 (d, *J* = 10.7 Hz, 1H), 3.80 (s, 3H), 1.93 - 1.83 (m, 1H), 1.25 (d, *J* = 6.5 Hz, 3H). ^{13}C NMR (101 MHz, $CDCl_3$): δ (ppm) 165.82, 160.45, 159.24 (d, *J* = 243.3 Hz), 158.84, 158.03, 143.11, 135.01, 133.10 (d, *J* = 6.9 Hz), 132.94, 132.02 (d, *J* = 2.5 Hz), 131.71, 130.51, 128.86, 124.49, 124.30, 121.95 (d, *J* = 8.0 Hz), 116.59 (d, *J* = 23.1 Hz), 114.32, 114.14 (d, *J* = 22.4 Hz), 77.48, 77.16, 76.84, 64.16, 55.39, 52.43, 41.70, 16.23. ^{19}F NMR (376 MHz, $CDCl_3$): δ (ppm) -117.6. GC–MS (EI) m/z: 373.2 (M^+); Anal. Calcd for $C_{24}H_{20}FNO_2$: C, 77.19; H, 5.40; N, 3.75; O, 8.57. Found: C, 77.12; H, 5.31; N, 3.69.

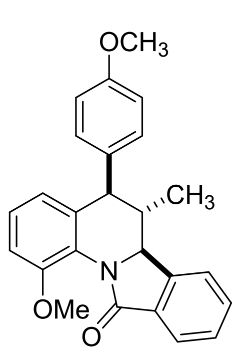

***Trans*-1-methoxy-5-(4-methoxyphenyl)-6-methyl-6,6a-dihydroisoindolo[2,1-a]quinolin-11(5H)-one (12)**. Oil. Yield 32 %. IR (ATR): ν_{max} XXX cm^{-1}. ^{1}H NMR (400 MHz, $CDCl_3$): δ (ppm) 7.99 – 7.96 (m, 1H), 7.57 – 7.51 (m, 1H), 7.53 – 7.47 (m, 2H), 7.05 (d, *J* = 8.8 Hz, 2H), 7.01 (d, *J* = 8.0 Hz, 1H), 6.88 (d, *J* = 8.2 Hz, 1H), 6.80 (d, *J* = 8.6 Hz, 2H), 6.44 (d, *J* = 7.9 Hz, 1H), 4.43 (d, *J* = 10.4 Hz, 1H), 3.96 (s, 3H), 3.78 (d, *J* = 11.0 Hz, 1H), 3.76 (s, 3H), 1.77 – 1.63 (m, 1H), 1.18 (d, *J* =

6.5 Hz, 3H). ^{13}C NMR (101 MHz, $CDCl_3$): δ (ppm) 164.5, 158.5, 153.0, 145.2, 136.1, 134.8, 132.8, 131.0, 130.3, 128.5, 126.1, 125.0, 124.6, 123.9, 121.9, 113.9, 110.6, 64.2, 56.2, 55.3, 51.9, 44.8, 16.6. GC–MS (EI) m/z: 385.2 (M^+); Anal. Calcd for $C_{25}H_{23}NO_3$: C, 77.90; H, 6.01; N, 3.63; O, 12.45. Found: C, 77.83; H, 5.94; N, 3.59.

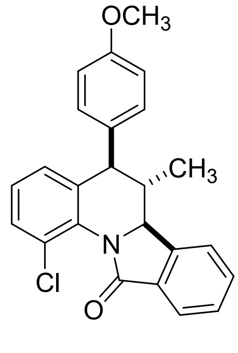

***Trans*-1-chloro-5-(4-methoxyphenyl)-6-methyl-6,6a-dihydroisoindolo[2,1-a]quinolin-11(5H)-one (13)**. White solid. mp 130 – 131 °C. Yield 30%. IR (ATR): ν_{max} 3388, 2955, 1672, 1509, 1243, 1036, 752 cm^{-1}. ^{1}H NMR (400 MHz, DMSO): δ (ppm) 7.75 – 7.72 (m, 2H), 7.57 (t, *J* = 7.4 Hz, 1H), 7.44 – 7.35 (m, 1H), 7.14 (d, *J* = 8.6 Hz, 2H), 7.09 (d, *J* = 7.7 Hz, 1H), 6.89 (d, *J* = 8.6 Hz, 2H), 6.45 (t, *J* = 7.8 Hz, 1H), 6.33 (d, *J* = 7.7 Hz, 1H), 5.03 (d, *J* = 10.0 Hz, 1H), 3.79 (d, *J* = 10.9 Hz, 1H), 3.74 (s, 3H), 2.29 (sa, 1H), 0.46 (d, *J* = 6.5 Hz, 3H). ^{13}C NMR (101 MHz, DMSO): δ (ppm) 169.3, 157.8, 141.2, 135.5, 132.9, 130.1, 129.0, 128.2, 127.4, 127.4, 126.6, 117.1, 116.7, 113.9, 55.0, 50.4, 15.8. GC–MS (EI) m/z: 389.2 (M^+); Anal. Calcd for $C_{24}H_{20}ClNO_2$: C, 73.94; H, 5.17; N, 3.59; O, 8.21. Found: C, 73.89; H, 5.10; N, 3.51.

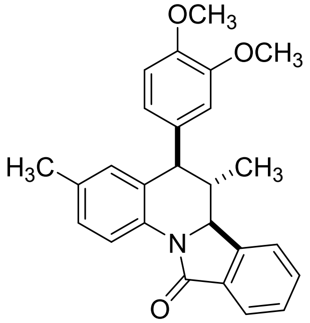

***Trans*-5-(3,4-dimethoxyphenyl)-3,6-dimethyl-6,6a-dihydroisoindolo[2,1-a]quinolin-11(5H)-one (14)**. White solid. mp 218 - 219 °C. Yield 78%. IR (ATR): ν_{max} 3077, 2989, 1678, 1517, 1383, 1233, 880, 747 cm^{-1}. ^{1}H NMR (400 MHz, $CDCl_3$): δ (ppm) 8.35 (d, *J* = 8.3 Hz, 1H), 7.99 (dd, *J* = 7.6, 1.2 Hz, 1H), 7.66 – 7.60 (m, 1H), 7.60 – 7.55 (m, 1H), 7.55 – 7.51 (m, 1H), 7.09 (dt, *J* = 8.3, 1.0 Hz, 1H), 6.83 (d, *J* = 8.2 Hz, 1H), 6.77 (dd, *J* = 8.2, 2.0 Hz, 1H), 6.65 – 6.57 (m, 1H), 6.52 (d, *J* = 2.0 Hz, 1H), 4.50 (d, *J* = 10.7 Hz, 1H), 3.89 (s, 3H), 3.76 (s, 3H), 2.18 (s, 3H), 1.95 – 1.82 (m, 1H), 1.25 (d, *J* = 6.5 Hz, 3H). ^{13}C NMR (101 MHz, $CDCl_3$): δ (ppm) 165. 8, 149.4, 148.1, 143.3, 136.2, 133.8, 133.4, 133.2, 131.5, 130.56, 130.4, 128.8, 127.9, 124.4, 124.3, 122.5, 120.2, 111.7, 110. 9, 64.2, 56.1, 56.0, 52.9, 41.8, 21.1, 16.4. GC–MS (EI) m/z: 399.2 (M^+); Anal. Calcd for $C_{26}H_{25}NO_3$: C, 78.17; H, 6.31; N, 3.51; O, 12.01. Found: C, 77.94; H, 6.26; N, 3.47.

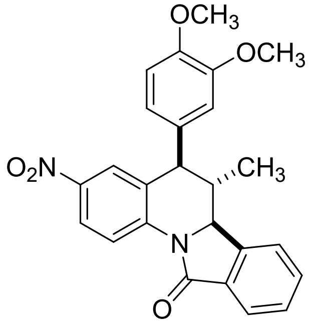

***Trans*-5-(3,4-dimethoxyphenyl)-6-methyl-3-nitro-6,6a-dihydroisoindolo[2,1-a]quinolin-11(5H)-one (15)**. White solid. mp 235 - 236 °C. Yield 66%. IR (ATR): ν_{max} 3389, 2971, 1705, 1504, 1313, 1236, 843, 753 cm^{-1}. ^{1}H NMR (400 MHz, $CDCl_3$): δ (ppm) 8.70 (d, *J* = 9.2 Hz, 1H), 8.13 (ddd, *J* = 9.2, 2.6, 1.2 Hz, 1H), 8.02 (d, *J* = 7.6 Hz, 1H), 7.72 (dd, *J* = 2.6, 1.2 Hz, 1H), 7.69 – 7.63 (m, 2H), 7.59 (td, *J* = 7.2, 1.5 Hz, 1H), 6.86 (d, *J* = 8.2 Hz, 1H), 6.77 (dd, *J* = 8.2, 2.0 Hz, 1H), 6.49 (d, *J* = 2.0 Hz, 1H), 4.62 (d, *J* = 10.7 Hz, 1H), 3.90 (s, 3H), 3.85 (d, *J* = 11.2 Hz, 1H), 3.77 (s, 3H), 2.00 – 1.90 (m, 1H), 1.30 (d, *J* = 6.5 Hz, 3H). ^{13}C NMR (101 MHz, $CDCl_3$): δ (ppm) 166.6, 149.8, 148. 143.7, 143.0, 141.4, 134.3, 132. 7, 132.1, 131.7, 129.3, 126.1, 125.0, 124.5, 122.9, 122.4, 120.5, 111.5, 111.4, 64.2, 56.2, 56.0, 52.8, 40.9, 16.2. GC–MS (EI) m/z: 430.2 (M^+); Anal. Calcd for $C_{25}H_{22}N_2O_5$: C, 69.76; H, 5.15; N, 6.51; O, 18.58. Found: C, 69.72; H, 5.07; N, 6.45.

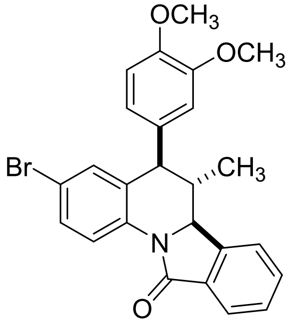

***Trans*-3-bromo-5-(3,4-dimethoxyphenyl)-6-methyl-6,6a-dihydroisoindolo[2,1-a]quinolin-11(5H)-one (16)**. Pale yellow solid. mp 224-225 °C. Yield 78%. IR (ATR): ν_{max} 3388, 2969, 1703, 1505, 1235, 751 cm^{-1}. ^{1}H NMR (400 MHz, $CDCl_3$): δ (ppm) 8.38 (d, *J* = 8.8 Hz, 1H), 8.04 – 7.94 (m, 1H), 7.66 – 7.63 (m, 1H), 7.60 (td, *J* = 7.3, 1.4 Hz, 1H), 7.55 (td, *J* = 7.3, 1.4 Hz, 1H), 7.39 (ddd, *J* = 8.8, 2.4, 1.1 Hz, 1H), 6.92 (dd, *J* = 2.4, 1.1 Hz, 1H), 6.84 (d, *J* = 8.2 Hz, 1H), 6.75 (dd, *J* = 8.2, 2.0 Hz, 1H), 6.49 (d, *J* = 2.0 Hz, 1H), 4.52 (d, *J* = 10.7 Hz, 1H), 3.89 (s, 3H), 3.77 (s, 3H), 3.76 (d, *J* = 11.1 Hz, 1H), 1.94 - 1.84 (m, 1H), 1.25 (d, *J* = 6.5 Hz, 3H). ^{13}C NMR (101 MHz, $CDCl_3$): δ (ppm) 166.0, 149.6, 148.4, 143.1, 135.0, 134.9, 132.9, 132.8, 132.8, 131.93, 130.3, 129.0, 124.6, 124.4, 122.5, 122.0, 117.3, 111.4, 111.1,

64.1, 56.1, 56.0, 52.8, 41.4, 16.3. GC–MS (EI) m/z: 465 (M^+); Anal. Calcd for $C_{25}H_{22}BrNO_3$: C, 64.66; H, 4.78; N, 3.02; O, 10.34. Found: C, 64.61; H, 4.70; N, 2.58.

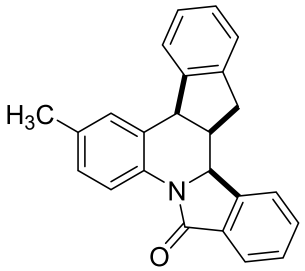

***Trans*-9-methyl-10b,15,15a,15b-tetrahydro-5H-indeno[2,1-c]isoindolo[2,1-a]quinolin-5-one (17)**. White solid. mp 106 - 107 °C. Yield 75%. IR (ATR): ν_{max} 2973, 1682, 1502, 1387, 1232, 816, 753 cm^{-1}. ^{1}H NMR (400 MHz, $CDCl_3$): δ (ppm) 8.29 (d, *J* = 8.3 Hz, 1H), 7.97 (dd, *J* = 7.4, 0.9 Hz, 1H), 7.65 – 7.62 (m, 1H), 7.59 (d, *J* = 7.6 Hz, 1H), 7.59 – 7.50 (m, 2H), 7.36 (d, *J* = 1.9 Hz, 1H), 7.22 – 7.17 (m, 1H), 7.12 (td, *J* = 7.6, 1.2 Hz, 1H), 7.09 – 7.04 (m, 1H), 6.99 (d, *J* = 7.6 Hz, 1H), 5.11 (d, *J* = 3.6 Hz, 1H), 4.62 (d, *J* = 8.1 Hz, 1H), 3.70 - 3.62 (m, 1H), 2.37 (dd, *J* = 9.8, 5.8 Hz, 2H), 2.32 (s, 3H). ^{13}C NMR (101 MHz, $CDCl_3$): δ (ppm) 166.3, 145.5, 143.2, 141.8, 134.2, 133.5, 133.0, 132.2, 129.3, 128.7, 128.7, 127.9, 127.6, 126.9, 125.0, 125.0, 124.4, 122.00, 120.6, 59.4, 45.9, 43.0, 30.8, 21.3. GC–MS (EI) m/z: 337.2 (M^+); Anal. Calcd for C24H19NO: C, 85.43; H, 5.68; N, 4.15; O, 4.74. Found: C, 85.38; H, 5.60; N, 3.98.

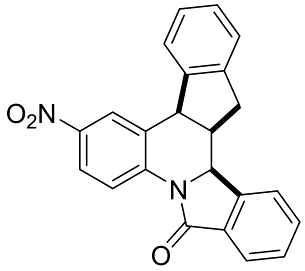

***Trans*-9-nitro-10b,15,15a,15b-tetrahydro-5H-indeno[2,1-c]isoindolo[2,1-a]quinolin-5-one (18)**. Pale yellow solid. mp 260 - 261 °C. Yield 71%. IR (ATR): ν_{max} 2989, 1702, 1678, 1505, 1373, 1161, 849, 751 cm^{-1}. ^{1}H NMR (400 MHz, $CDCl_3$): δ (ppm) 8.63 (d, *J* = 9.1 Hz, 1H), 8.45 (dd, *J* = 2.6, 1.1 Hz, 1H), 8.10 (ddd, *J* = 9.2, 2.6, 0.7 Hz, 1H), 8.02 – 7.97 (m, 1H), 7.71 (td, *J* = 7.3, 1.2 Hz, 1H), 7.64 (d, *J* = 7.6 Hz, 1H), 7.62 – 7.57 (m, 2H), 7.28 – 7.21 (m, 1H), 7.16 (td, *J* = 7.6, 1.2 Hz, 1H), 7.01 (d, *J* = 7.3 Hz, 1H), 5.22 (d, *J* = 3.6 Hz, 1H), 4.74 (d, *J* = 8.1 Hz, 1H), 3.81 – 3.73 (m, 1H), 2.41 (qd, *J* = 16.0, 9.4 Hz, 2H). ^{13}C NMR (101 MHz, $CDCl_3$): δ (ppm) 167.0, 144.2, 143.8, 142.9, 141.4, 141.2, 133.3, 132.2, 130.0, 129.3, 128.2, 127.6, 125.2, 125.0, 124.9, 124.9, 122.8, 122.2, 120.8, 59.3, 46.0, 42.5, 30.8. GC–MS (EI) m/z: 368 (M^+); Anal. Calcd for $C_{23}H_{16}N_2O_3$: C, 74.99; H, 4.38; N, 7.60; O,13.03. Found: C, 74.90; H, 4.31; N, 7.53.

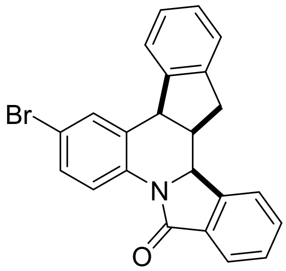

***Trans*-9-bromo-10b,15,15a,15b-tetrahydro-5H-indeno[2,1-c]isoindolo[2,1-a]quinolin-5-one (19)**. Pale yellow solid. mp 109-110 °C. Yield 62%. IR (ATR): ν_{max} 3388, 2971, 1704, 1505, 1235, 843, 751 cm^{-1}. ^{1}H NMR (400 MHz, $CDCl_3$): δ (ppm) 8.31 (d, *J* = 8.8 Hz, 1H), 7.96 (d, *J* = 7.5 Hz, 1H), 7.68 – 7.61 (m, 2H), 7.58 – 7.50 (m, 3H), 7.34 (ddd, *J* = 8.8, 2.3, 0.8 Hz, 1H), 7.24 – 7.18 (m, 1H), 7.13 (td, *J* = 7.5, 1.2 Hz, 1H), 6.99 (d, *J* = 7.4 Hz, 1H), 5.08 (d, *J* = 3.6 Hz, 1H), 4.60 (d, *J* = 8.1 Hz, 1H), 3.70 – 3.59 (m, 1H), 2.35 (d, *J* = 9.2 Hz, 2H). ^{13}C NMR (101 MHz, $CDCl_3$): δ (ppm) 166.4, 144.6, 143.0, 141.6, 134.6, 132.9, 132.5, 131.8, 131.1, 130.1, 128.9, 127.9, 127.2, 125.1, 124.8, 124.5, 122.3, 122.0, 117.5, 59.2, 45.8, 42.8, 30.7. GC–MS (EI) m/z: 401 (M^+); Anal. Calcd for $C_{23}H_{16}BrNO$: C, 68.67; H, 4.01; N, 3.48; O, 3.98. Found: C, 68,69; H, 3.97; N, 3.41.

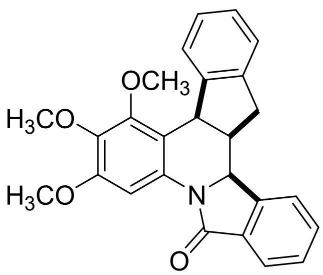

***Trans*-8,9,10-trimethoxy-10b,15,15a,15b-tetrahydro-5H-indeno[2,1-c]isoindolo[2,1-a]quinolin-5-one (20)**. White solid. mp 160 °C (Dec.). Yield 60%. IR (ATR): ν_{max} 3388, 2970, 1704, 1505, 1235, 752 cm^{-1}. ^{1}H NMR (400 MHz, $CDCl_3$): δ (ppm) 7.96 – 7.91 (m, 1H), 7.90 – 7.85 (m, 1H), 7.81 (s, 1H), 7.63 (td, *J* = 7.4, 1.2 Hz, 1H), 7.58 – 7.48 (m, 2H), 7.12 -7.04 (m, 2H), 6.93 (d, *J* = 7.1 Hz, 1H), 4.99 (d, *J* = 3.8 Hz, 1H), 4.92 (d, *J* = 9.1 Hz, 1H), 4.06 (s, 3H), 3.88 (s, 3H), 3.85 (s, 3H), 3.80 – 3.72 (m, 1H), 2.44 – 2.34 (m, 2H). ^{13}C NMR (101 MHz, $CDCl_3$): δ (ppm) 166.3, 152.3, 152.1, 145.6, 143.1, 141.4, 139.2, 133.6, 132.3, 132.1, 128.8, 127.3, 127.0, 126.8, 124.4, 124.2, 122.1, 116.7, 99.80, 61.1, 60.7, 60.6, 56.1, 42.7, 41.9, 31.7. GC–MS (EI) m/z: 413.2 (M^+); Anal. Calcd for $C_{26}H_{23}NO_4$: C, 75.53; H, 5.61; N, 3.39; O, 15.48. Found: C, 75.48; H, 5.54; N, 3.32.

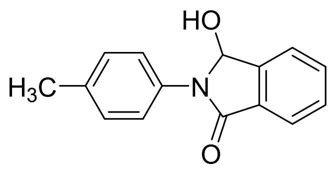

3-hydroxy-2-(p-tolyl)isoindolin-1-one (21).[2] Brown oil. Yield 66%. ^{1}H NMR (400 MHz, $CDCl_3$): δ (ppm) 7.91 (d, *J* = 7.6 Hz, 1H), 7.77 – 7.70 (m, 1H), 7.66 – 7.61 (m, 2H), 7.09 (d, *J* = 8.4 Hz, 2H), 6.87 (d, *J* = 8.4 Hz, 2H), 6.80 (d, *J* = 10.7 Hz, 1H), 4.62 (d, *J* = 11.1 Hz, 1H), 2.29 (s, 3H). ^{13}C NMR (101 MHz, $CDCl_3$): δ (ppm) 169.4, 145.5, 141.3, 134.4, 130.8, 130.6, 130.1, 129.8, 128.2, 125.7, 123.5, 115.6, 115.4, 88.2, 20.7.

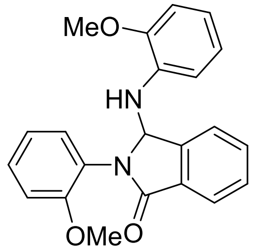

2-(2-methoxyphenyl)-3-((2-methoxyphenyl)amino)isoindolin-1-one (22). White powder. Yield 13%. ^{1}H NMR (400 MHz, $CDCl_3$): δ (ppm) 7.95 (d, *J* = 7.6 Hz, 1H), 7.75 (td, *J* = 7.5, 1.1 Hz, 1H), 7.69 – 7.61 (m, 2H), 7.15 (dd, *J* = 7.8, 1.6 Hz, 1H), 6.96 (td, *J* = 7.6, 1.7 Hz, 1H), 6.93 – 6.89 (m, 1H), 6.89 – 6.84 (m, 2H), 6.82 – 6.77 (m, 2H), 6.75 – 6.71 (m, 2H), 5.24 (d, *J* = 10.8 Hz, 1H), 3.85 (s, 3H), 3.83 (s, 3H). ^{13}C NMR (101 MHz, $CDCl_3$): δ (ppm) 169.3, 147.4, 147.4, 145.5, 136.2, 134.3, 133.5, 130.7, 128.1, 125.6, 123.4, 121.3, 121.1, 120.4, 118.5, 115.0, 112.7, 110.5, 110.3, 86.9, 55.5, 55.4.

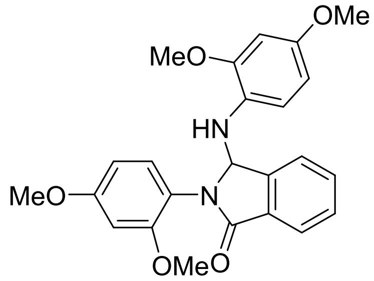

2-(2,4-dimethoxyphenyl)-3-((2,4-dimethoxyphenyl)amino)isoindolin-1-one (23). White powder. Yield 43%. ^{1}H NMR (400 MHz, $CDCl_3$): δ (ppm) 7.90 (d, *J* = 7.6 Hz, 1H), 7.71 (td, *J* = 7.5, 1.1 Hz, 1H), 7.67 – 7.64 (m, 1H), 7.62 – 7.57 (m, 1H), 7.02 (d, *J* = 8.4 Hz, 1H), 6.80 (s, 1H), 6.62 (d, *J* = 8.4 Hz, 1H), 6.48 - 6.43 (m, 3H), 6.33 (dd, *J* = 8.4, 2.7 Hz, 1H), 4.94 (s, 1H), 3.80 (s, 3H), 3.77 (s, 3H), 3.77 (s, 3H), 3.73 (s, 3H). ^{13}C NMR (101 MHz, $CDCl_3$): δ (ppm) 169.4, 158.5, 154.5, 153.2, 148.7, 148.4, 145.6, 134.2, 130.6, 129.7, 128.2, 127.3, 125.5, 123.5, 115.3, 113.6, 104.2, 104.0, 99.4, 99.4, 55.8, 55.7, 55.5, 55.5.

NMR SPECTRA

***Trans*-5-(4-methoxyphenyl)-3,6-dimethyl-6,6a-dihydroisoindolo[2,1-a]quinolin-11(5H)-one (1)**

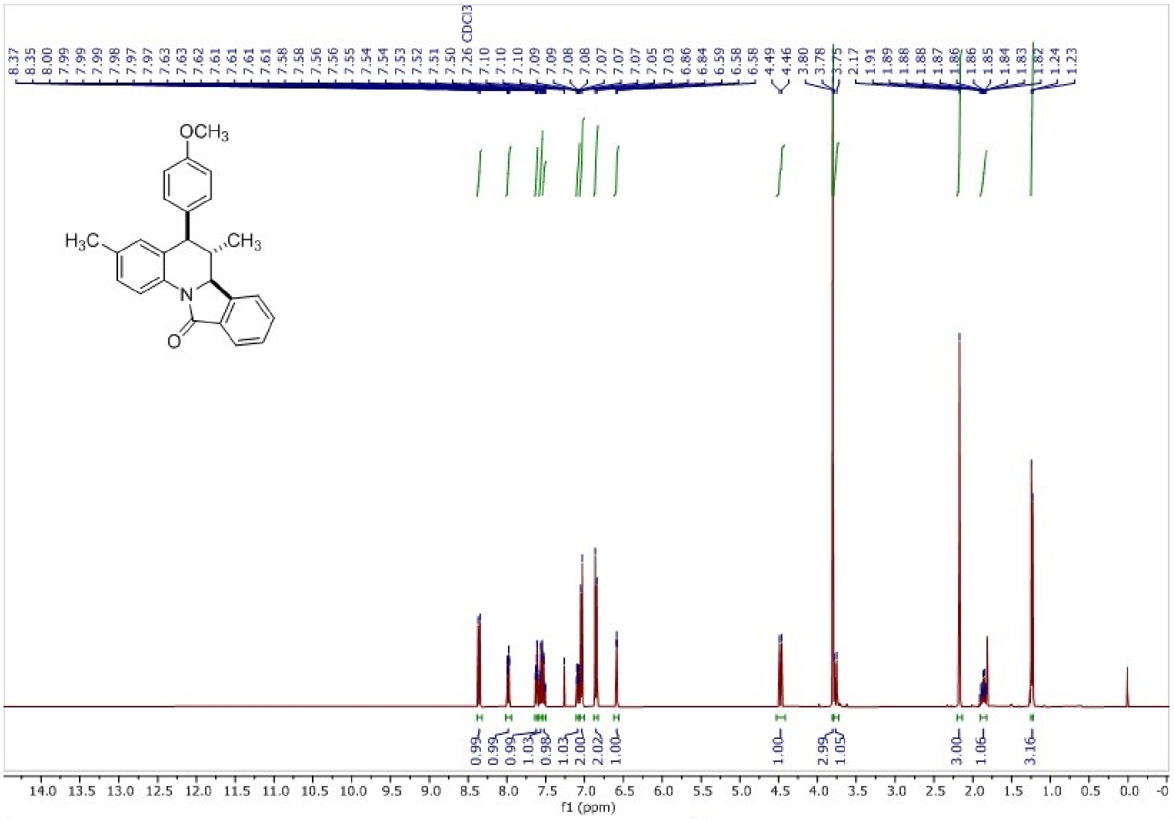

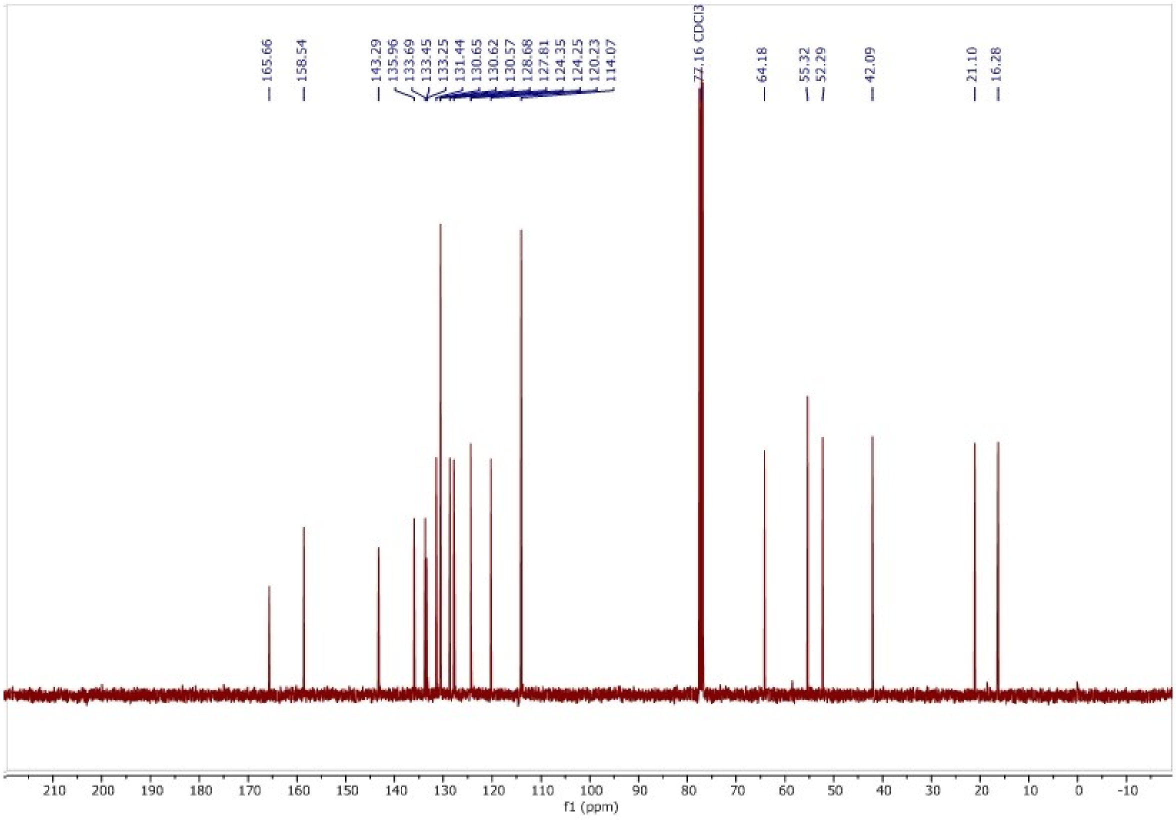

***Trans*-5-(4-methoxyphenyl)-6-methyl-6,6a-dihydroisoindolo[2,1-a]quinolin-11(5H)-one (2)**

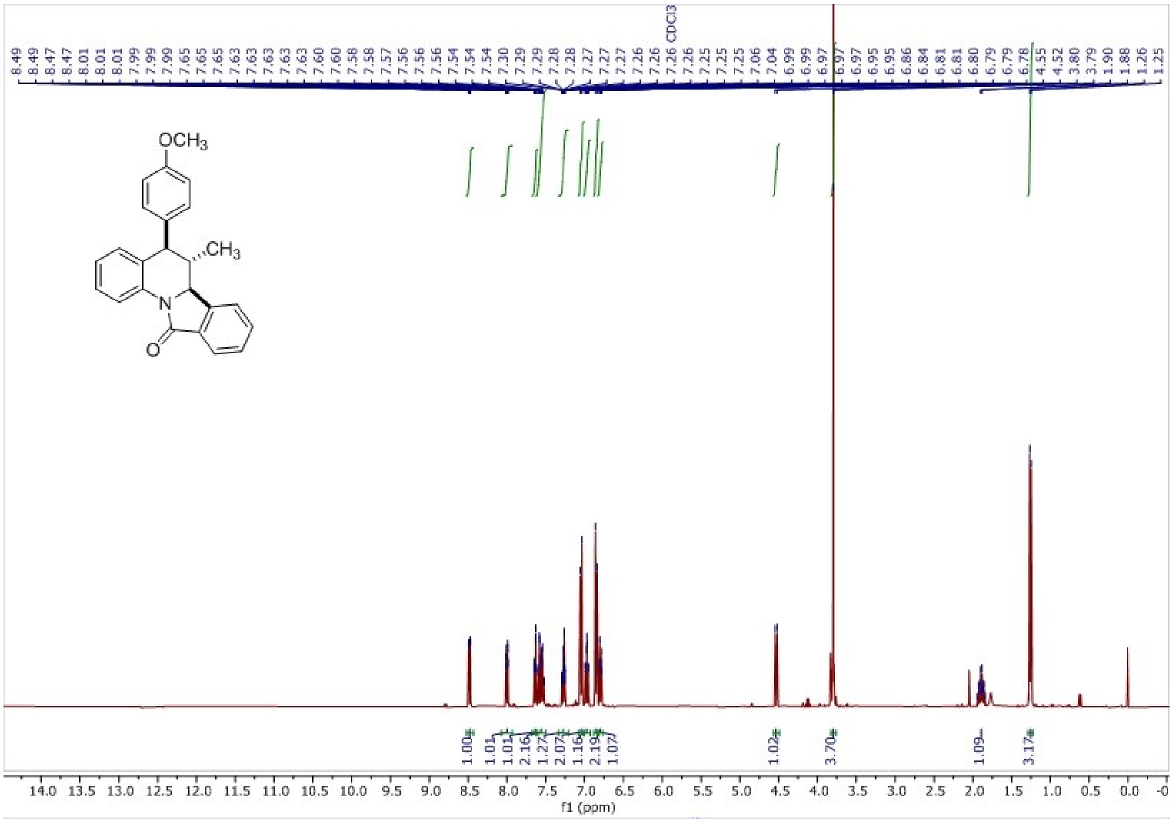

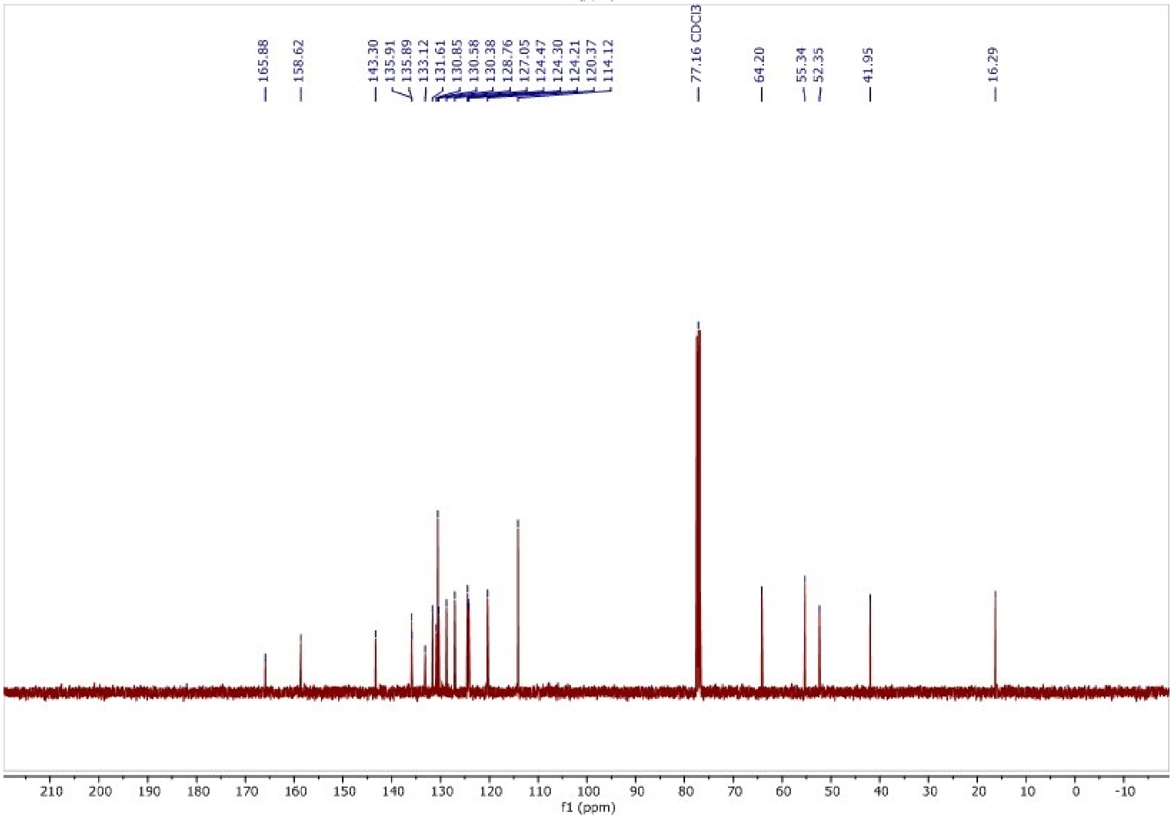

***Trans*-3-methoxy-5-(4-methoxyphenyl)-6-methyl-6,6a-dihydroisoindolo[2,1-a]quinolin-11(5H)-one (3)**

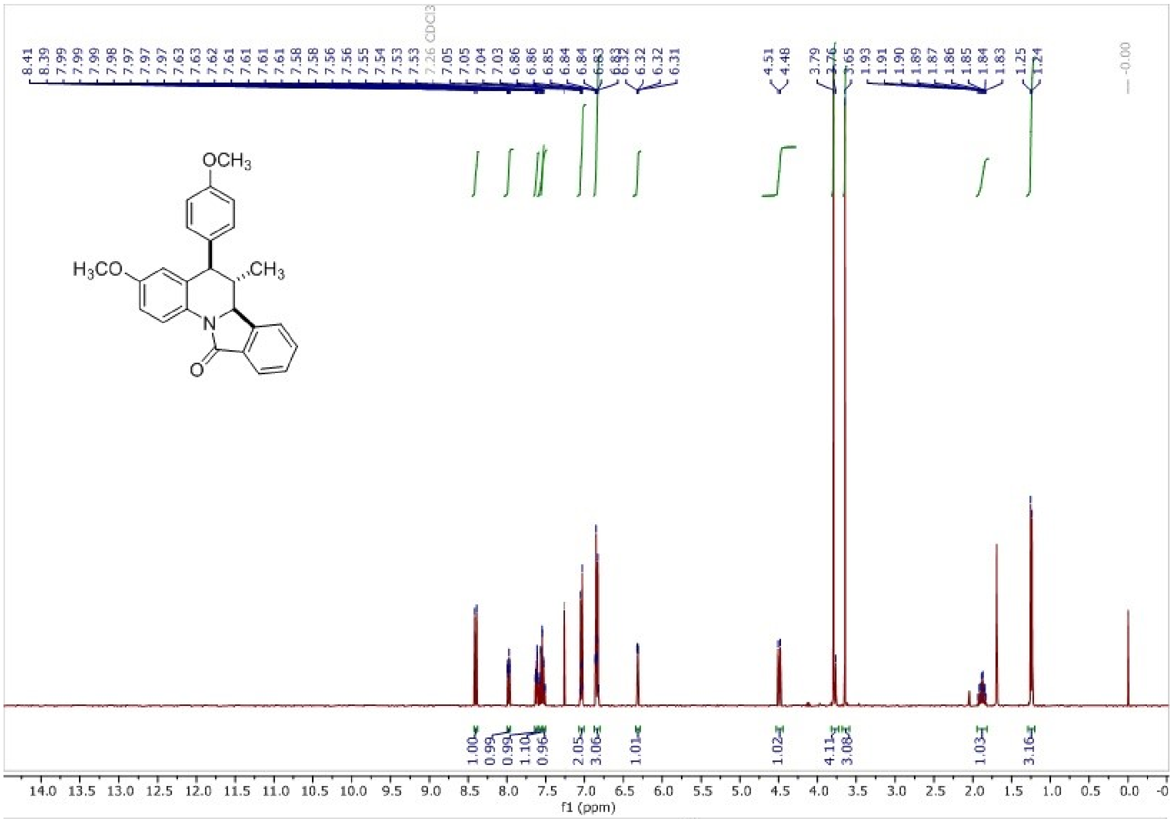

8.41 8.39 7.99 7.99 7.99 7.98 7.97 7.97 7.97 7.63 7.63 7.62 7.61 7.61 7.61 7.58 7.56 7.56 7.55 7.54 7.53 7.53 7.26 CDCl3 7.05 7.05 7.04 7.03 6.86 6.86 6.85 6.84 6.84 6.83 6.83 6.32 6.32 6.31
4.51 4.48 3.79 3.76 3.65 1.93 1.91 1.90 1.89 1.87 1.86 1.85 1.84 1.83 1.25 1.24
-0.00
OCH3
H3CO
CH3
N
O
1.00 0.99 0.99 1.10 0.96 2.05 3.06 1.01 1.02 4.11 3.08 1.03 3.16
f1 (ppm)

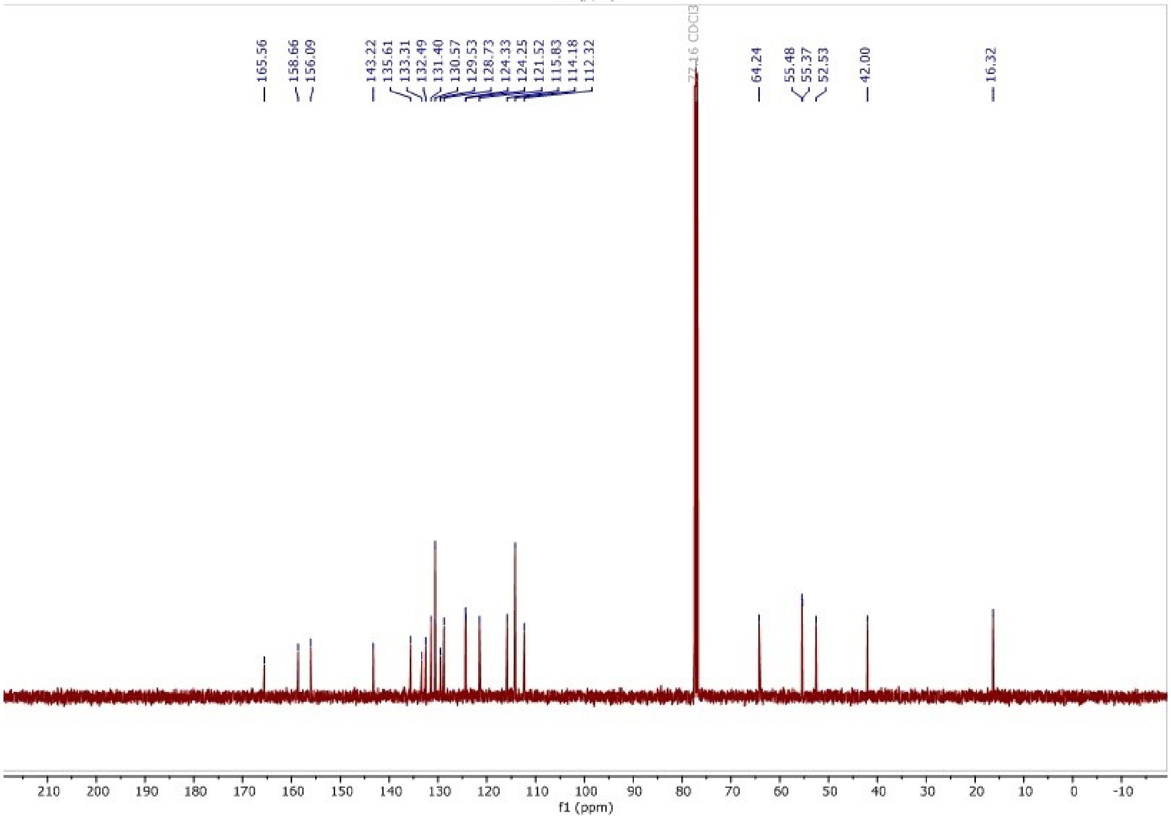

165.56 158.66 156.09 143.22 135.61 133.31 132.49 131.40 130.57 129.53 128.73 124.33 124.25 121.52 115.83 114.18 112.32
77.16 CDCl3
64.24 55.48 55.37 52.53 42.00 16.32
f1 (ppm)

***Trans*-3-ethyl-5-(4-methoxyphenyl)-6-methyl-6,6a-dihydroisoindolo[2,1-a]quinolin-11(5H)-one (4)**

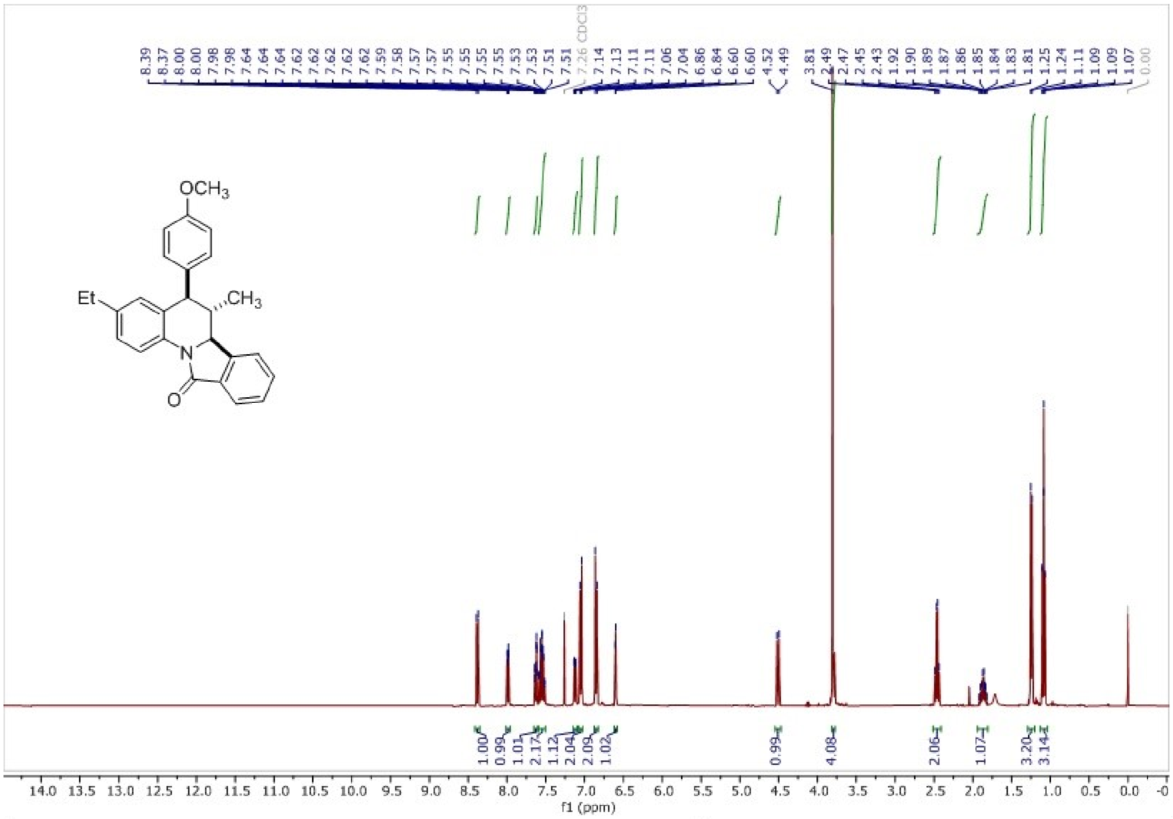

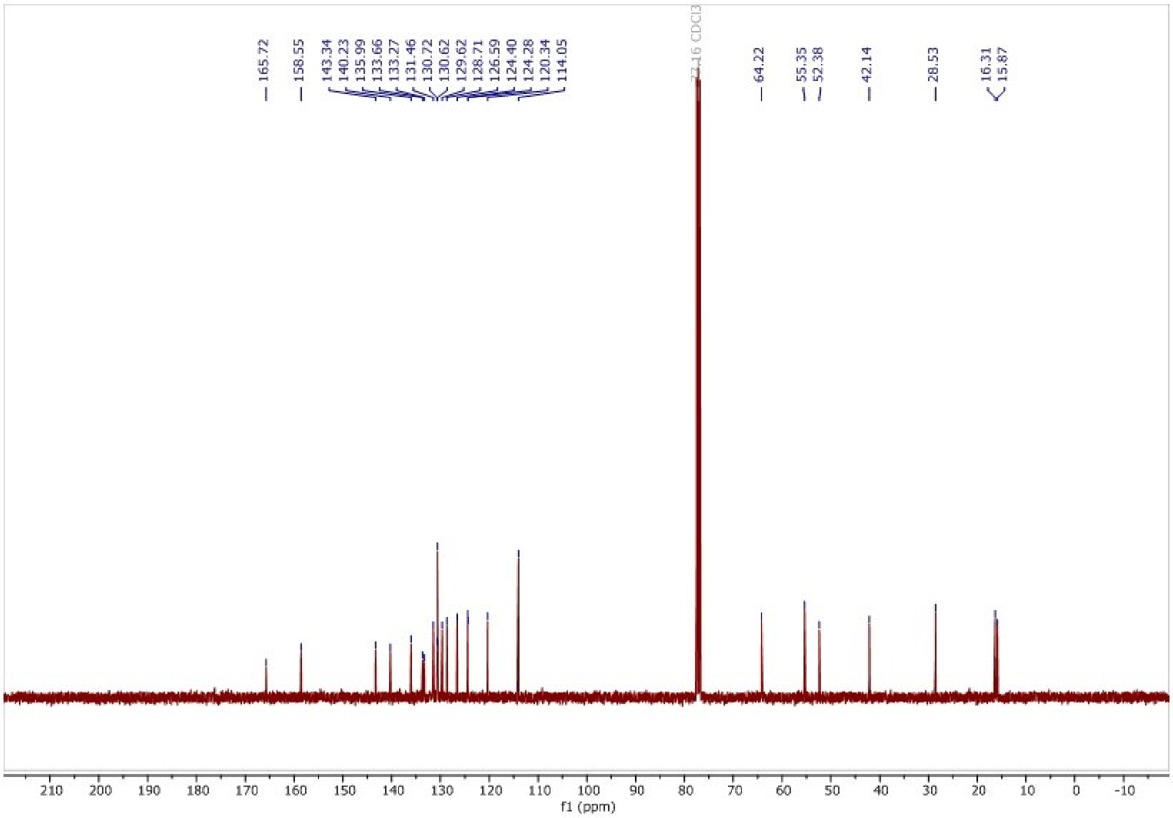

***Trans*-5-(4-methoxyphenyl)-2,4,6-trimethyl-6,6a-dihydroisoindolo[2,1-a]quinolin-11(5H)-one (5)**

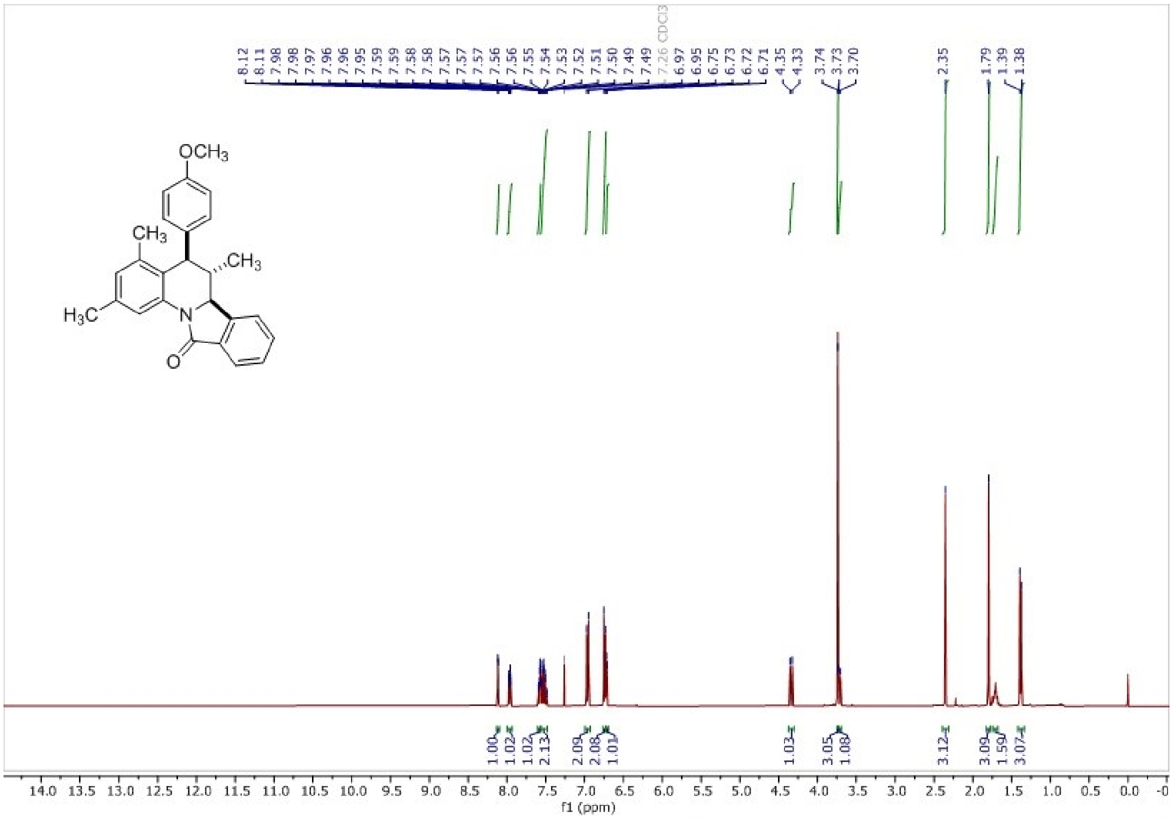

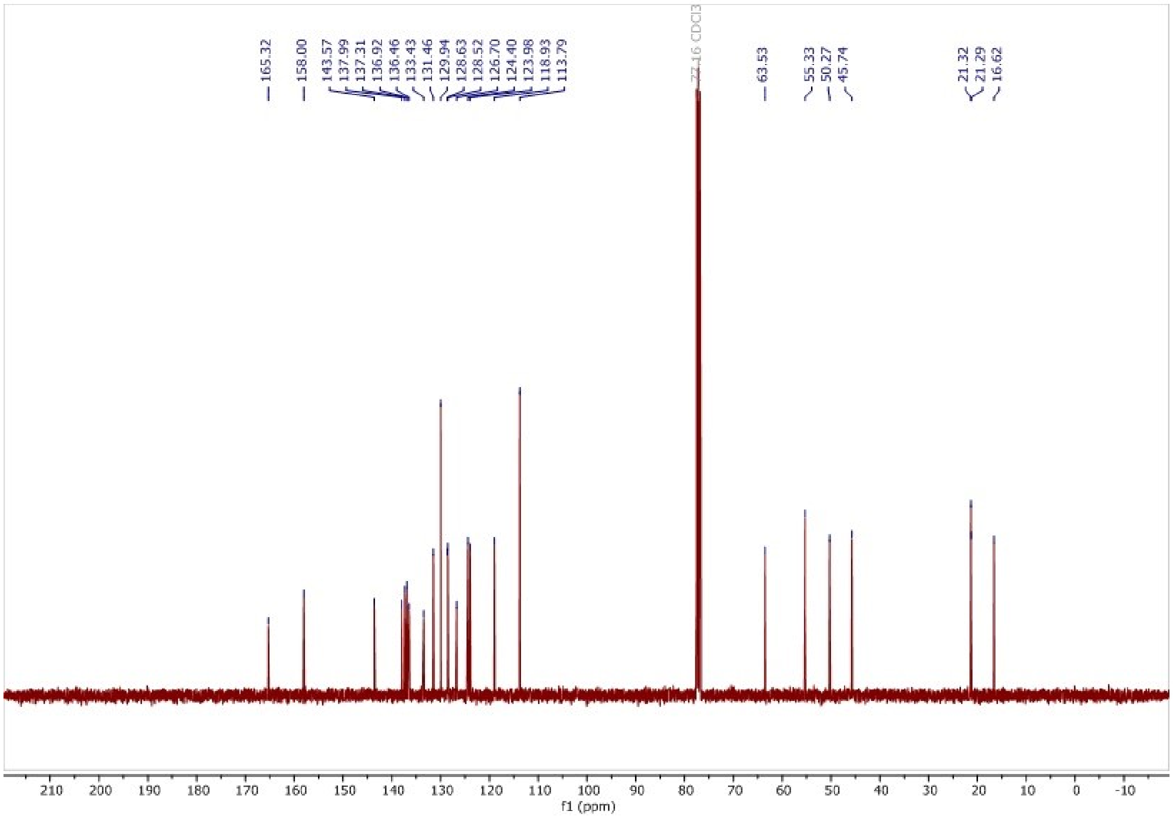

***Trans*-2,3,4-trimethoxy-5-(4-methoxyphenyl)-6-methyl-6,6a-dihydroisoindolo[2,1-a]quinolin-11(5H)-one (6).**

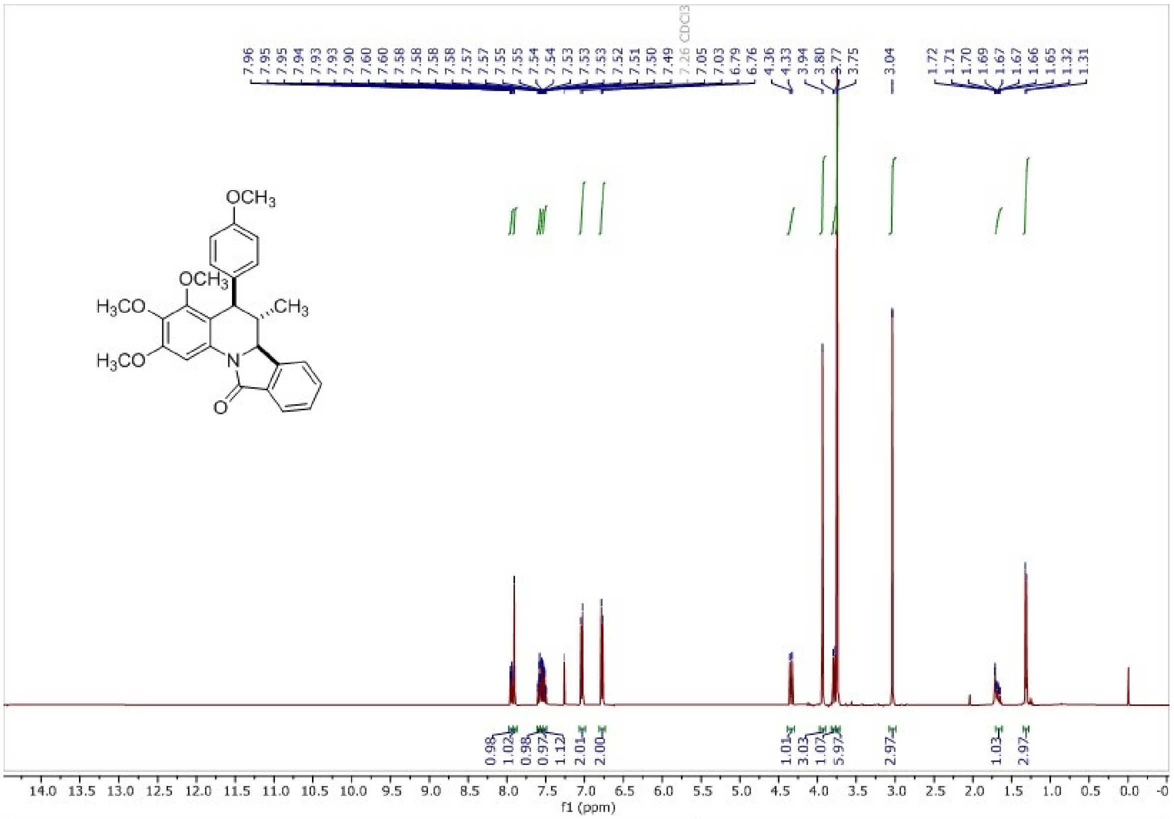

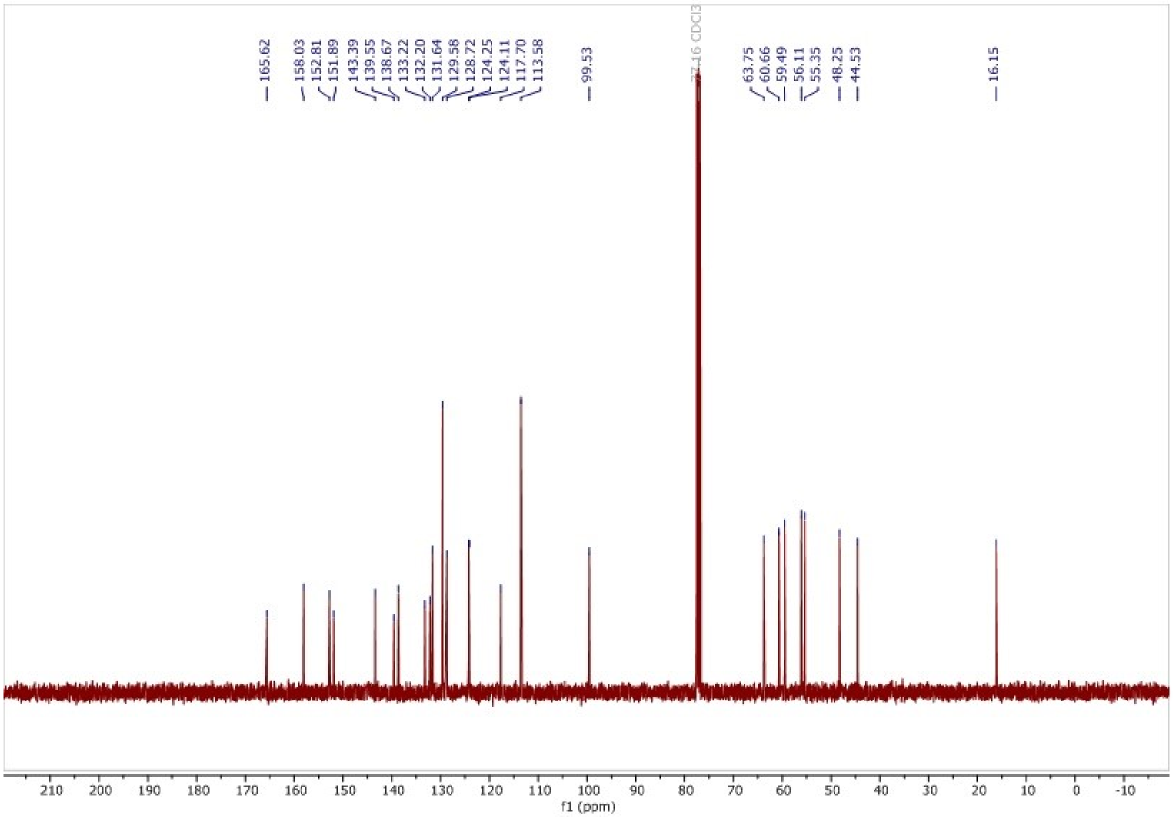

***Trans*-5-(4-methoxyphenyl)-6-methyl-3-nitro-6,6a-dihydroisoindolo[2,1-a]quinolin-11(5H)-one (7)**.

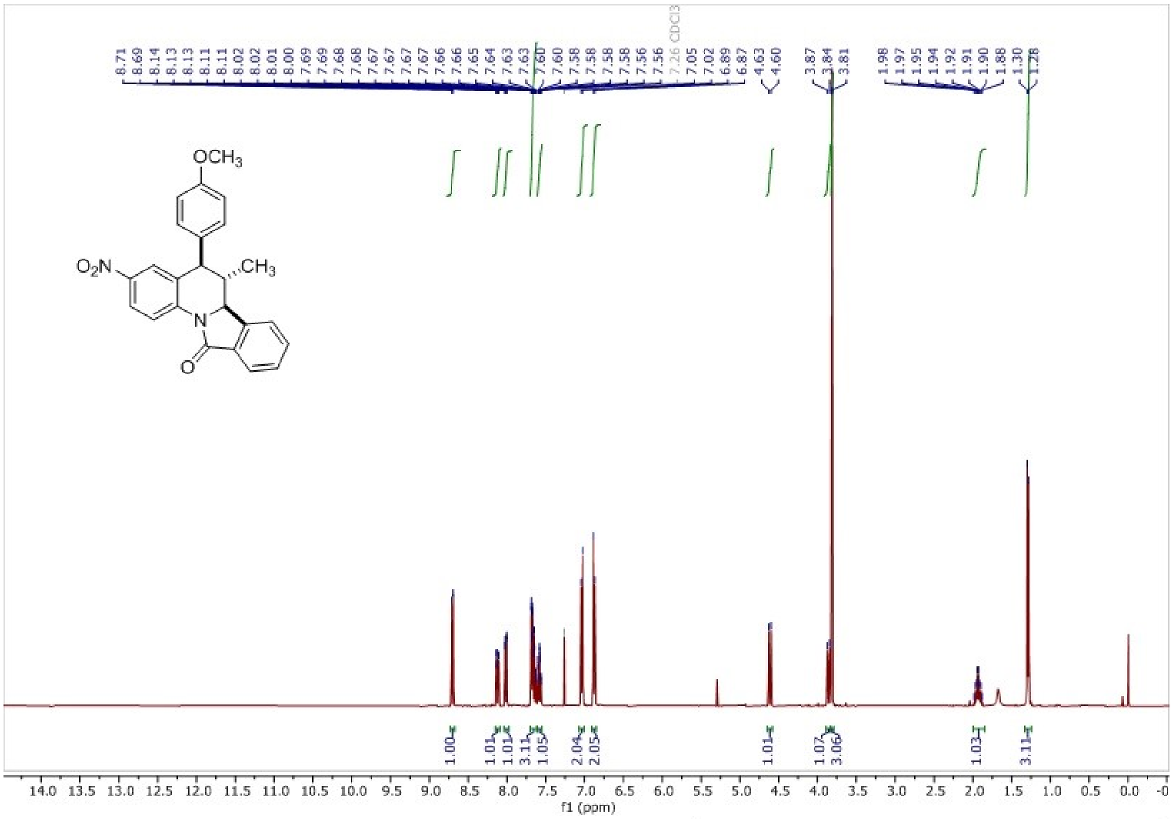

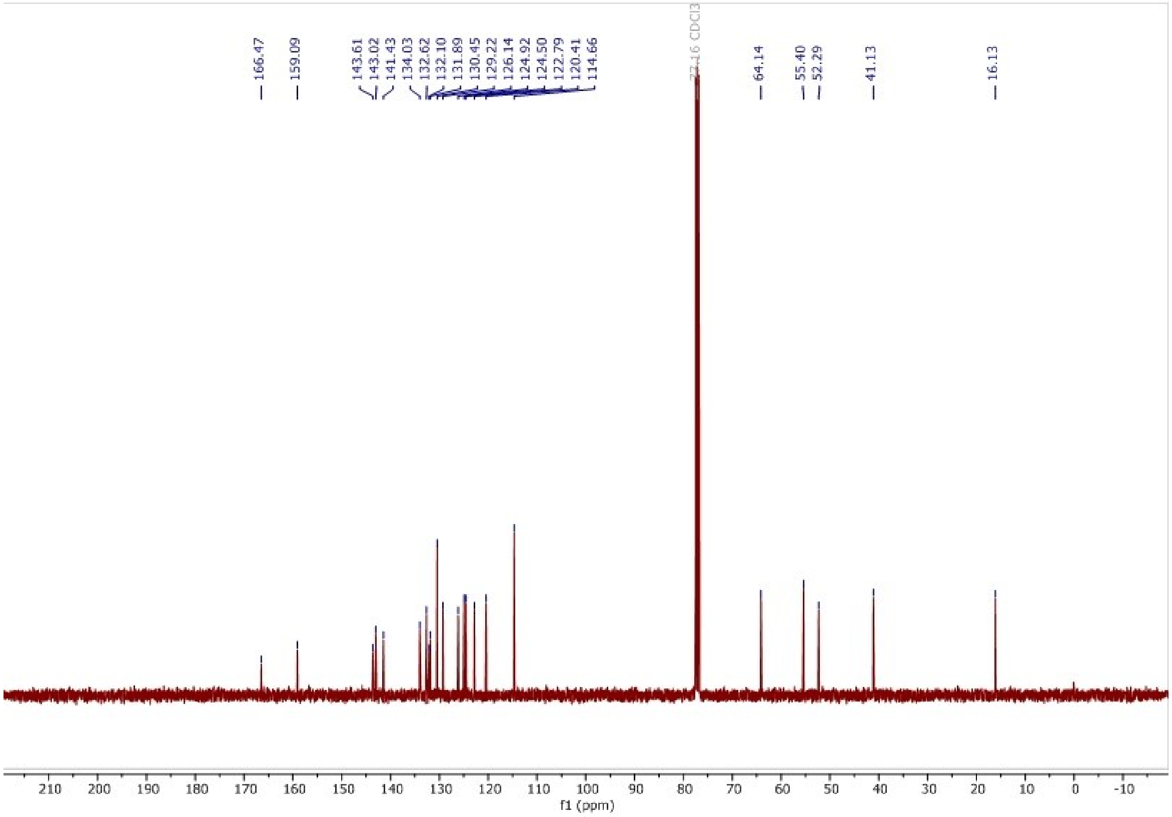

***Trans*-3-iodo-5-(4-methoxyphenyl)-6-methyl-6,6a-dihydroisoindolo[2,1-a]quinolin-11(5H)-one (8)**.

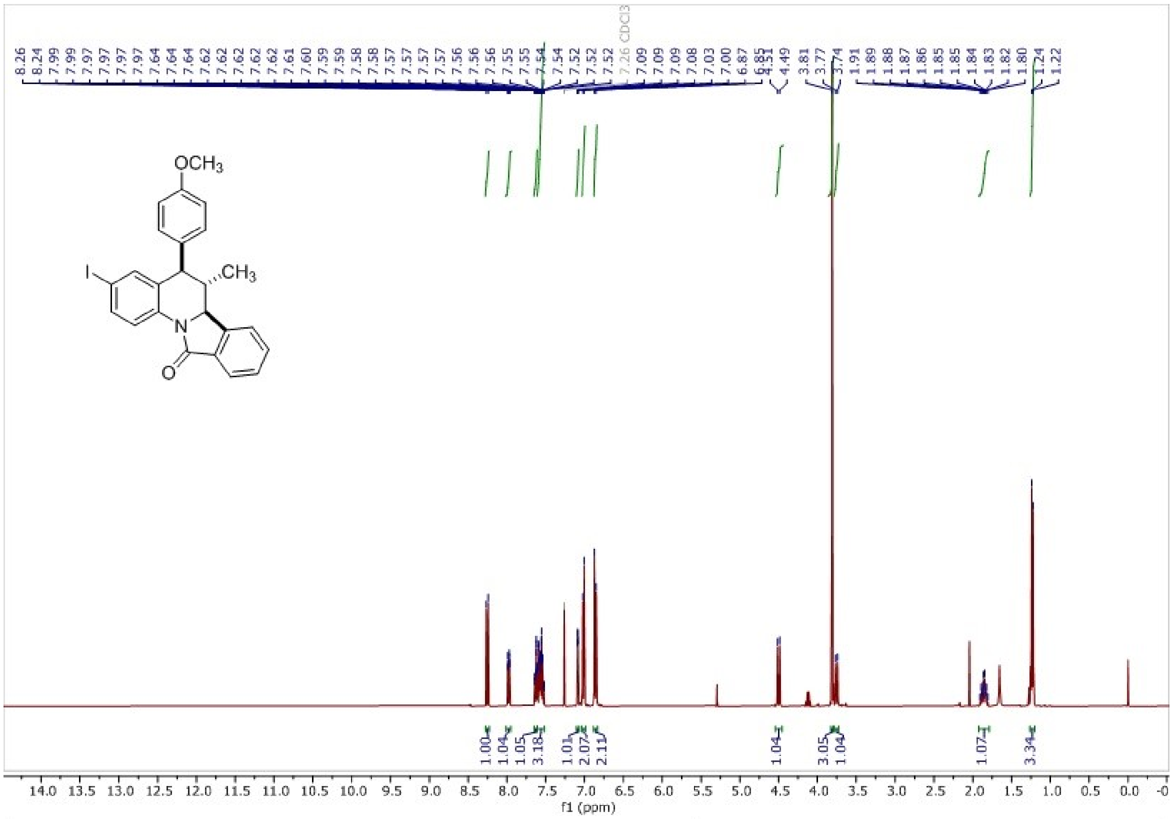

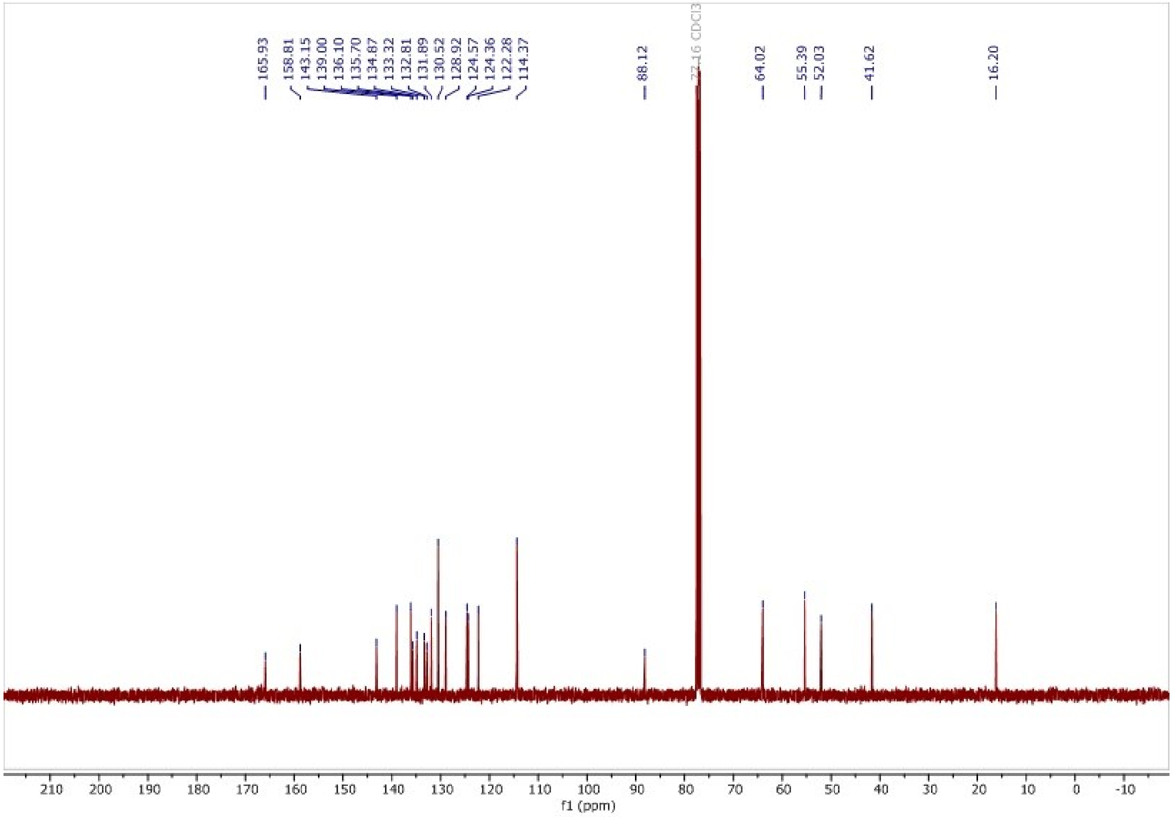

***Trans*-3-bromo-5-(4-methoxyphenyl)-6-methyl-6,6a-dihydroisoindolo[2,1-a]quinolin-11(5H)-one (9)**.

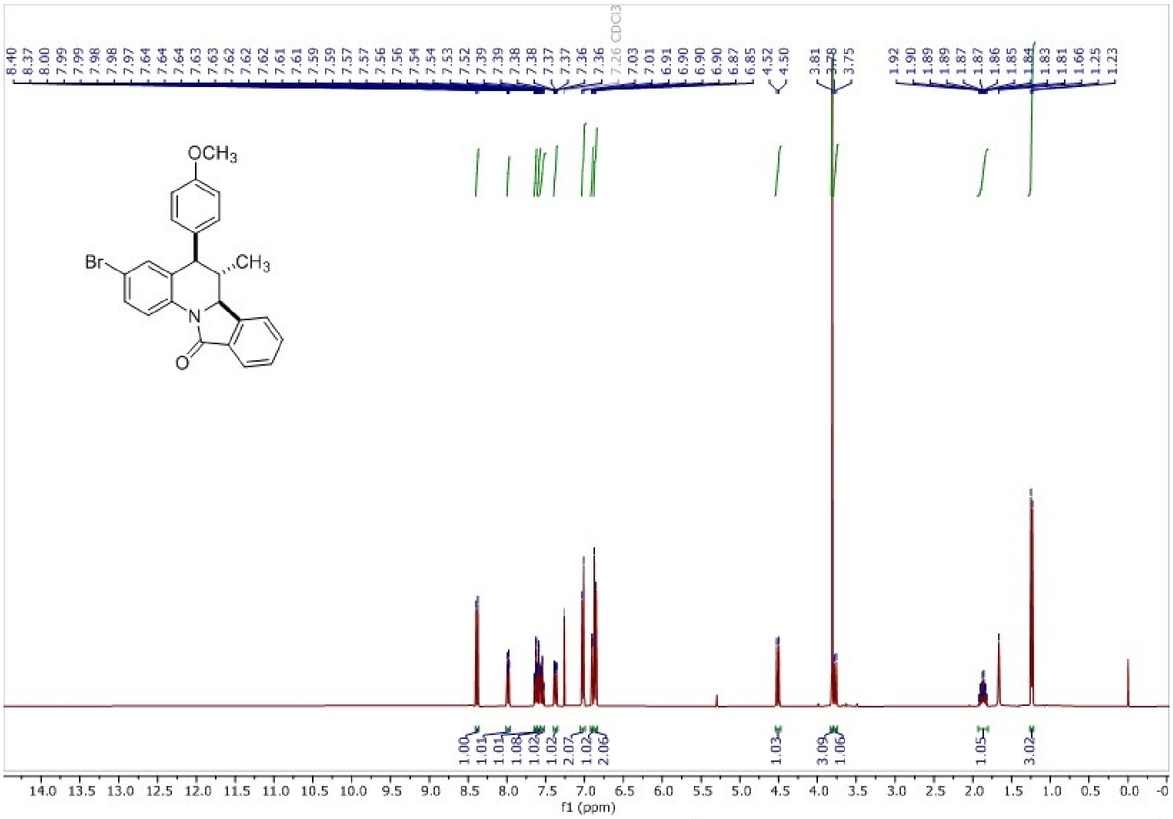

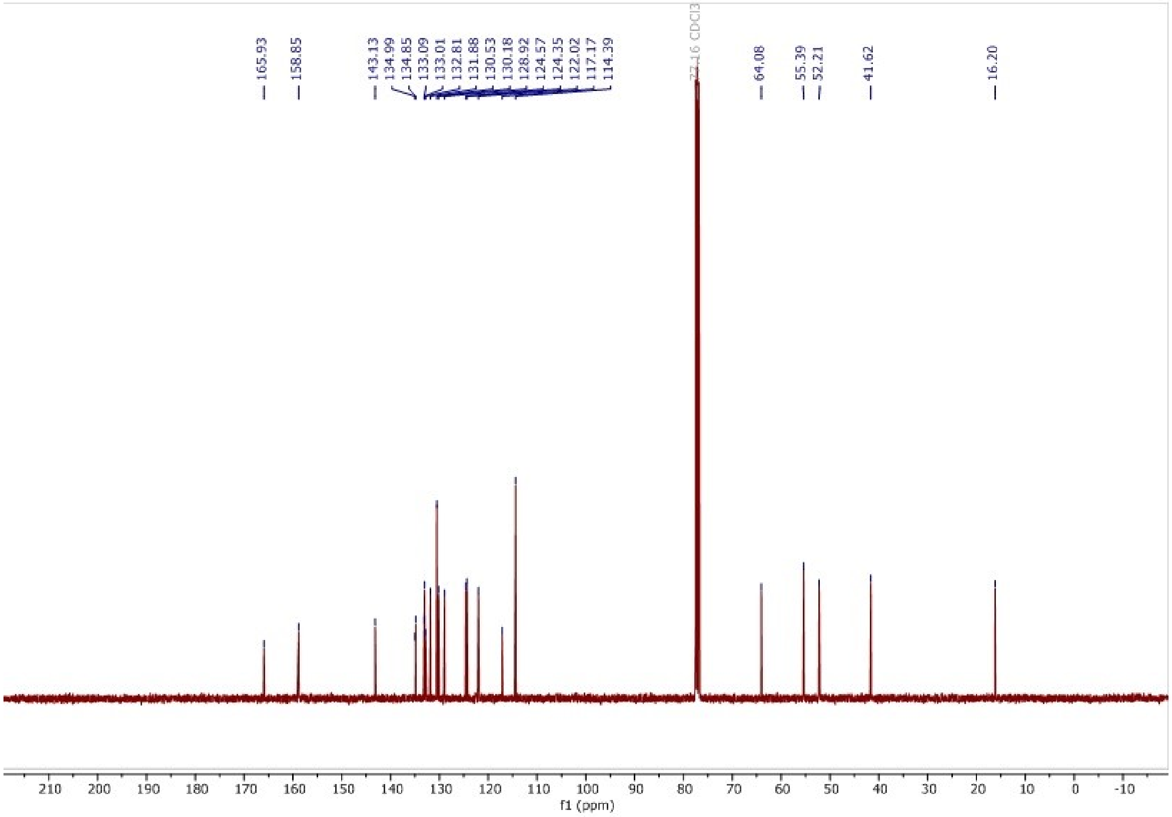

***Trans*-3-chloro-5-(4-methoxyphenyl)-6-methyl-6,6a-dihydroisoindolo[2,1-a]quinolin-11(5H)-one (10)**.

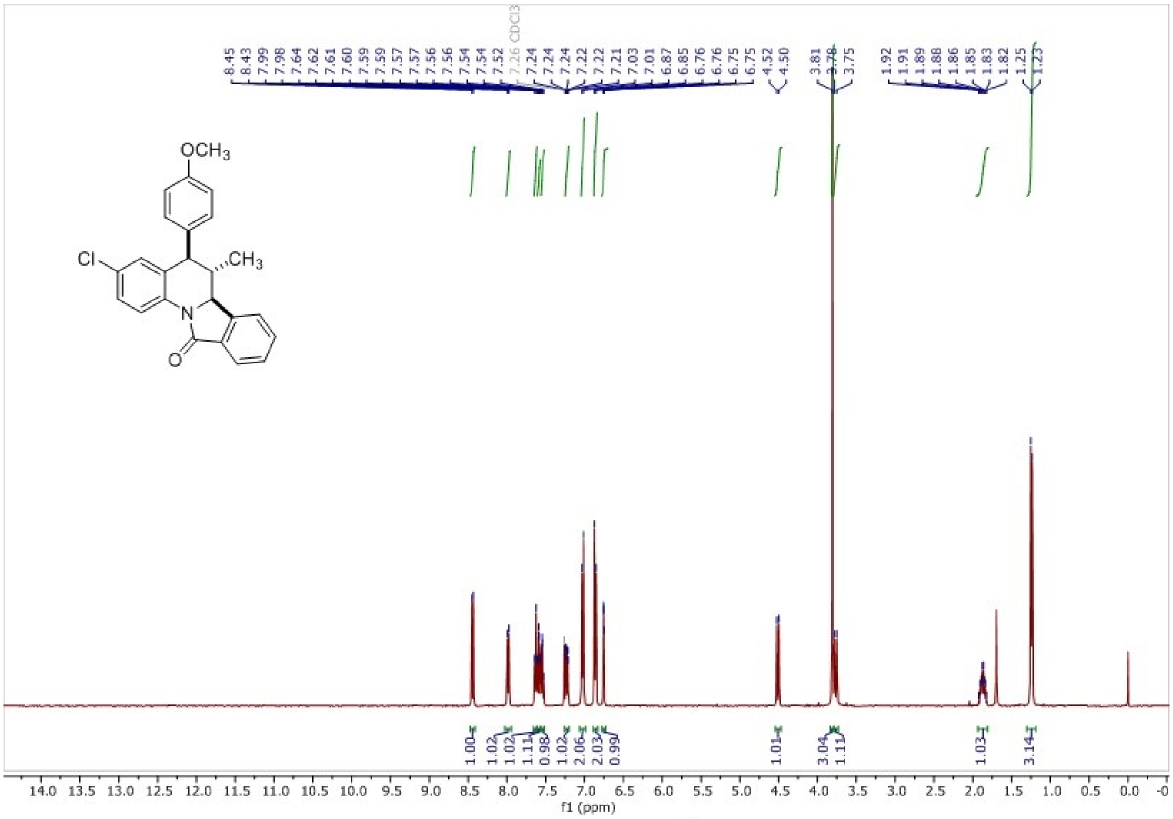

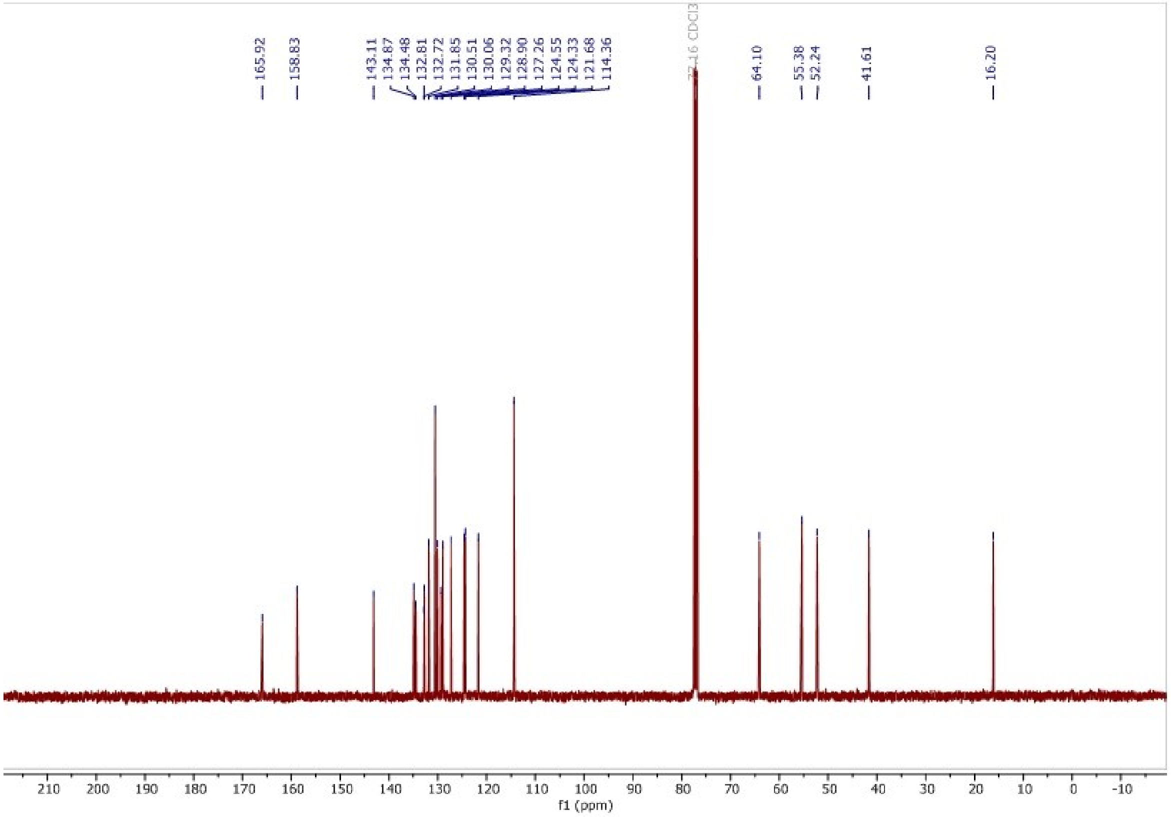

***Trans*-3-fluoro-5-(4-methoxyphenyl)-6-methyl-6,6a-dihydroisoindolo[2,1-a]quinolin-11(5H)-one (11)**.

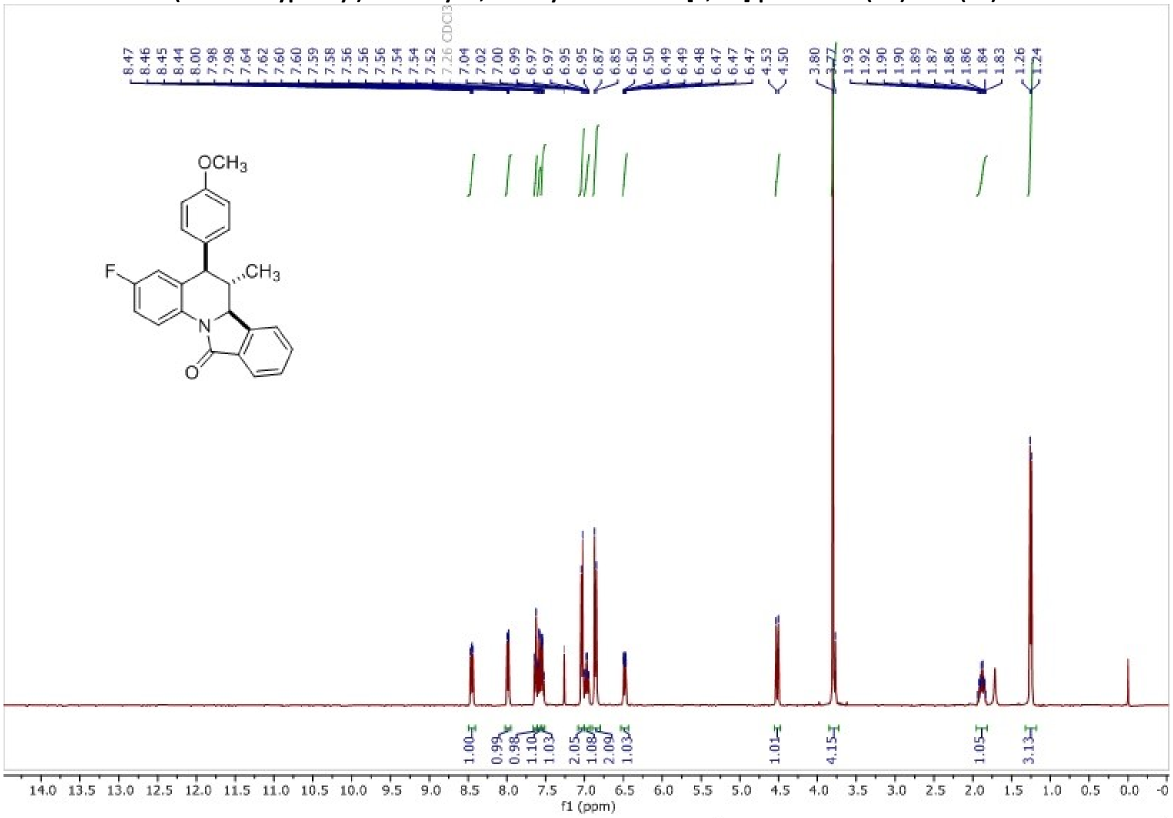

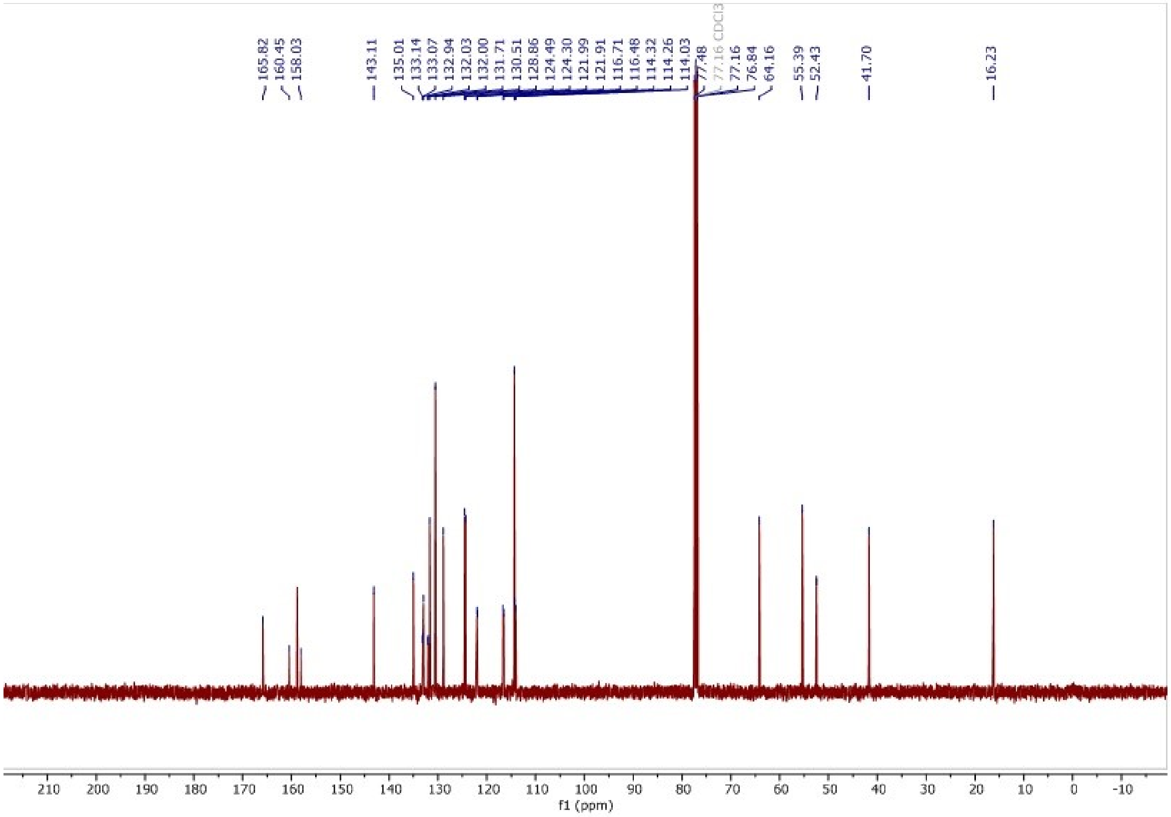

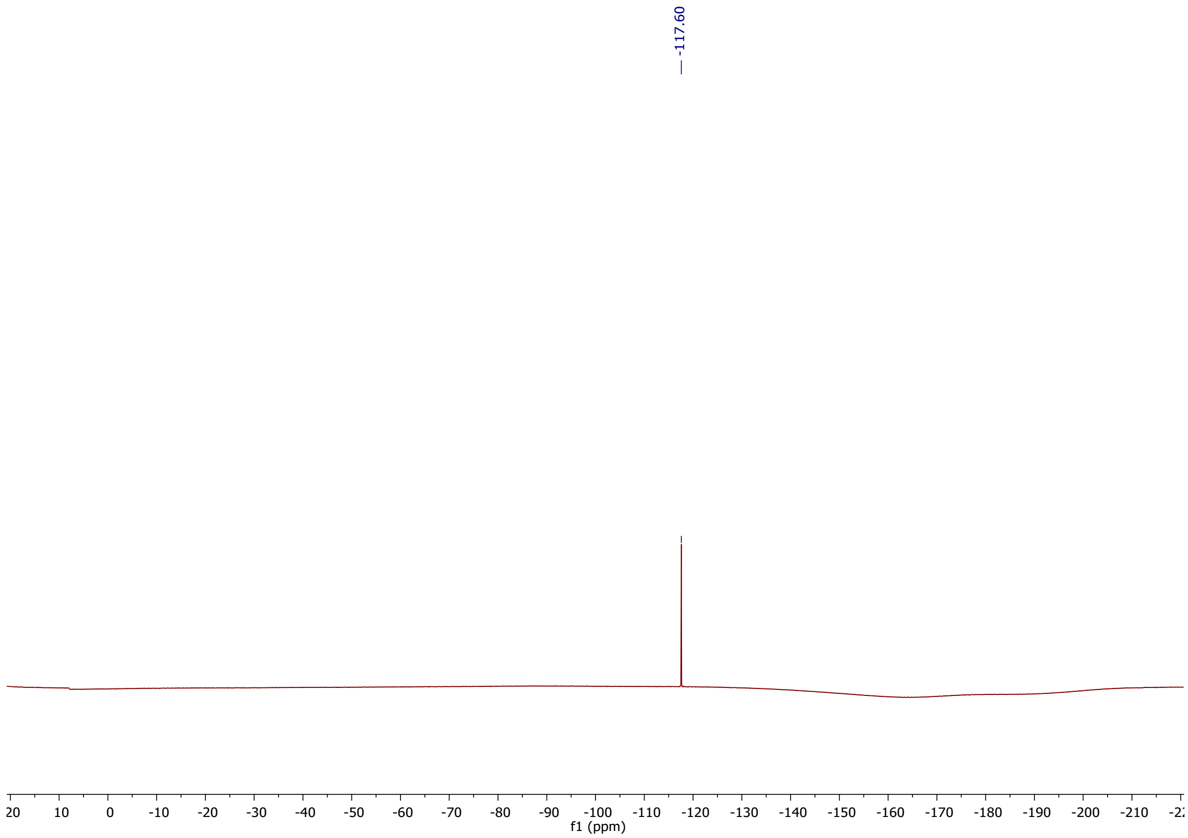
-117.60
20 10 0 -10 -20 -30 -40 -50 -60 -70 -80 -90 -100 -110 -120 -130 -140 -150 -160 -170 -180 -190 -200 -210
f1 (ppm)

***Trans*-1-methoxy-5-(4-methoxyphenyl)-6-methyl-6,6a-dihydroisoindolo[2,1-a]quinolin-11(5H)-one (12)**.

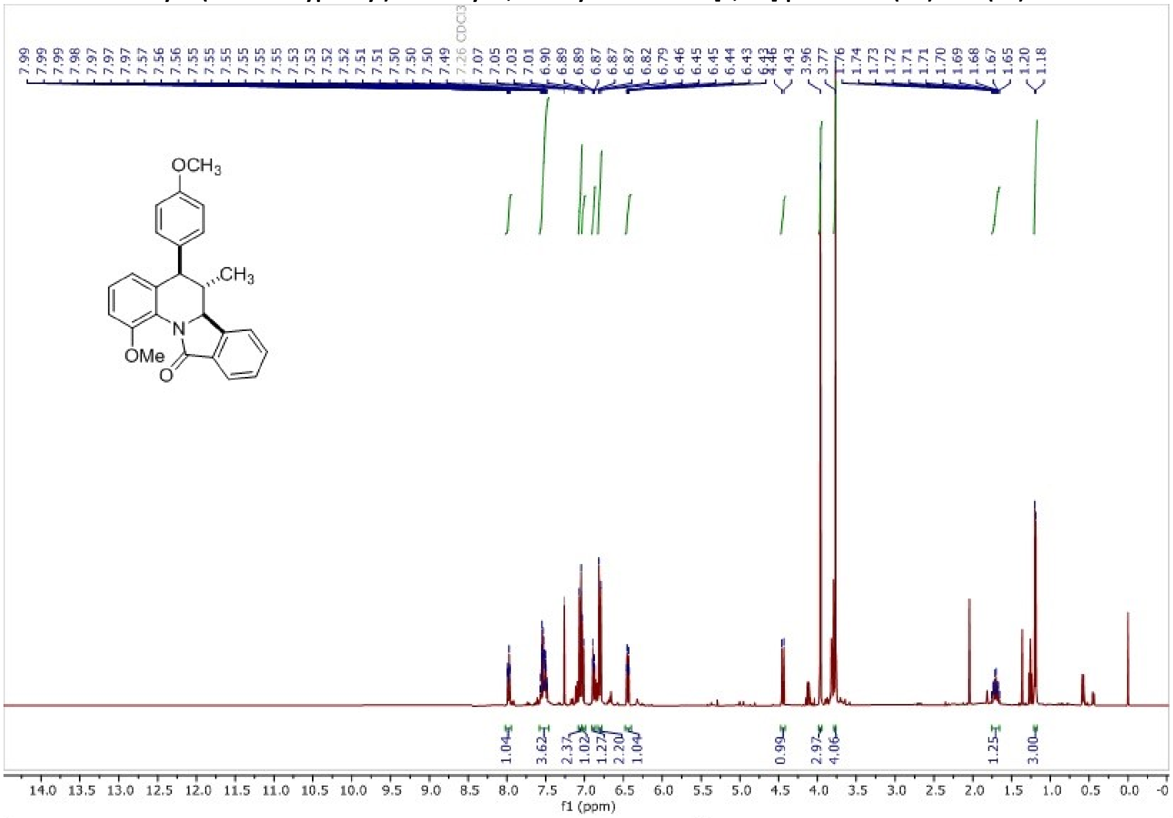

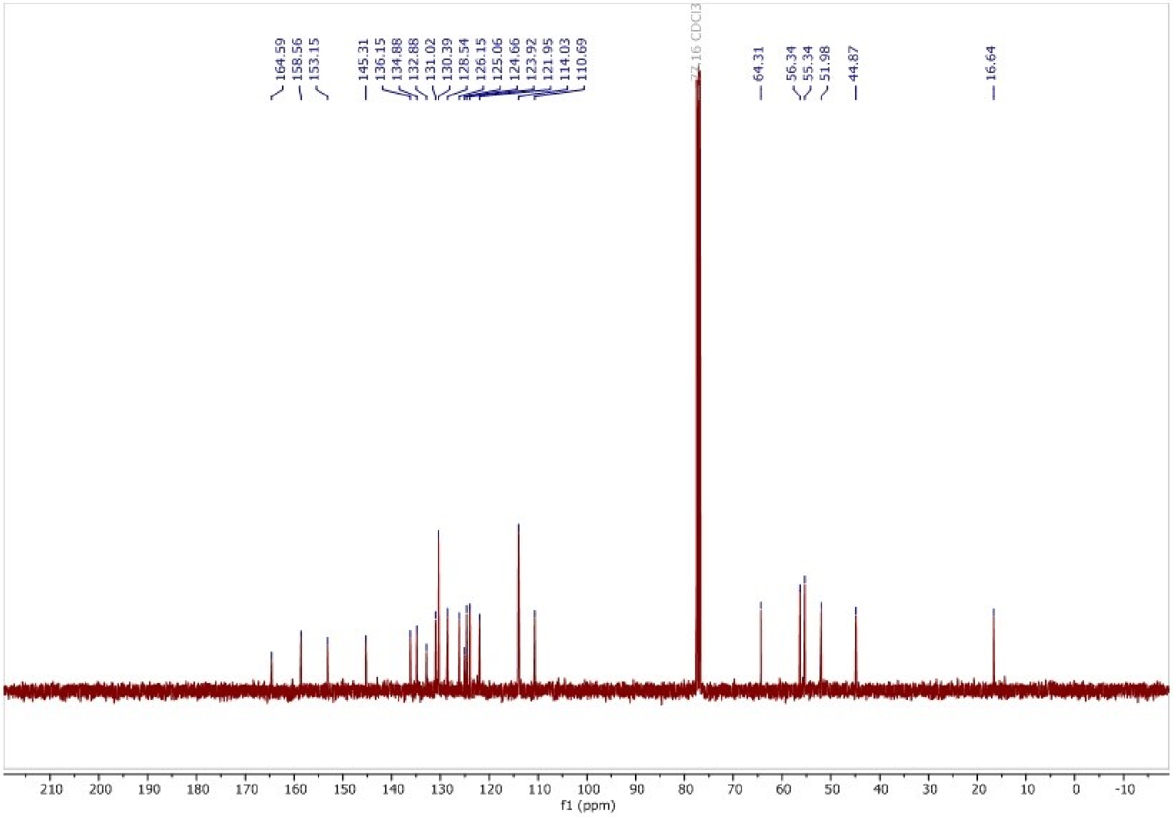

***Trans*-1-chloro-5-(4-methoxyphenyl)-6-methyl-6,6a-dihydroisoindolo[2,1-a]quinolin-11(5H)-one (13)**.

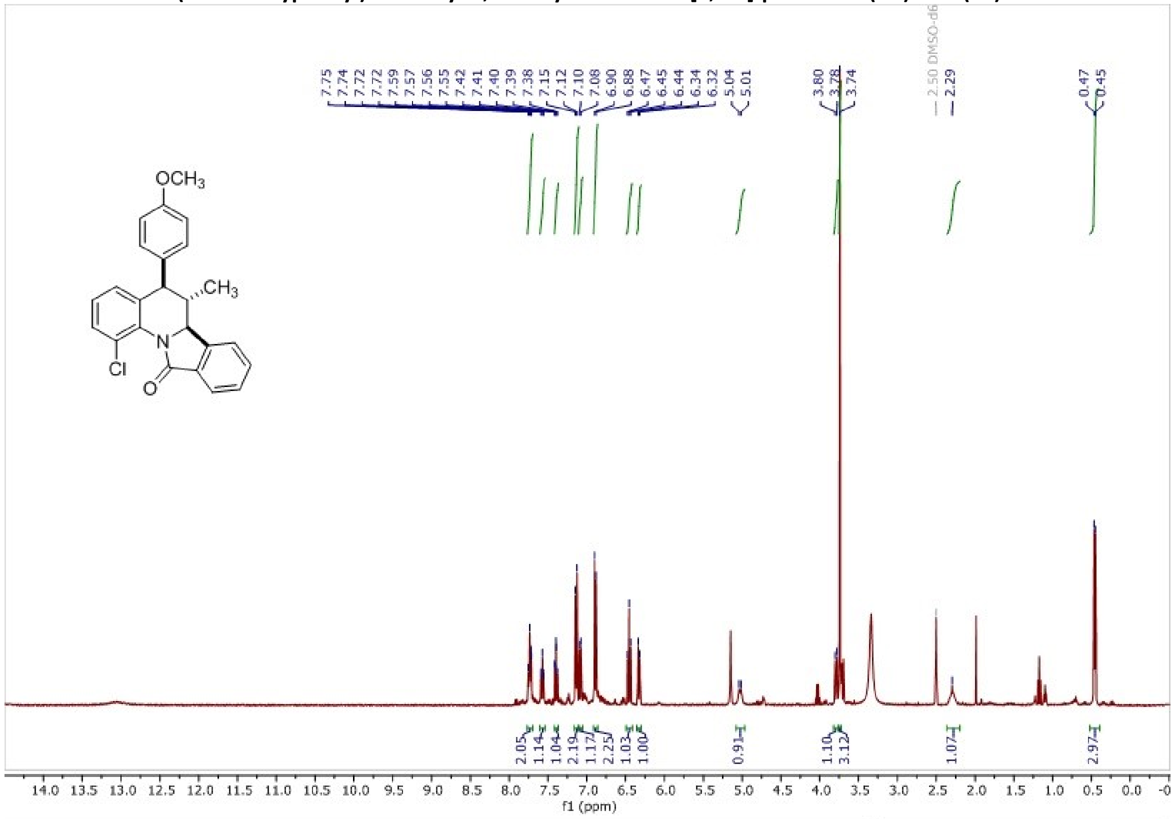

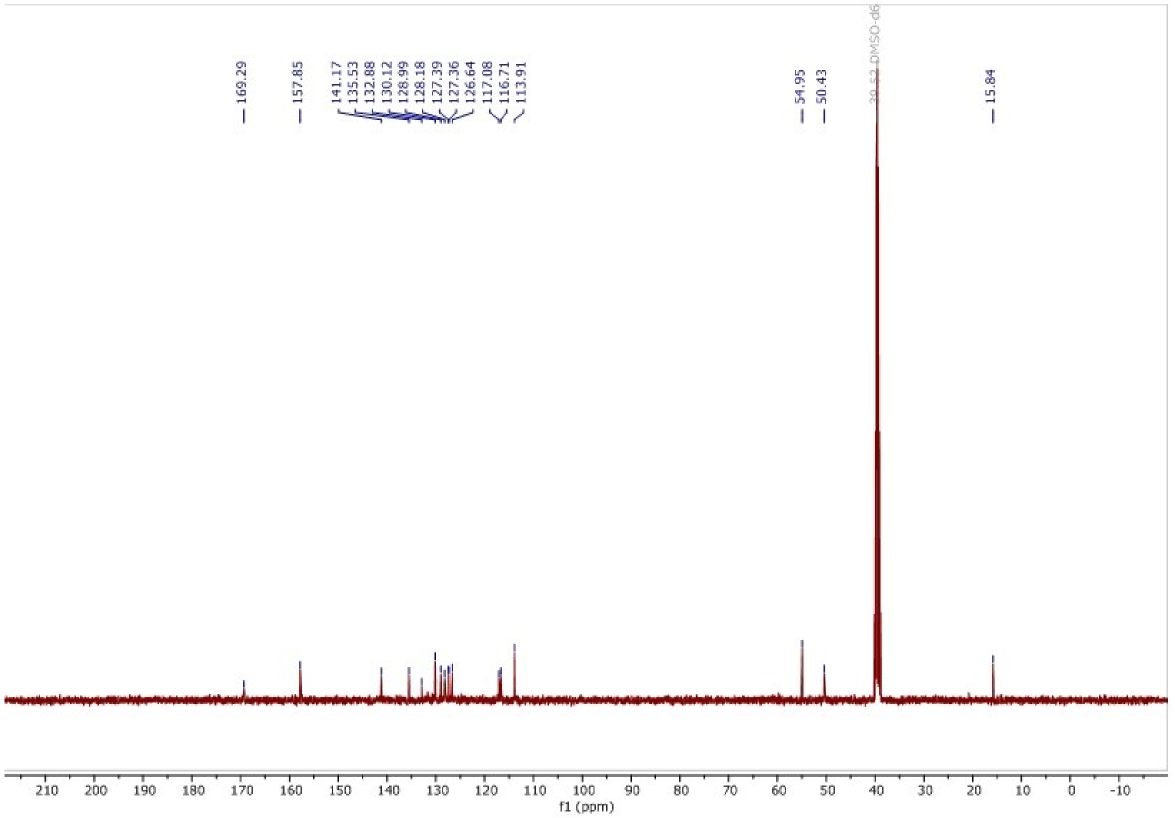

***Trans*-5-(3,4-dimethoxyphenyl)-3,6-dimethyl-6,6a-dihydroisoindolo[2,1-a]quinolin-11(5H)-one (14)**.

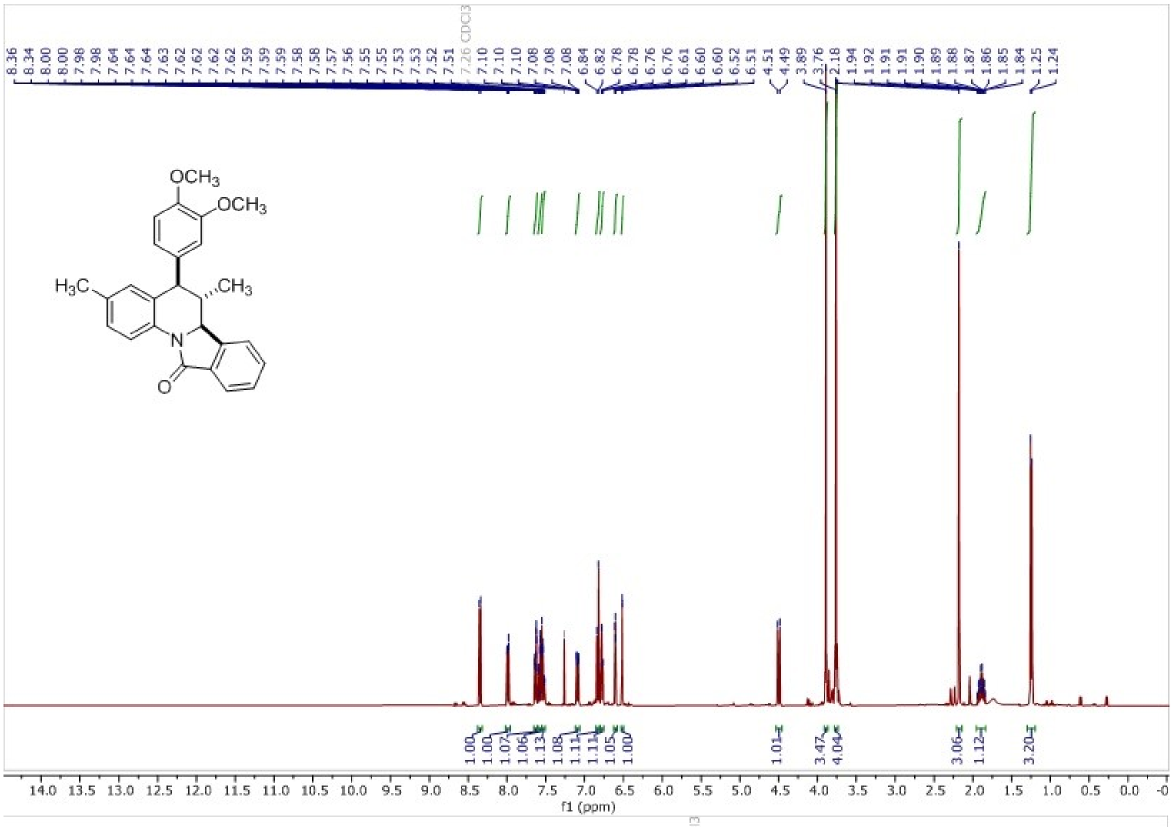

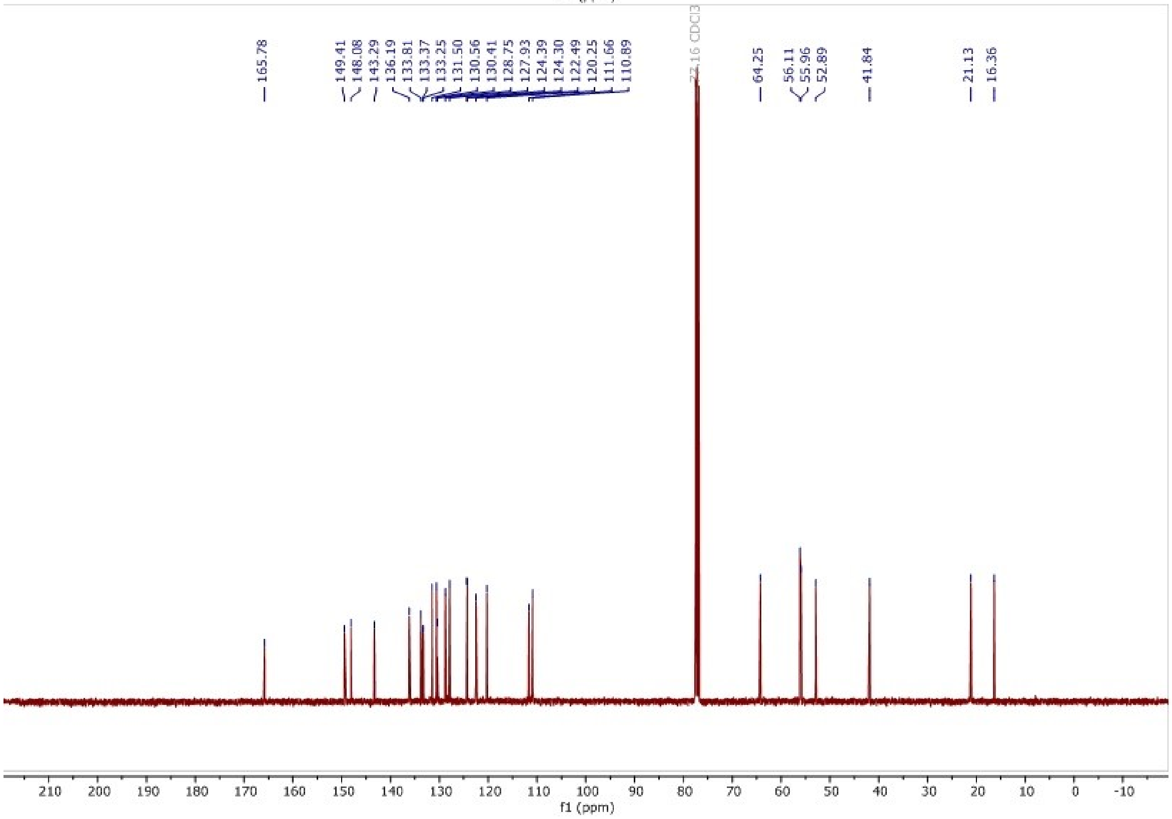

***Trans*-5-(3,4-dimethoxyphenyl)-6-methyl-3-nitro-6,6a-dihydroisoindolo[2,1-a]quinolin-11(5H)-one (15)**.

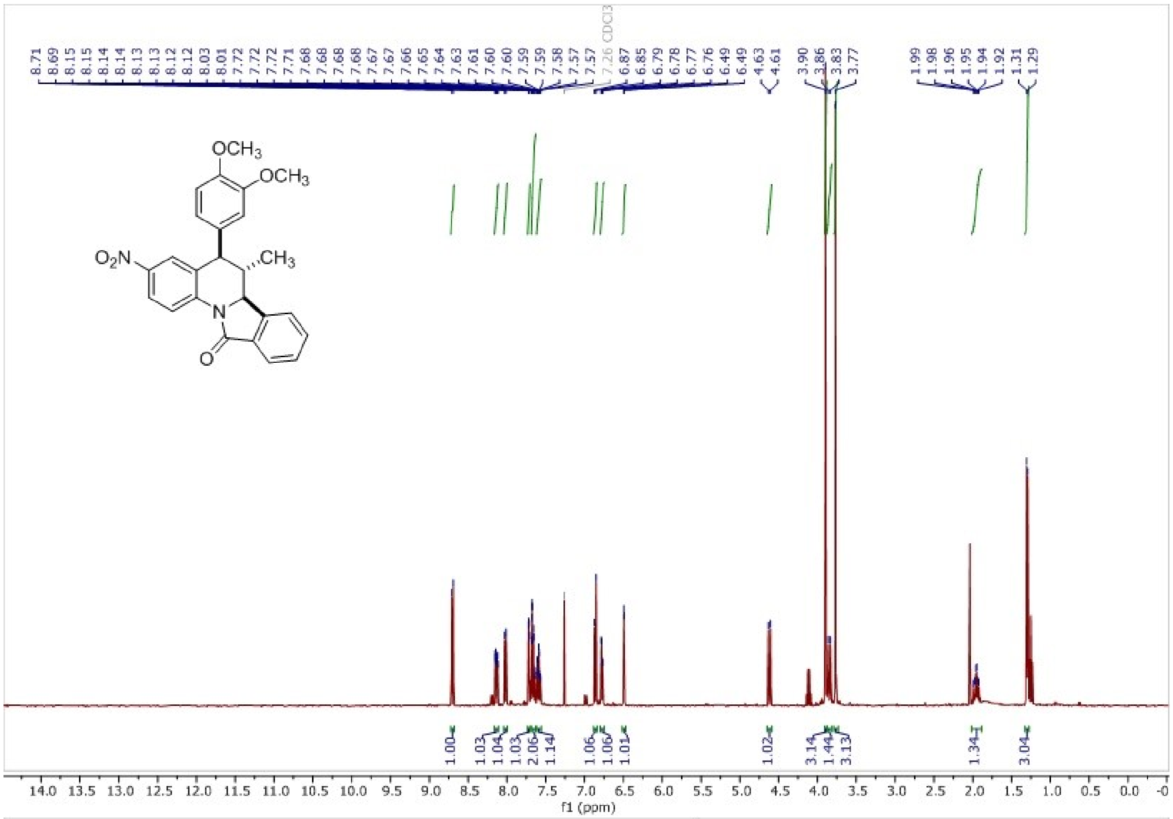

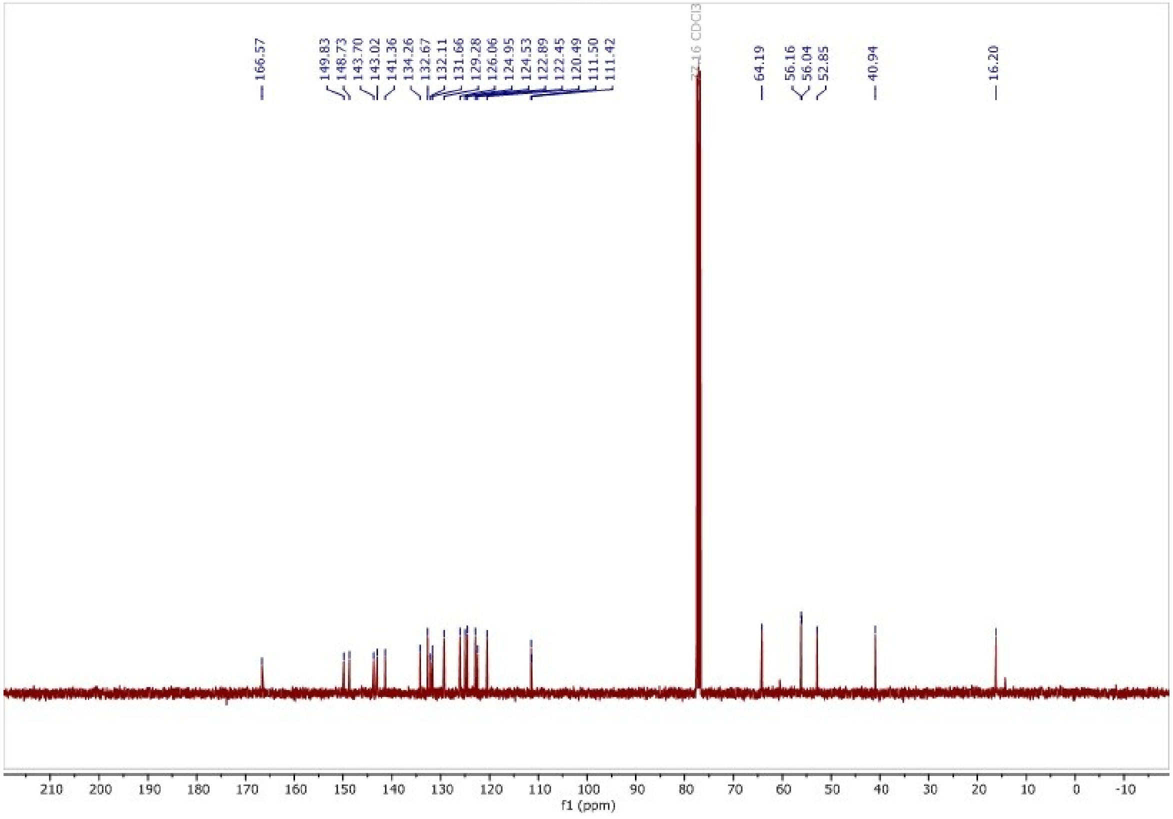

***Trans*-3-bromo-5-(3,4-dimethoxyphenyl)-6-methyl-6,6a-dihydroisoindolo[2,1-a]quinolin-11(5H)-one (16)**.

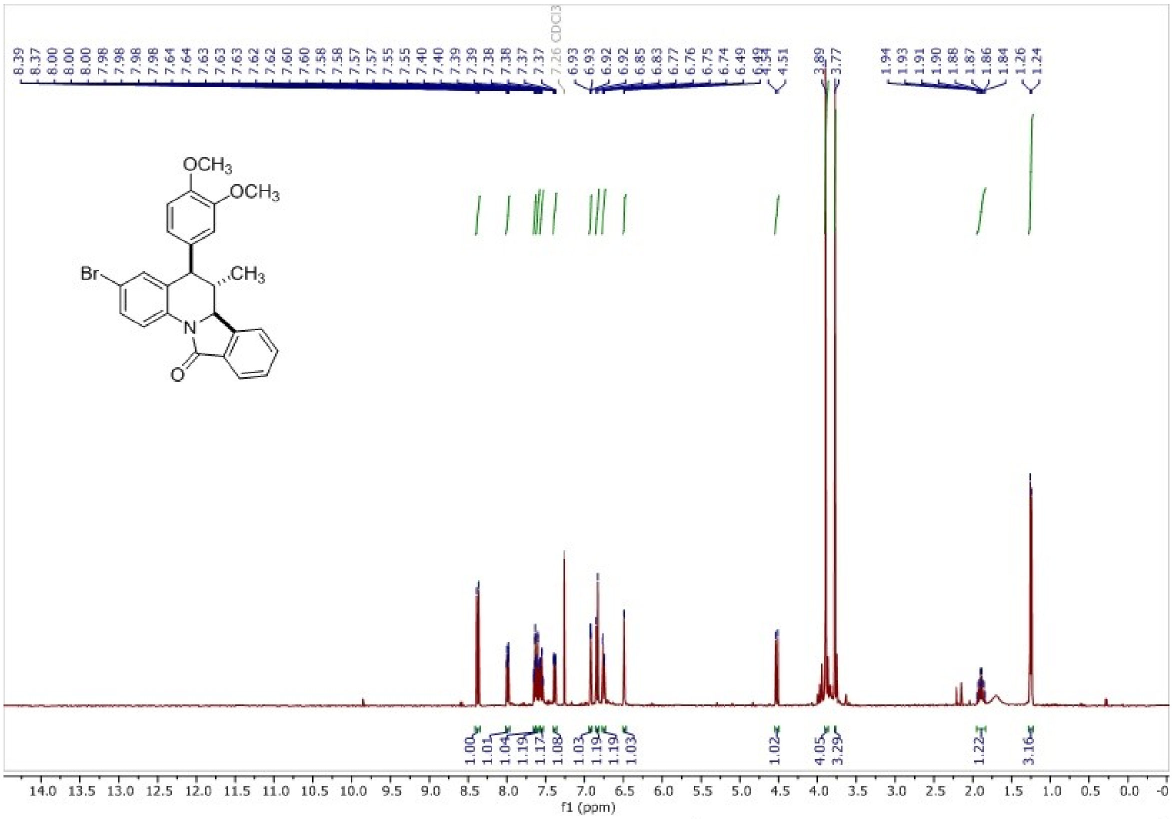

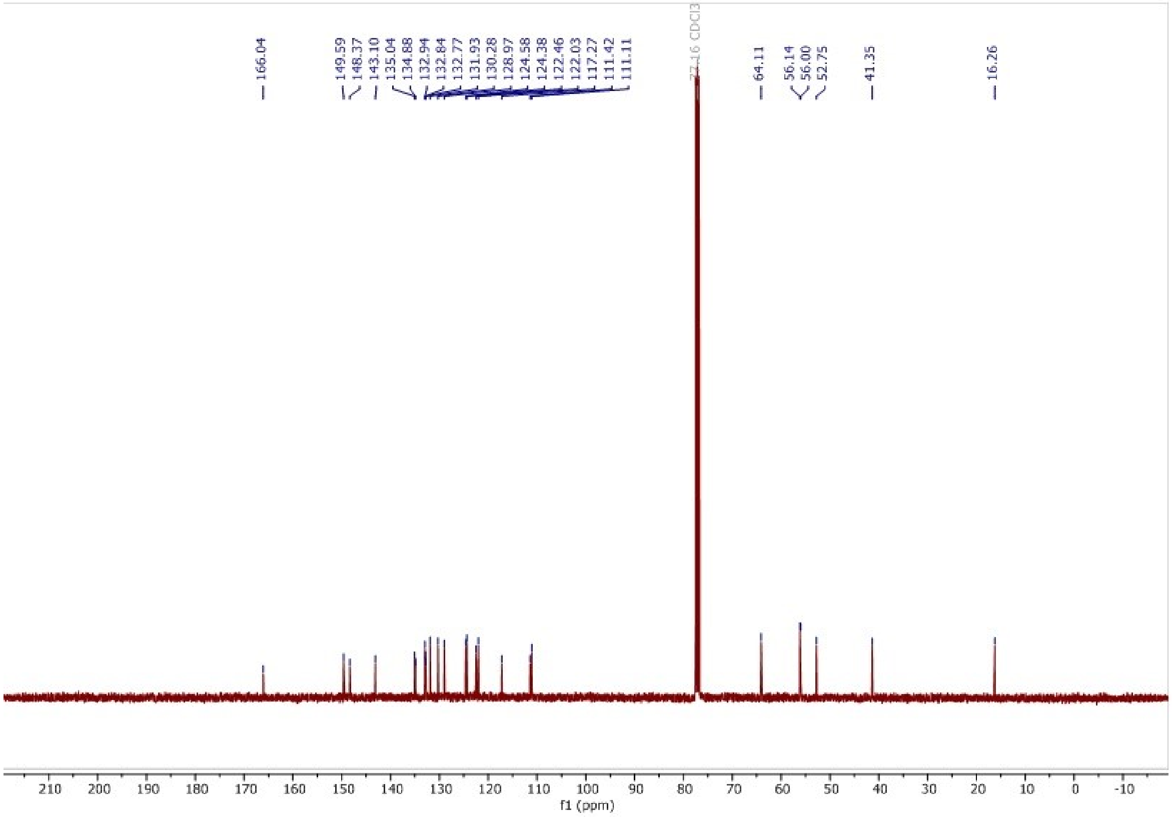

***Trans*-9-methyl-10b,15,15a,15b-tetrahydro-5H-indeno[2,1-c]isoindolo[2,1-a]quinolin-5-one (17)**.

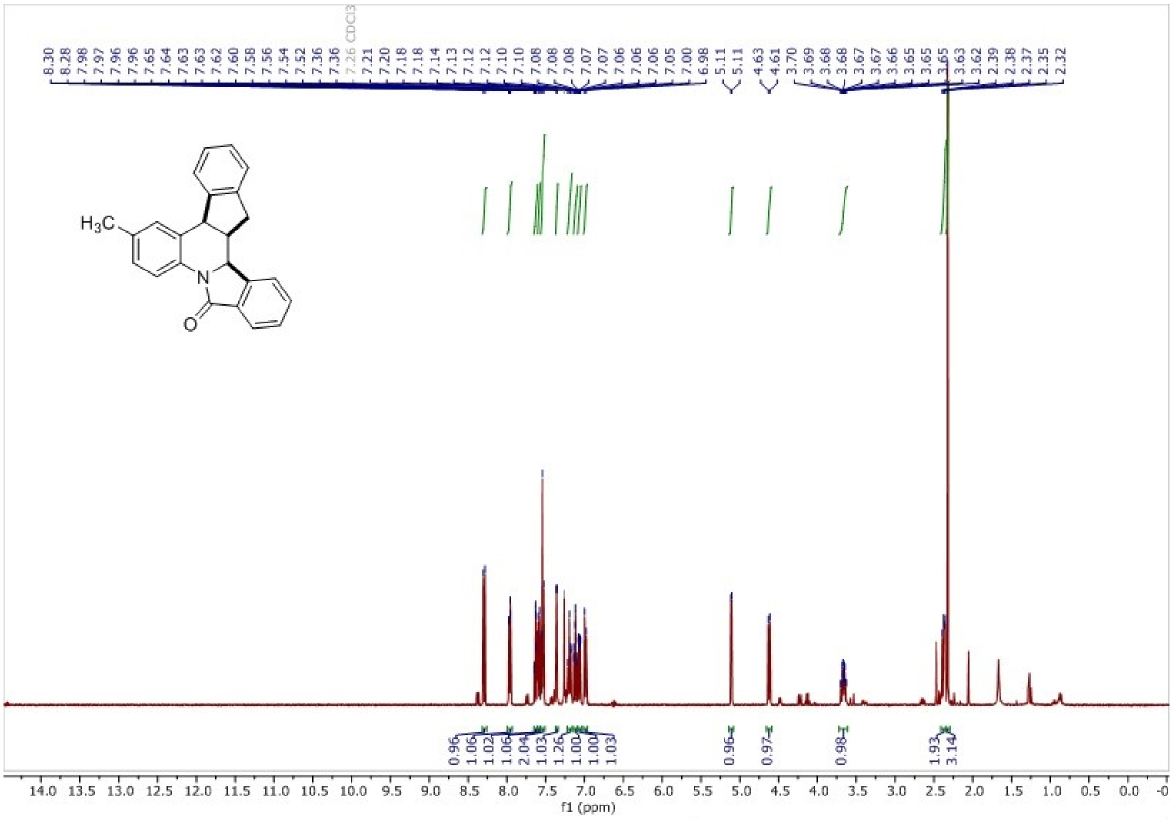

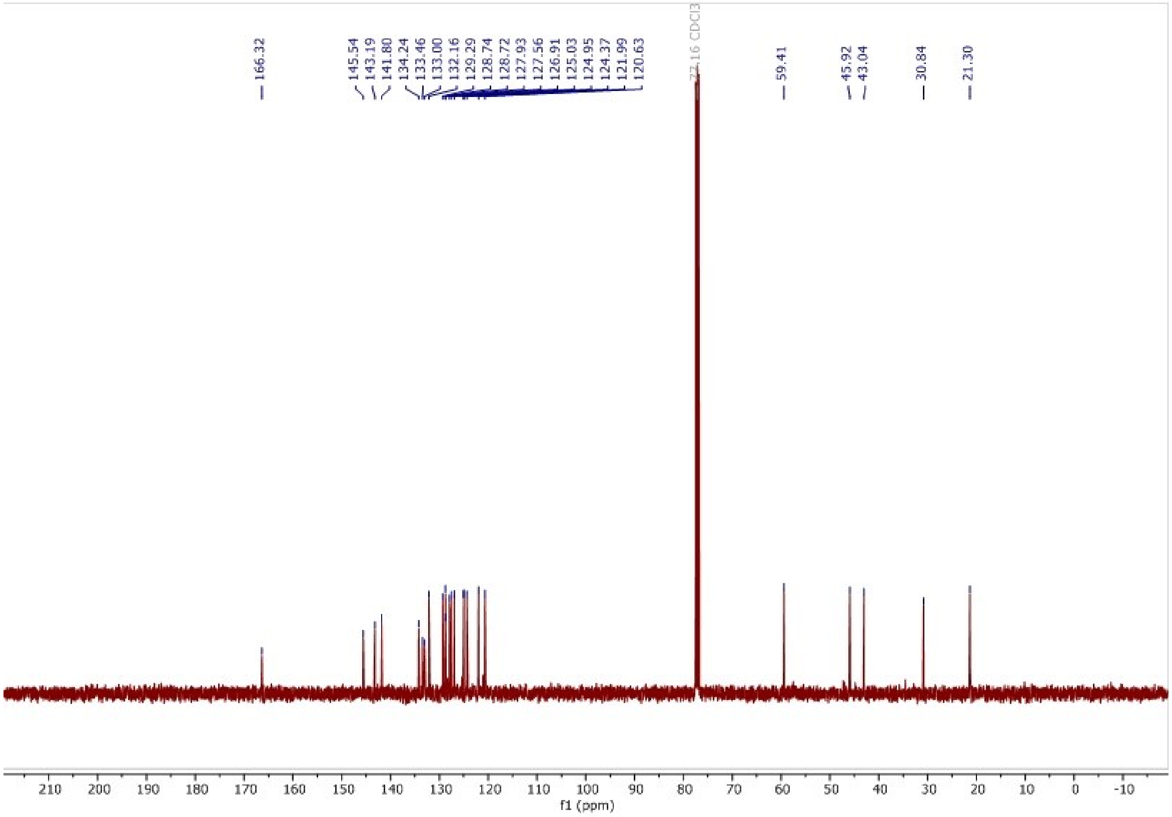

***Trans*-9-nitro-10b,15,15a,15b-tetrahydro-5H-indeno[2,1-c]isoindolo[2,1-a]quinolin-5-one (18)**.

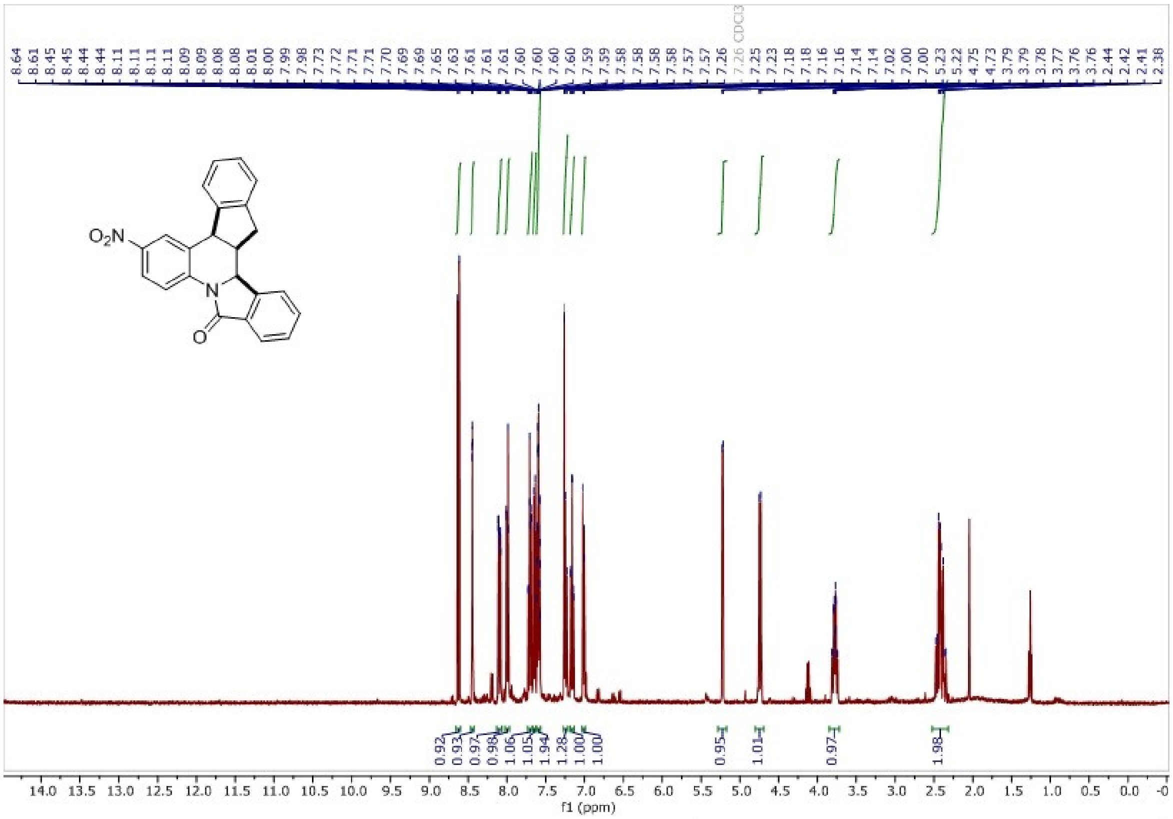

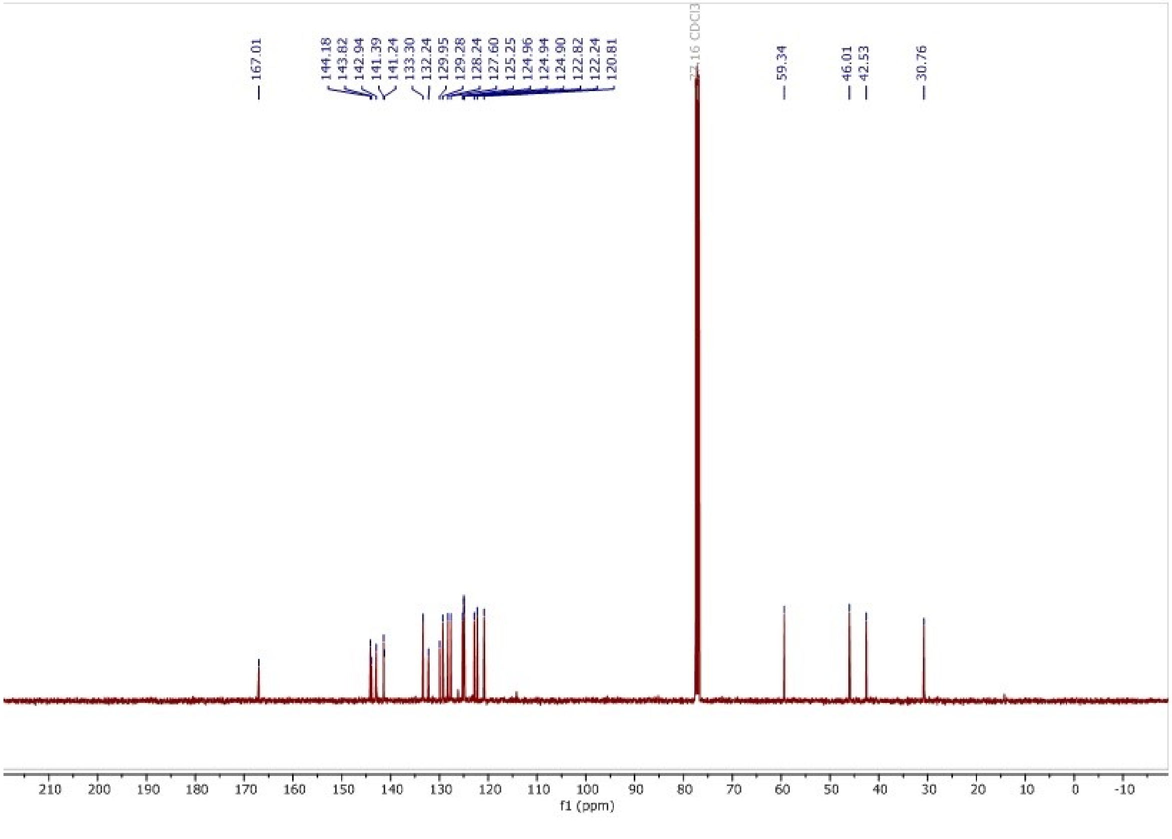

***Trans*-9-bromo-10b,15,15a,15b-tetrahydro-5H-indeno[2,1-c]isoindolo[2,1-a]quinolin-5-one (19)**.

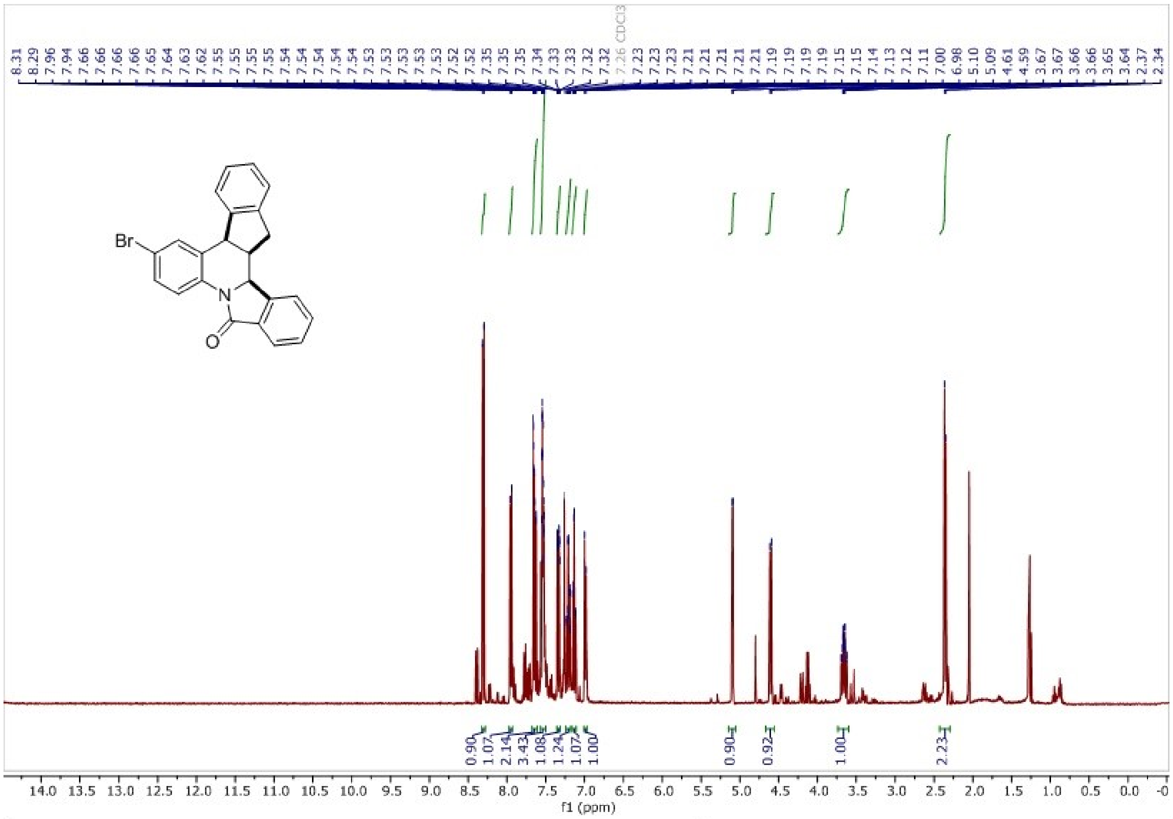

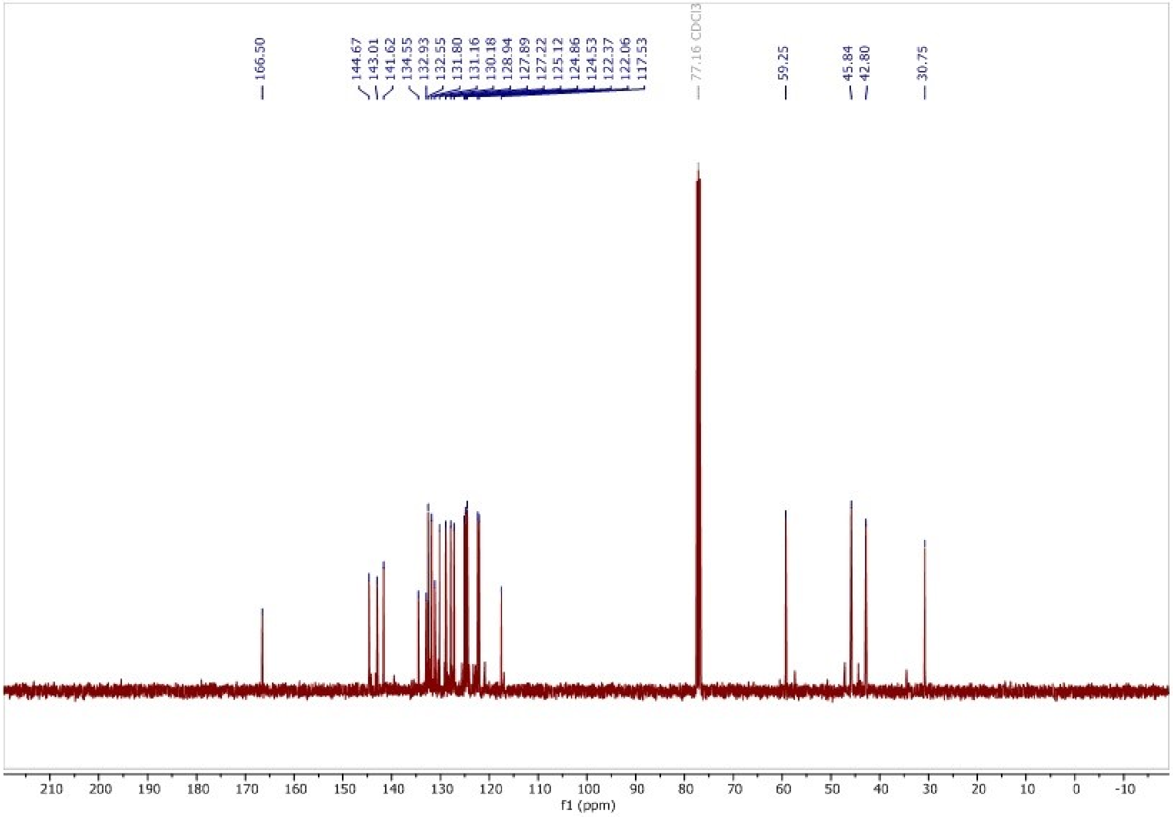

***Trans*-8,9,10-trimethoxy-10b,15,15a,15b-tetrahydro-5H-indeno[2,1-c]isoindolo[2,1-a]quinolin-5-one (20)**.

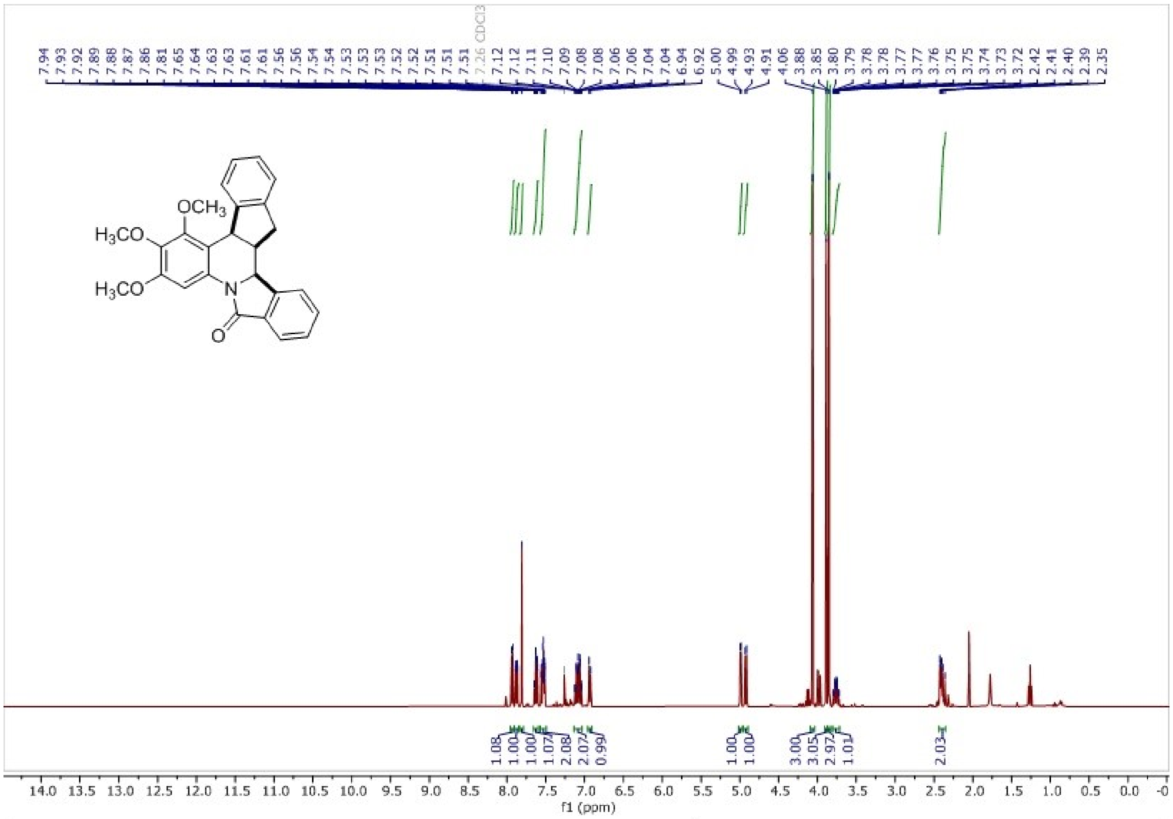

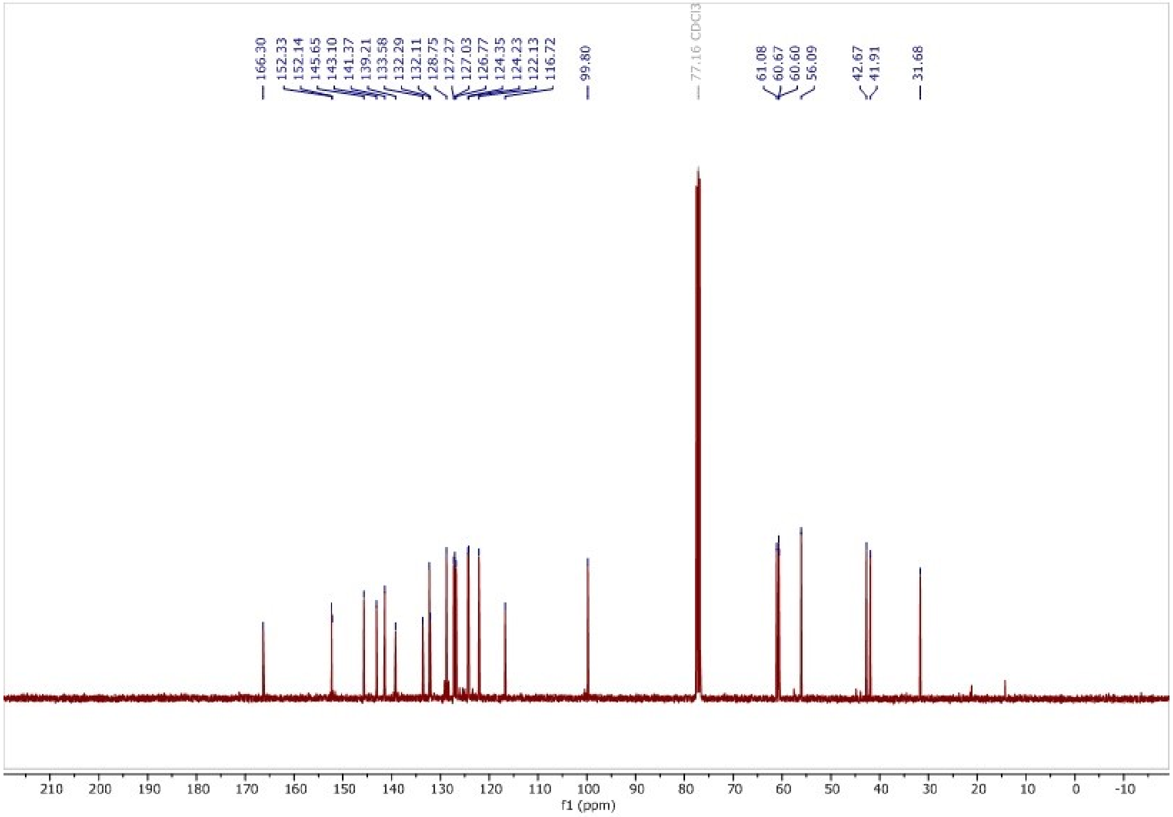

3-hydroxy-2-(p-tolyl)isoindolin-1-one (21).

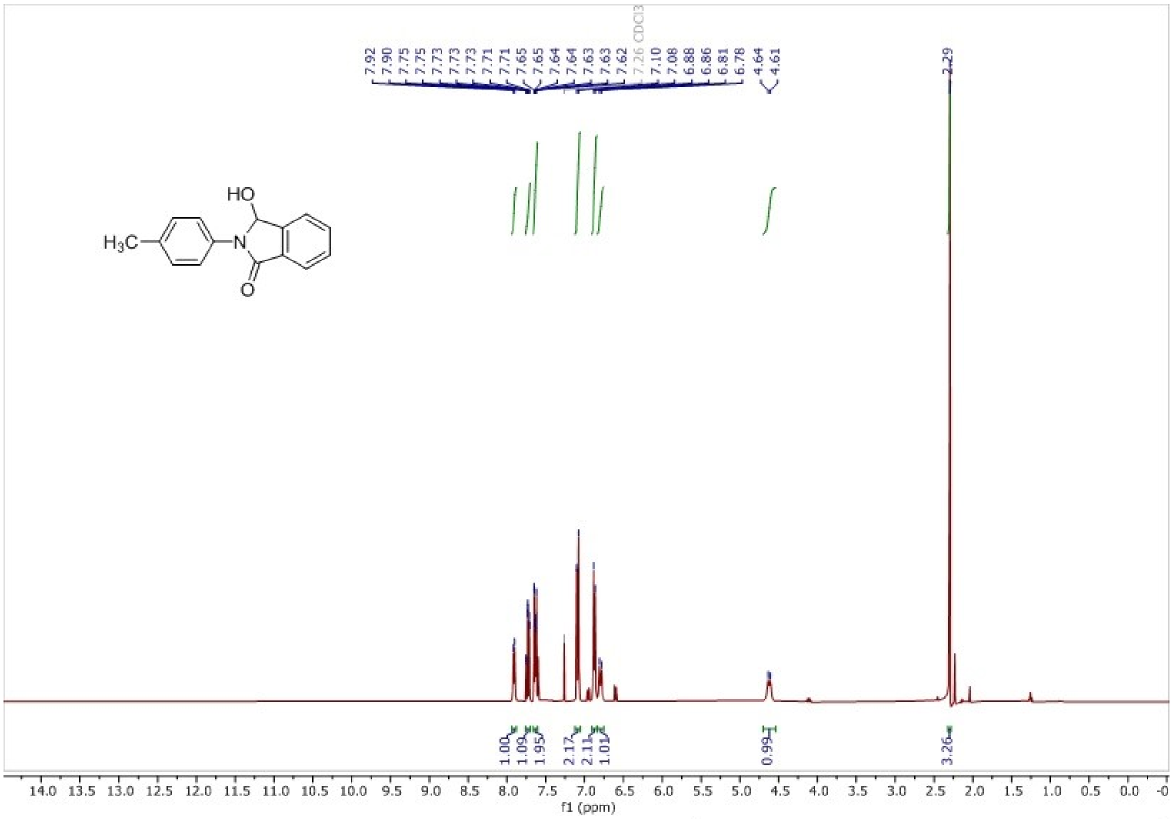

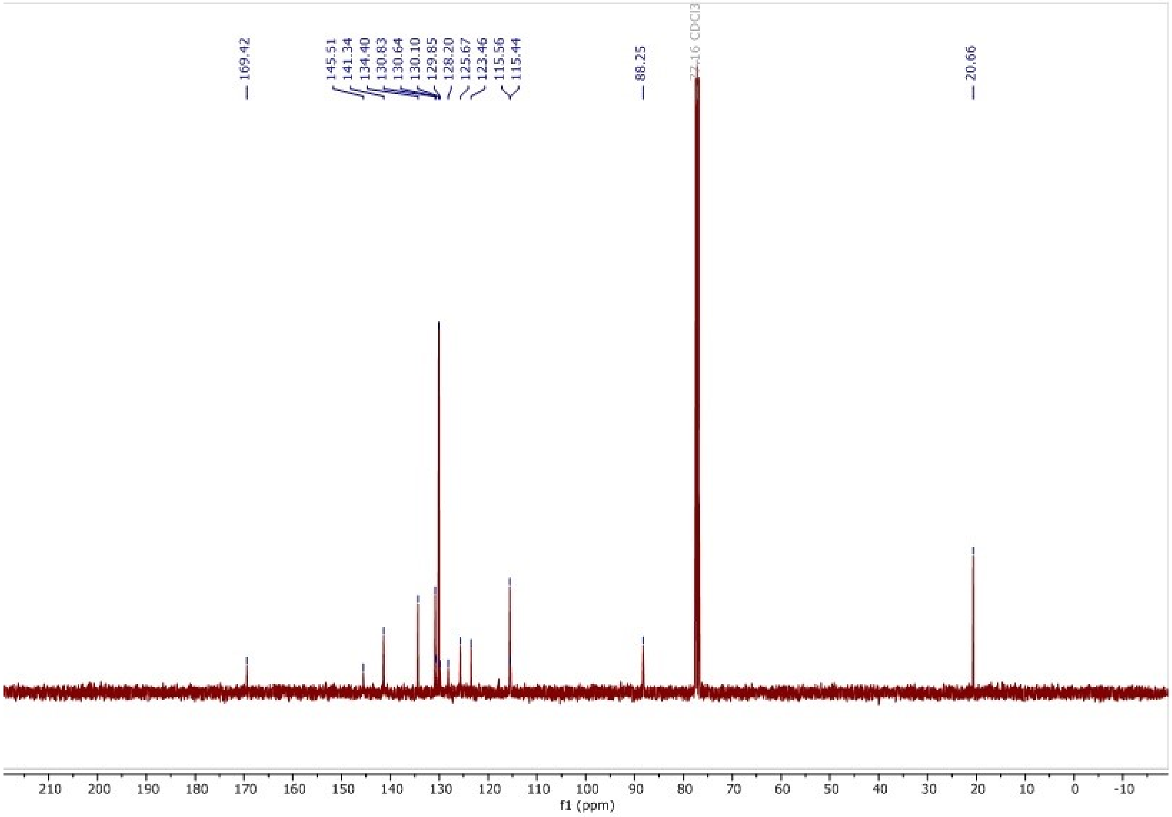

2-(2-methoxyphenyl)-3-((2-methoxyphenyl)amino)isoindolin-1-one (22).

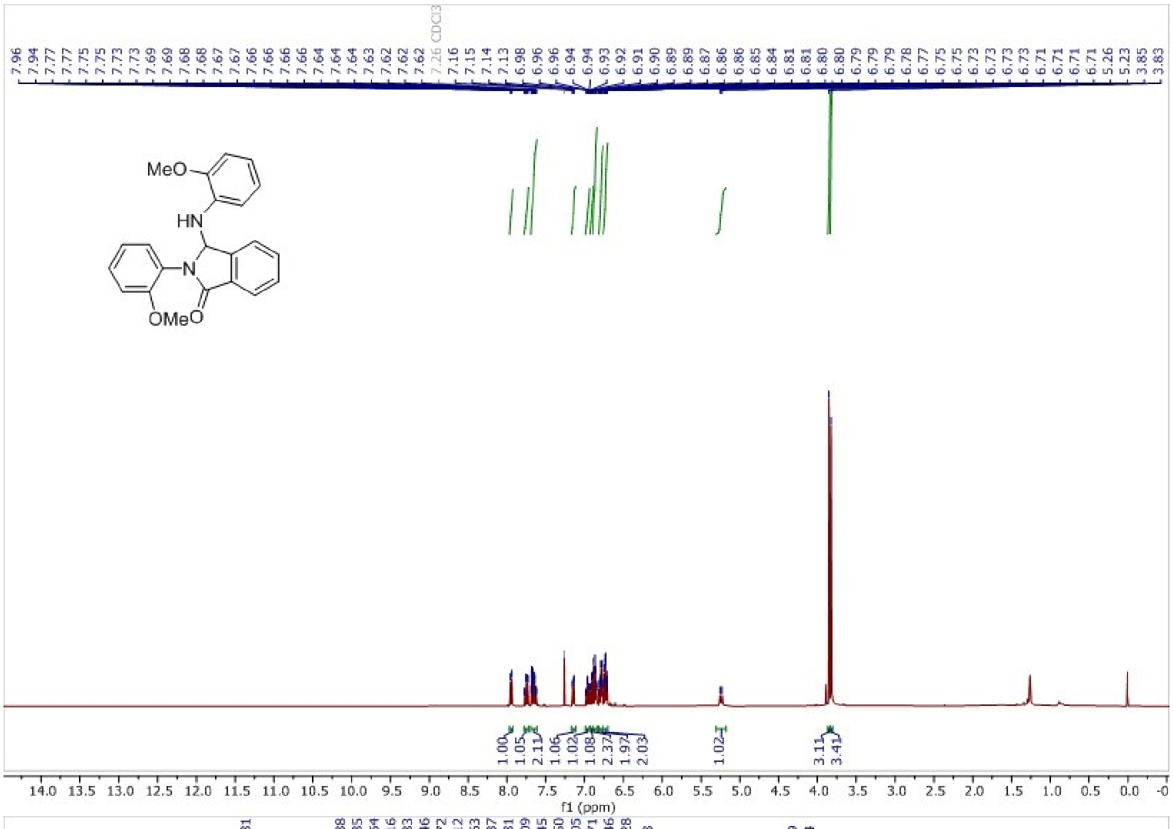

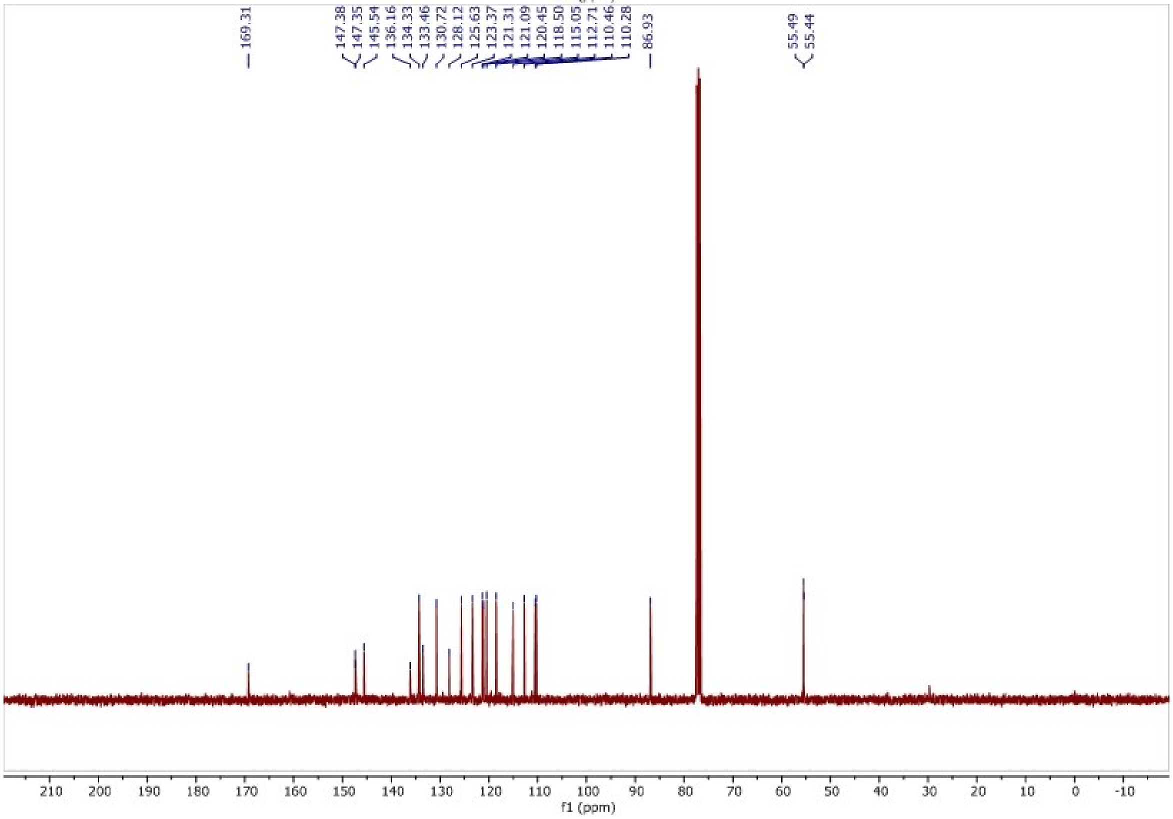

2-(2,4-dimethoxyphenyl)-3-((2,4-dimethoxyphenyl)amino)isoindolin-1-one (23).

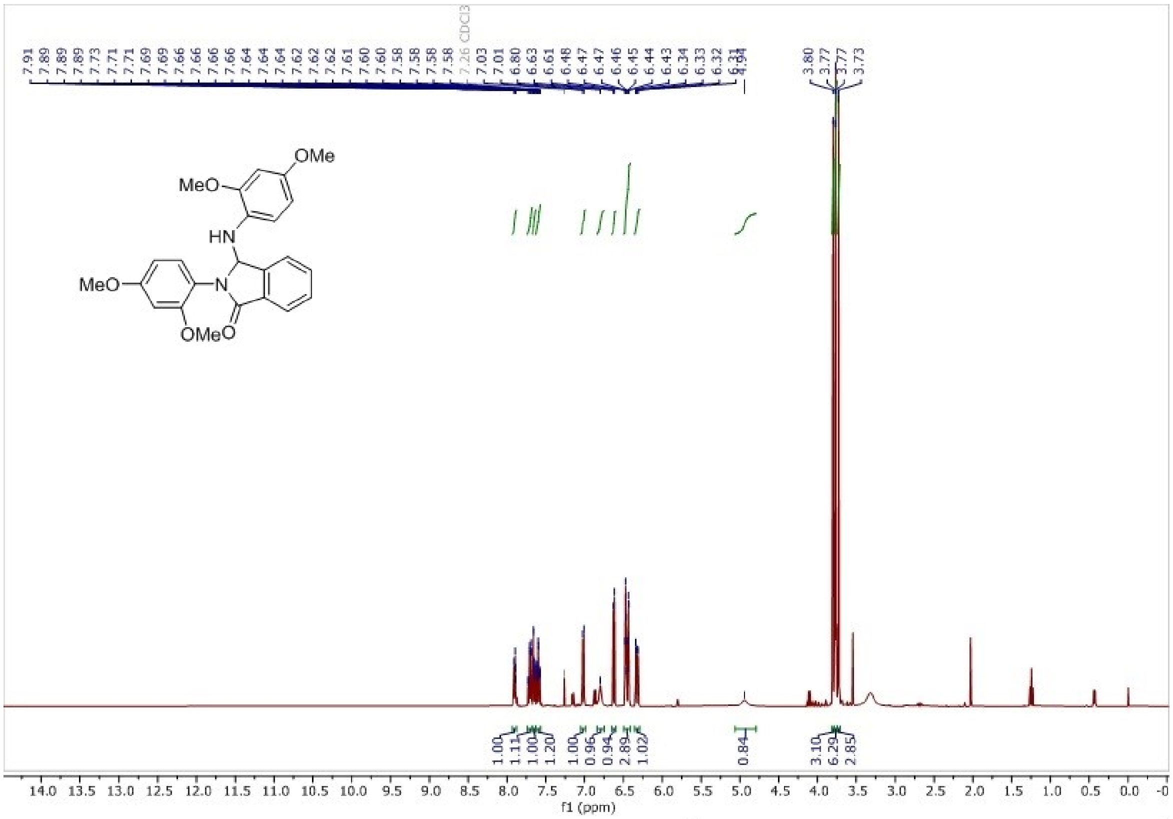
OMe
MeO
HN
MeO
N
OMe O
CDCl3
f1 (ppm)

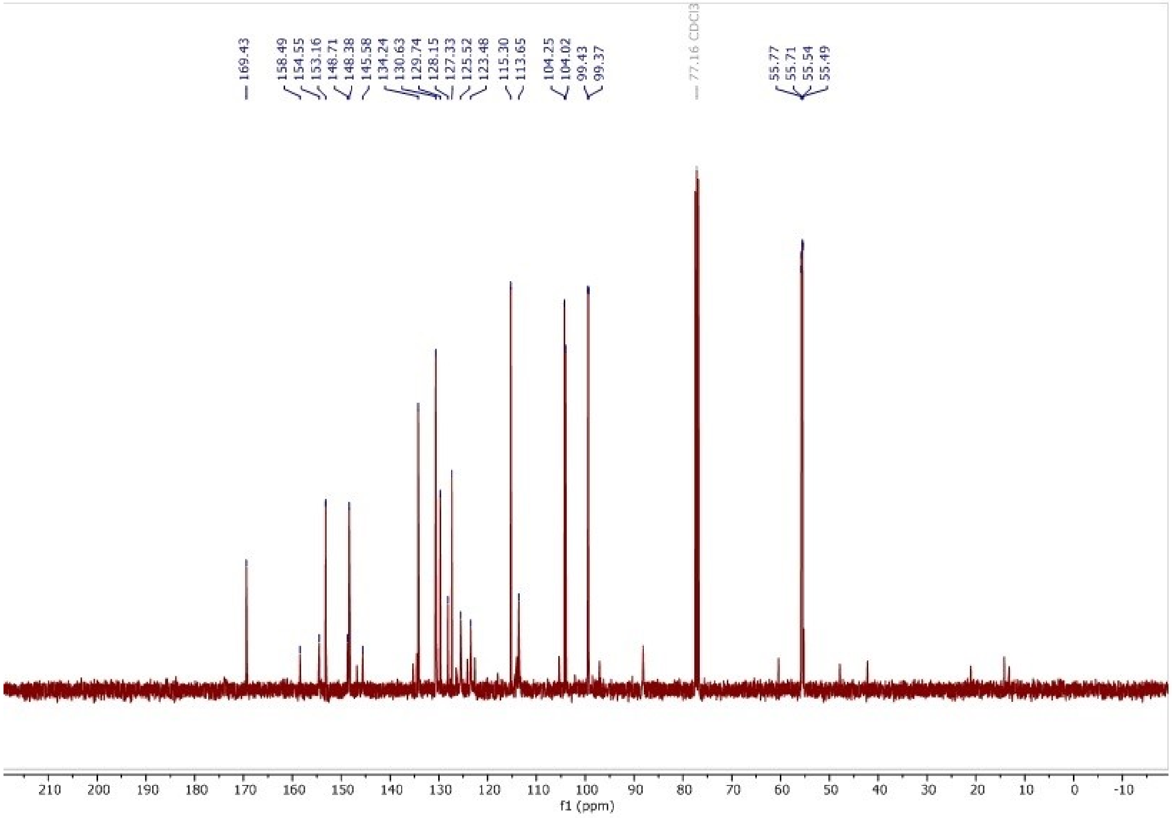
169.43
158.49
154.55
153.16
148.71
148.38
145.58
134.24
130.63
129.74
128.15
127.33
125.52
123.48
115.30
113.65
104.25
104.02
99.43
99.37
77.16 CDCl3
55.77
55.71
55.54
55.49
f1 (ppm)

REFERENCES

1. Merchán-Arenas, D. R.; Kouznetsov, V. V. *J. Org. Chem.* 2014, 79, 5327-5333

2. Wu, C.; Wang, J.; Zhang, X.; Zhang, R.; Ma, B. *Org. Chem. Front.***,** 2021, 8, 6530-6534
